# Association between Guillain–Barré syndrome and 7 autoimmune diseases: a mendelian randomization study

**DOI:** 10.1186/s12883-026-04957-8

**Published:** 2026-05-09

**Authors:** Jialei Zhou, Tong Wang, Gang Chen, Siyuan Yang, Tianyi Wang, Xiang Li

**Affiliations:** Department of Neurosurgery & Brain and Nerve Research Laboratory, First Affiliated Hospital of Soochow University, Suzhou, Jiangsu Province China

**Keywords:** Guillain–Barré syndrome, Autoimmune diseases, Mendelian randomization

## Abstract

**Background:**

The genetic and environmental factors influencing an individual’s susceptibility to develop Guillain–Barré syndrome (GBS) remain largely unidentified. This study aims to evaluate the genetically predicted associations between GBS and candidate autoimmune diseases using a Mendelian randomization (MR) framework.

**Methods:**

Genetic instruments for autoimmune diseases were extracted from large-scale genome-wide association study (GWAS) datasets. Following stringent quality control, seven autoimmune diseases were ultimately retained for the primary analysis. Summary statistics for GBS were obtained from the FinnGen consortium. We employed the inverse variance weighted (IVW) method as the primary approach, supplemented by sensitivity analyses to ensure robustness. Furthermore, a high-throughput two-step MR screening of 731 immune traits was integrated to explore potential biological mediators.

**Results:**

Genetic liability to type 1 diabetes was suggestively associated with an increased risk of GBS [OR (95% CI) = 1.26 (1.04–1.52), *p* = 0.0176]. Conversely, genetic predisposition to psoriasis vulgaris showed a suggestive protective association [OR (95% CI) = 0.784 (0.623–0.986), *p* = 0.0375]. No significant or suggestive associations were observed for rheumatoid arthritis (*p* = 0.990), sarcoidosis (*p* = 0.993), systemic lupus erythematosus (*p* = 0.240), asthma (*p* = 0.906), or Graves’ disease (*p* = 0.926). No circulating immune trait emerged as a significant mediator between these conditions.

**Conclusion:**

This study identifies suggestive genetically predicted associations between specific autoimmune diseases and GBS susceptibility, specifically a potential risk-increasing effect of type 1 diabetes and a protective effect of psoriasis vulgaris. The absence of significant peripheral immune mediators suggests that these neuro-immune interactions may be driven by more localized or complex tissue-resident mechanisms.

**Supplementary Information:**

The online version contains supplementary material available at 10.1186/s12883-026-04957-8.

## Introduction

Guillain–Barré syndrome (GBS) is a group of acute immune-mediated disorders affecting the peripheral nervous system [1]. It stands as the leading cause of acute flaccid paralysis, with an overall annual incidence ranging from 1.1 to 1.8 cases per 100,000 individuals [2]. Despite advancements in medical and supportive care, the mortality rate of GBS within the first year remains approximately 4%. Additionally, GBS results in severe persistent disability in 14% of patients at one year, while approximately 40% experience loss of full strength, persistent pain, and may require professional changes [3]. However, the genetic and environmental factors influencing an individual’s susceptibility to develop GBS remain unidentified [4].

Like GBS, other autoimmune diseases frequently arise from the immune system’s response to self-antigens, leading to the production of specific antibodies detectable through diagnostic testing [5]. These autoimmune diseases might share numerous common features, encompassing antibody-mediated mechanisms, organ specificity in their pathological effects, and dysregulation of the immune system. Given the rarity of GBS, whether the associations between GBS and other autoimmune diseases are causal is uncertain.

Mendelian randomization (MR) analysis, an emerging statistical method, aims to estimate causal relationships between exposure and outcome by utilizing genetic variants, such as single nucleotide polymorphisms (SNPs), as instrumental variables (IVs) [6]. Genetic variants, being randomly assigned at conception, remain unaffected by reverse causation. In the absence of pleiotropy and population stratification, they offer unconfounded estimates of disease risk [7]. In this study, we utilized a two-sample MR framework to examine the genetically predicted associations between candidate autoimmune diseases and GBS risk. Through a comprehensive quality control process, seven autoimmune conditions were ultimately included for the primary analysis. Furthermore, we integrated a high-throughput screening of 731 immune traits to explore potential mediators of these neuro-immune interactions.

## Methods

### Study design and data sources

**Fig. 1:**
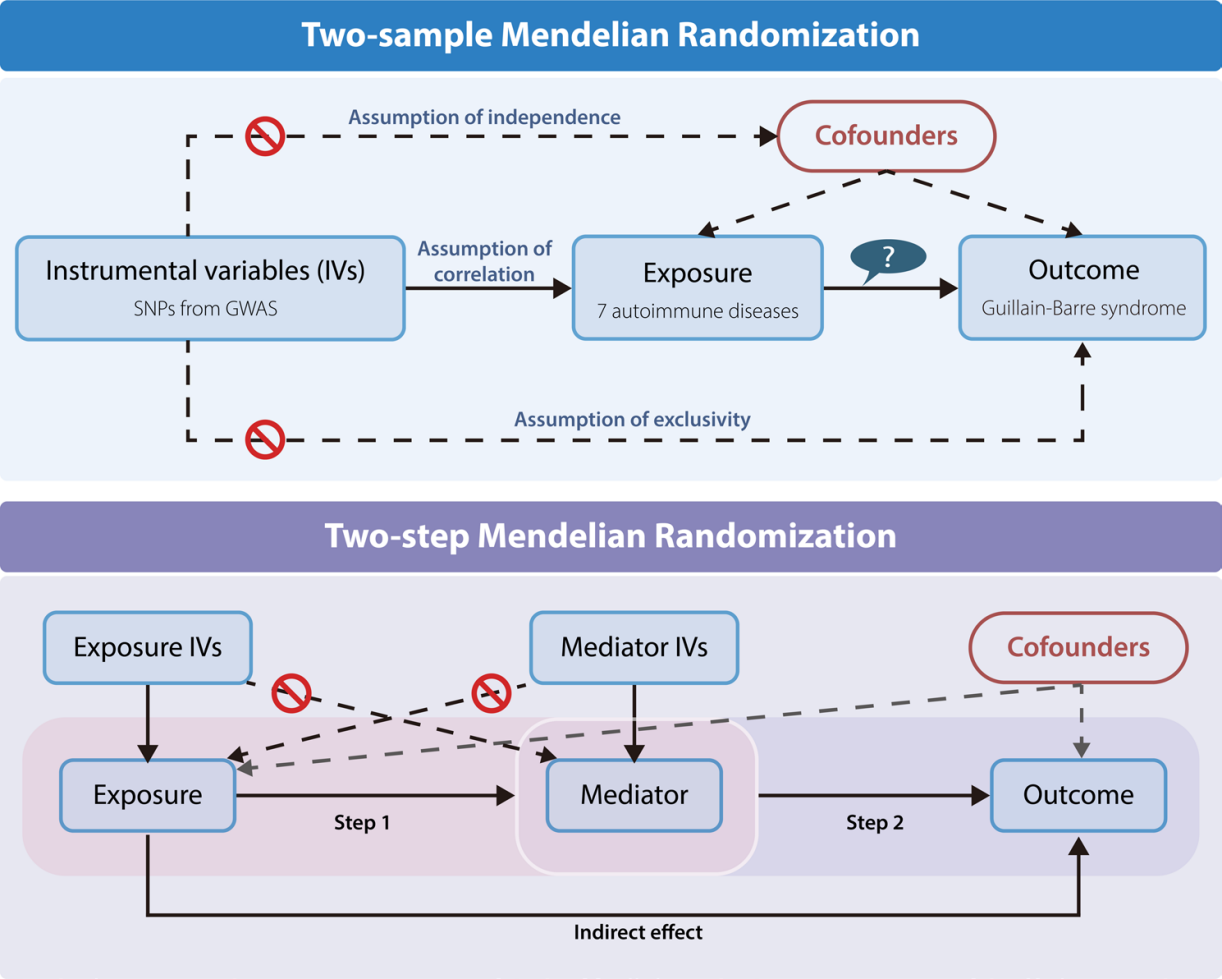
The overview and assumptions of the MR study design. Assumption 1: the instrumental variables that refer to genetic variation have a strong relationship with exposure (autoimmune diseases). Assumption 2: the used IVs are not linked with potential confounders. Assumption 3: the genetic variants are related to the outcome only through selected exposure (autoimmune diseases), not via alternative pathways

Figure 1 shows the overall flow diagram of the Mendelian randomization study examining the relationship between GBS and candidate autoimmune diseases. The research methods strictly adhere to the STROBE-MR guidelines [8]. No additional ethical approval or informed consent was necessary for this study, as all analyses utilized publicly available summary statistics from genome-wide association studies (GWAS) that had previously obtained relevant ethical approvals.

This study employed a two-sample MR framework to infer the potential genetic associations between autoimmune diseases and the risk of GBS. Initially, nine candidate systemic autoimmune diseases were selected based on two primary criteria: (1) characterized by systemic inflammation with known or suspected clinical manifestations involving the peripheral nervous system; and (2) the availability of large-scale, high-quality, European-ancestry GWAS summary statistics to ensure sufficient statistical power. After stringent quality control, seven diseases were ultimately retained for the primary analysis. Summary-level data for the GBS outcome were obtained from the FinnGen consortium, which primarily maps to the International Classification of Diseases, Tenth Revision (ICD-10) code G61.0. Detailed definitions, sample sizes, and GWAS identifiers for all candidate exposures and the outcome are comprehensively provided in Supplementary Table S1.

Regarding the mediation analysis, the 731 immune cell traits were derived from a comprehensive flow-cytometry-based GWAS of a European population [9]. These traits meticulously encompass absolute cell counts, relative proportions, and the mean fluorescence intensity of surface antigens across various innate and adaptive immune cell subsets.

### Instrument selection

The R package TwoSampleMR was used to identify genetic instruments from the autoimmune disease GWAS datasets [10]. To identify valid genetic instruments, we employed SNPs strongly associated with each exposure (*p* < 5 × 10^− 8^), followed by linkage disequilibrium-based clumping (*r*^*2*^ < 0.001, window size = 10,000 kb).

To maximize instrumental retention for variants absent in the outcome GWAS, we implemented an exhaustive, multi-tier proxy search strategy. We prioritized proxy SNPs exhibiting strong linkage disequilibrium (*r*^*2*^ > 0.8) using the European reference samples from the 1000 Genomes Project via the LDlinkR API [11], supplemented by the OpenGWAS proxy database. Ambiguous or palindromic SNPs with nonconcordant alleles or ambiguous strands were systematically excluded from the analysis.

To assess the strength of the instrumental variables (IVs) and mitigate bias from weak instruments, *F*-statistics were computed; IVs were considered robust and retained only if the *F*-statistic was > 10 [12]. Furthermore, Steiger directionality filtering was rigorously applied to remove variants that explained more variance in the outcome than in the exposure, thereby intercepting potential reverse causality at the instrument level [13].

### Statistical analysis

We evaluated the genetic associations between GBS and the seven retained autoimmune diseases using a structured hierarchy of MR methods. The inverse-variance weighted (IVW) method was utilized as the primary statistical approach for interpreting main effects. To ensure robustness and rigorously evaluate potential violations of MR assumptions, we performed secondary sensitivity analyses including MR-Egger regression, weighted median (WM), and weighted mode. The reported odds ratios (ORs) represent the relative risk of GBS per unit increase in the log-odds of genetic liability to the binary autoimmune exposures. Leave-one-out analyses were conducted to assess whether the MR results were driven by any particular variant. Additionally, Cochran’s Q statistic was calculated to evaluate the evidence of heterogeneity, and forest and funnel plots were generated to visually examine heterogeneity and directional pleiotropy. Pleiotropy was further assessed and corrected using the Mendelian Randomization Pleiotropy Residual Sum and Outlier (MR-PRESSO) global test and local outlier detection [14].

To address concerns regarding potential participant overlap between the IEU OpenGWAS exposures and the FinnGen outcome, we calculated the expected sample overlap bias using the quantitative formulation proposed by Burgess et al. [15]. To definitively rule out reverse causality, a reverse MR analysis was systematically conducted using GBS as the exposure (*p* < 5 × 10^− 6^ to acquire sufficient IVs for the relatively small GBS cohort) against the autoimmune traits.

We adopted two-step Mendelian randomization (TSMR) to differentiate the direct and indirect effects of autoimmune diseases on GBS. The indirect mediating effect was determined by the product of the causal effect of the exposure on the mediator (*β*_EM_) and the causal effect of the mediator on GBS (*β*_MO_). This exploratory high-throughput screening utilized 731 immune traits as potential mediators.

To rigorously account for multiple testing, a conservative Bonferroni correction was implemented for the primary MR analysis across the seven retained autoimmune diseases, with strong evidence of association defined as *p* < 0.007 (0.05 / 7). For the TSMR mediation analysis, statistical significance was stringently established using the False Discovery Rate (FDR) correction (Benjamini-Hochberg procedure) to control for Type I error inflation across the 731 tests.

## Results

### Instrumental variables and quantitative bias assessment

Following the implementation of our stringent quality control pipeline (enforcing *F*-statistic > 10, Steiger directionality filtering, and conservative proxy matching), two of the initial nine candidate exposures—Crohn’s disease and ulcerative colitis—were completely excluded due to an insufficient number of directionally robust genetic instruments. Consequently, our final Mendelian randomization (MR) analysis retained seven systemic autoimmune diseases.

For the retained exposures, we identified 12, 23, 5, 5, 18, 22, and 24 independent and significant SNPs as instrumental variables for psoriasis vulgaris, rheumatoid arthritis (RA), sarcoidosis, systemic lupus erythematosus (SLE), type 1 diabetes (T1D), asthma, and Graves’ disease, respectively. Detailed characteristics of all harmonized SNPs are cataloged in Supplementary Table S2. Notably, the mean *F*-statistics for all retained exposure instruments were exceptionally high (ranging from 45.5 to 198), confirming the absence of weak instrument bias. Furthermore, the quantitative assessment of potential sample overlap between the IEU OpenGWAS and FinnGen datasets yielded an expected overlap bias of less than 0.001 for all seven exposures (ranging from 5.05 × 10^− 5^ to 2.20 × 10^− 4^), demonstrating that participant overlap had a negligible impact on our estimates.

### Genetically predicted associations between autoimmune diseases and GBS

**Fig. 2:**
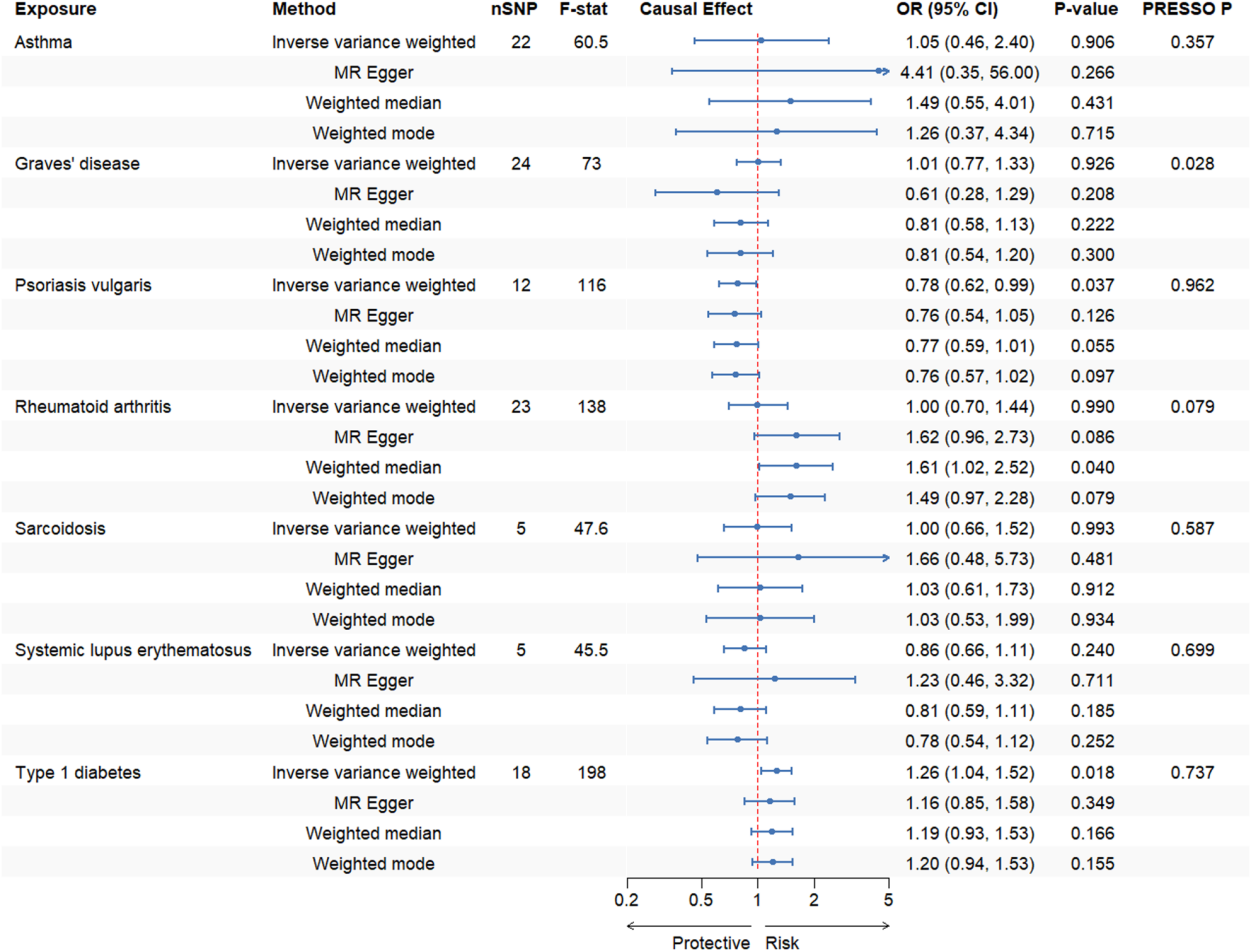
MR analyses effect estimates for associations between seven autoimmune diseases and Guillain–Barré syndrome

The genetically predicted associations inferred from the primary IVW analysis between the seven autoimmune diseases and GBS are summarized in Table 1; Fig. 2. After applying the conservative Bonferroni correction for multiple testing (threshold *p* < 0.007), no autoimmune disease reached the criteria for a definitive causal effect. However, we identified two suggestive genetically predicted associations (0.007 < *p* < 0.05).

**Table 1:** Genetically predicted associations between autoimmune diseases and Guillain-Barré syndrome

Exposure	No. of SNPs	OR (95% CI)	*P*_IVW_	*P*_Bonferroni_	*P*_Heterogeneity_	*P*_Pleiotropy_	*P*_PRESSO_	Mean F
Type 1 diabetes	18	1.26 (1.04–1.52)	0.0176	0.123	0.685	0.538	0.737	198.0
Psoriasis vulgaris	12	0.784 (0.62–0.99)	0.0375	0.263	0.959	0.764	0.962	116.0
SLE	5	0.856 (0.66–1.11)	0.240	1.000	0.709	0.513	0.699	45.5
Asthma	22	1.05 (0.46–2.40)	0.906	1.000	0.087	0.256	0.357	60.5
Graves’ disease	24	1.01 (0.77–1.33)	0.926	1.000	0.037	0.168	0.028	73.0
RA	23	1.00 (0.70–1.44)	0.990	1.000	0.080	0.032	0.079	138.0
Sarcoidosis	5	1.00 (0.66–1.52)	0.993	1.000	0.546	0.458	0.587	47.6

Specifically, genetic liability to type 1 diabetes demonstrated a suggestive association with an increased risk of GBS [OR (95% CI) = 1.26 (1.04–1.52), *p* = 0.0176; Bonferroni *p* = 0.123]. Conversely, genetic predisposition to psoriasis vulgaris exhibited a suggestive protective association against GBS [OR (95% CI) = 0.784 (0.623–0.986), *p* = 0.0375; Bonferroni *p* = 0.262]. We did not observe any significant or suggestive associations between GBS and RA (*p* = 0.990), sarcoidosis (*p* = 0.993), systemic lupus erythematosus (*p* = 0.240), asthma (*p* = 0.906), or Graves’ disease (*p* = 0.926).

### Sensitivity analyses and reverse Mendelian randomization

Extensive sensitivity analyses were conducted to evaluate the robustness of the primary IVW estimates. Cochran’s Q statistic revealed no significant heterogeneity for the majority of the seven autoimmune traits, although nominal heterogeneity was detected for Graves’ disease (*p* = 0.037). Regarding horizontal pleiotropy, the MR-Egger regression intercepts and MR-PRESSO global tests showed no evidence of directional pleiotropy for the primary suggestive exposures—type 1 diabetes and psoriasis vulgaris. While nominal pleiotropic signals were observed for RA and Graves’ disease, visual inspection of the funnel plots and leave-one-out sensitivity plots generally corroborated the stability of the overall findings, showing largely symmetric distributions and confirming that no single variant disproportionately drove the genetic association estimates (Supplementary Figures).

To address concerns regarding potential reverse causality, a reverse MR screening was conducted treating GBS as the exposure against the seven autoimmune outcomes. The analysis revealed no significant reverse associations (*p*-values ranging from 0.150 to 0.888), robustly validating the unidirectional nature of our forward MR findings (Supplementary Table S3).

### High-throughput immune mediation screening

To investigate whether specific circulating immune cell phenotypes mediate the suggestive genetic cross-talk between T1D, psoriasis, and GBS, we executed an unbiased, high-throughput two-step MR screen encompassing 731 distinct immune traits. While several immune traits exhibited nominal unadjusted *p*-values < 0.05, rigorous multiple testing correction via the FDR approach revealed a global null finding. No single immune trait survived the FDR correction (Supplementary Table S4).

## Discussion

The etiology of GBS involves a complex interaction between environmental triggers and host susceptibility. While previous epidemiological studies have extensively documented the roles of preceding infections [16], vaccinations [17], malignancies [18, 19], and surgical procedures [20] in triggering GBS, the specific contribution of the host’s underlying autoimmune background remains incompletely understood. Recently, high-quality Mendelian randomization frameworks have successfully decoded complex genetic axes in various immune-mediated conditions, such as the gut-microbiota-eye axis in acute anterior uveitis [21]. In the present study, we utilized a Mendelian randomization (MR) framework to investigate the genetically predicted associations between a spectrum of systemic autoimmune diseases and GBS risk. Our findings suggest that genetic liability to T1D is associated with an increased risk of GBS, whereas a genetic predisposition to psoriasis vulgaris exhibits a suggestive protective effect.

The observation that genetically predicted psoriasis vulgaris may confer a protective effect against GBS presents an intriguing biological scenario. Clinically, the co-occurrence of psoriasis and GBS is exceedingly rare, with most existing literature limited to isolated case reports, such as instances of GBS induced by biologic therapies (e.g., ixekizumab) in psoriasis patients [22, 23]. Our MR results suggest that the rarity of this comorbidity might be rooted in a shared, yet divergent, genetic architecture. Psoriasis is fundamentally a T-cell mediated inflammatory dermatosis predominantly driven by the IL-23/Th17 axis [24]. In contrast, the pathogenesis of GBS—particularly the acute inflammatory demyelinating polyneuropathy (AIDP) subtype, which constitutes the vast majority of cases in European populations—is largely orchestrated by macrophage-mediated demyelination and a specific humoral response involving autoantibodies against peripheral nerve glycolipids [4]. A potential explanation for our findings is the concept of immune deviation. Genetic variants that strongly polarize the immune system towards a skin-directed Th17 response might simultaneously downregulate or competitively suppress the distinct, neuro-directed humoral pathways required to initiate GBS. Alternatively, this inverse relationship could reflect pleiotropic effects within the human leukocyte antigen (HLA) complex, where certain alleles conferring susceptibility to dermatological autoimmunity simultaneously confer protection against the specific molecular mimicry mechanisms that target the peripheral nerves.

Conversely, our analysis indicated that genetic liability to T1D is suggestively associated with an increased risk of developing GBS. T1D is characterized by the autoimmune destruction of pancreatic beta cells, leading to a state of chronic systemic inflammation [25]. The pathogenesis of T1D involves the sustained upregulation of circulating pro-inflammatory cytokines, including interleukin (IL)-1β, IL-6, and tumor necrosis factor-α (TNF-α) [26]. In the context of the peripheral nervous system, chronic exposure to this pro-inflammatory milieu can compromise the structural and functional integrity of the blood-nerve barrier (BNB) [27]. Increased BNB permeability facilitates the transmigration of circulating autoantibodies, macrophages, and autoreactive T-cells into the endoneurial microenvironment, thereby lowering the threshold for GBS onset following an environmental trigger. Furthermore, it is plausible that shared epitopes between pancreatic islet autoantigens and peripheral nerve antigens (such as Schwann cell surface molecules or myelin proteins) could facilitate cross-reactive neuro-immunity via molecular mimicry [28]. This genetic susceptibility is also consistent with clinical observations demonstrating that hyperglycemia and poor glycemic control are associated with greater clinical severity and poorer short-term prognoses in GBS patients [29].

A critical aspect of our study is the interpretation of the null findings. We did not observe significant genetic associations between GBS and several major autoimmune conditions, including RA, SLE, sarcoidosis, asthma, and Graves’ disease. While previous observational cohort studies have reported an elevated risk of GBS among patients with RA and SLE [30], our MR analysis suggests that these previously reported associations might not be driven by a shared genetic etiology. Instead, the increased incidence of GBS in these patient populations is likely attributable to environmental confounding factors, such as an increased susceptibility to acute respiratory or gastrointestinal infections secondary to the disease itself, or the use of systemic immunosuppressive therapies.

Similarly, our analysis did not find evidence supporting a genetic association between GBS and inflammatory bowel diseases (IBD). Although clinical case reports have frequently linked Crohn’s disease and ulcerative colitis to GBS [31, 32], these traits did not yield sufficient valid genetic instruments to establish a causal relationship in our analysis. The clinical co-occurrence of IBD and GBS may be more accurately explained by shared exposure to enteric pathogens, such as *Campylobacter jejuni*, which is a well-established trigger for GBS and can also exacerbate intestinal inflammation, rather than an underlying genetic pleiotropy.

To further elucidate the pathways linking T1D and psoriasis to GBS, we performed an exploratory mediation analysis incorporating 731 circulating immune cell traits. Following appropriate correction for multiple testing, no single peripheral immune trait emerged as a significant mediator. This finding suggests that the genetic cross-talk between these conditions is unlikely to be mediated by simple numerical shifts or surface marker expressions of circulating immune cells. Rather, the neuro-immune interactions dictating GBS susceptibility are likely localized, potentially involving tissue-resident macrophages within the peripheral nerves or specific Schwann cell-immune interactions that cannot be captured by broad peripheral blood immunophenotyping.

This study has several strengths. To our knowledge, it is the first to systematically evaluate the genetic associations between a wide array of systemic autoimmune diseases and GBS risk. The two-sample MR design inherently minimizes the biases of environmental confounding and reverse causation that frequently limit observational studies. However, several limitations must be acknowledged. First, the relatively small sample size of the GBS cohort in the FinnGen dataset limits our statistical power, meaning our findings should be interpreted as suggestive and require replication in larger, multi-ancestry meta-analyses. Second, as with all MR studies, we cannot entirely rule out the influence of unmeasured horizontal pleiotropy. Third, the reliance on the overarching ICD-10 diagnostic code (G61.0) for GBS precludes subtype-specific analyses. Given that our data is derived from European populations, these results primarily reflect the genetic architecture of the acute inflammatory demyelinating polyneuropathy (AIDP) subtype and may not be generalizable to axonal variants which possess distinct pathophysiological mechanisms.

## Conclusions

In conclusion, our Mendelian randomization study provides evidence for suggestive genetically predicted associations between specific autoimmune diseases and the risk of GBS. Specifically, we identified a potential risk-increasing association with type 1 diabetes and a protective association with psoriasis vulgaris. The absence of genetic associations with other major autoimmune diseases highlights the specific and complex nature of the neuro-immune axis in GBS. Future large-scale genetic and experimental studies are warranted to validate these findings and to unravel the precise barrier-disruption and molecular mimicry mechanisms linking systemic autoimmunity to peripheral nerve demyelination.

## Supplementary Information

Supplementary Material 1. Table S1.

Supplementary Material 2. Table S2.

Supplementary Material 3. Table S3.

Supplementary Material 4. Table S4.

Supplementary Material 5. Supplementary Figures.

## Data Availability

The GWAS summary data for psoriasis vulgaris, rheumatoid arthritis, sarcoidosis, systemic lupus erythematosus, type 1 diabetes, asthma, Crohn’s disease, ulcerative colitis, Graves’ disease and GBS were obtained from the GWAS catalog website (https://gwas.mrcieu.ac.uk).

## References

[1] Liu S, Zhang WW, Jia L, Zhang H-L. Guillain-Barré syndrome: immunopathogenesis and therapeutic targets. Expert Opin Ther Targets. 2024;1–13. 10.1080/14728222.2024.2330435. 10.1080/14728222.2024.2330435 38470316

[2] McGrogan A, Madle GC, Seaman HE, De Vries CS. The epidemiology of Guillain-Barré Syndrome worldwide. Neuroepidemiology. 2009;32:150–63. 10.1159/000184748. 19088488 10.1159/000184748

[3] Rajabally YA, Uncini A. Outcome and its predictors in Guillain–Barré syndrome. J Neurol Neurosurg Psychiatry. 2012;83:711–8. 10.1136/jnnp-2011-301882. 22566597 10.1136/jnnp-2011-301882

[4] Willison HJ, Jacobs BC, Van Doorn PA. Guillain-Barré syndrome. Lancet. 2016;388:717–27. 10.1016/S0140-6736(16)00339-1. 26948435 10.1016/S0140-6736(16)00339-1

[5] Li K, Ouyang Y, Yang H. Myasthenia gravis and five autoimmune diseases: a bidirectional mendelian randomization study. Neurol Sci Off J Ital Neurol Soc Ital Soc Clin Neurophysiol. 2023. 10.1007/s10072-023-07163-3. 10.1007/s10072-023-07163-3 37910321

[6] Bowden J, Holmes MV. Meta-analysis and Mendelian randomization: a review. Res Synth Methods. 2019;10:486–96. 10.1002/jrsm.1346. 30861319 10.1002/jrsm.1346 PMC6973275

[7] Burgess S, Butterworth A, Thompson SG. Mendelian randomization analysis with multiple genetic variants using summarized data. Genet Epidemiol. 2013;37:658–65. 10.1002/gepi.21758. 24114802 10.1002/gepi.21758 PMC4377079

[8] Skrivankova VW, Richmond RC, Woolf BAR, Yarmolinsky J, Davies NM, Swanson SA, et al. Strengthening the reporting of observational studies in epidemiology using mendelian randomization: the STROBE-MR statement. JAMA. 2021;326:1614. 10.1001/jama.2021.18236. 34698778 10.1001/jama.2021.18236

[9] Orrù V, Steri M, Sidore C, Marongiu M, Serra V, Olla S, et al. Complex genetic signatures in immune cells underlie autoimmunity and inform therapy. Nat Genet. 2020;52:1036–45. 10.1038/s41588-020-0684-4. 32929287 10.1038/s41588-020-0684-4 PMC8517961

[10] Hemani G, Zheng J, Elsworth B, Wade KH, Haberland V, Baird D, et al. The MR-Base platform supports systematic causal inference across the human phenome. eLife. 2018;7:e34408. 10.7554/eLife.34408. 29846171 10.7554/eLife.34408 PMC5976434

[11] Myers TA, Chanock SJ, Machiela MJ. LDlinkR: An R package for rapidly calculating linkage disequilibrium statistics in diverse populations. Front Genet. 2020;11:157. 10.3389/fgene.2020.00157. 32180801 10.3389/fgene.2020.00157 PMC7059597

[12] Burgess S, Thompson SG, CRP CHD Genetics Collaboration. Avoiding bias from weak instruments in mendelian randomization studies. Int J Epidemiol. 2011;40:755–64. 10.1093/ije/dyr036. 21414999 10.1093/ije/dyr036

[13] Hemani G, Tilling K, Davey Smith G. Orienting the causal relationship between imprecisely measured traits using GWAS summary data. PLoS Genet. 2017;13:e1007081. 10.1371/journal.pgen.1007081. 29149188 10.1371/journal.pgen.1007081 PMC5711033

[14] Verbanck M, Chen C-Y, Neale B, Do R. Detection of widespread horizontal pleiotropy in causal relationships inferred from Mendelian randomization between complex traits and diseases. Nat Genet. 2018;50:693–8. 10.1038/s41588-018-0099-7. 29686387 10.1038/s41588-018-0099-7 PMC6083837

[15] Burgess S, Davies NM, Thompson SG. Bias due to participant overlap in two-sample mendelian randomization. Genet Epidemiol. 2016;40:597–608. 10.1002/gepi.21998. 27625185 10.1002/gepi.21998 PMC5082560

[16] Levison LS, Thomsen RW, Sindrup SH, Andersen H. Association of hospital-diagnosed infections and antibiotic use with risk of developing Guillain-Barré Syndrome. Neurology. 2021;96. 10.1212/WNL.0000000000011342. 10.1212/WNL.0000000000011342 33318166

[17] Levison LS, Thomsen RW, Andersen H. Guillain–Barré syndrome following influenza vaccination: a 15-year nationwide population‐based case–control study. Eur J Neurol. 2022;29:3389–94. 10.1111/ene.15516. 35913431 10.1111/ene.15516 PMC9804417

[18] Goodfellow JA, Willison HJ. Guillain–Barré syndrome: a century of progress. Nat Rev Neurol. 2016;12:723–31. 10.1038/nrneurol.2016.172. 27857121 10.1038/nrneurol.2016.172

[19] Levison LS, Thomsen RW, Sindrup SH, Andersen H. Association between incident cancer and guillain-barré syndrome development: a nationwide case-control study. Neurology. 2022;98. 10.1212/WNL.0000000000200015. 10.1212/WNL.0000000000200015 35236772

[20] Levison LS, Thomsen RW, Andersen H. Hospital-diagnosed morbidities and recent surgery as risk factors for developing Guillain-Barré syndrome. Eur J Neurol. 2023;30:3277–85. 10.1111/ene.15955. 37368224 10.1111/ene.15955

[21] Mi Y, Chen L, Liao N, Wan M. Mendelian randomization analysis revealed a gut microbiota-eye axis in acute anterior uveitis. Eye. 2025;39:1562–70. 10.1038/s41433-025-03715-3. 39979613 10.1038/s41433-025-03715-3 PMC12089388

[22] Sargın B. Psoriasis and Guillain-Barré Syndrome: incidental or associated? Arch Rheumatol. 2017;32:273–4. 10.5606/ArchRheumatol.2017.6296. 30375542 10.5606/ArchRheumatol.2017.6296 PMC6190954

[23] Liu H, Li Q, Li Q, Li X, Dai L. Guillain–Barré syndrome is associated with ixekizumab in a patient with pustular psoriasis. Rheumatology. 2023;62:e161–3. 10.1093/rheumatology/keac609. 36269188 10.1093/rheumatology/keac609

[24] Greb JE, Goldminz AM, Elder JT, Lebwohl MG, Gladman DD, Wu JJ, et al. Psoriasis. Nat Rev Dis Primer. 2016;2:16082. 10.1038/nrdp.2016.82. 10.1038/nrdp.2016.82 27883001

[25] Suliman BA. Potential clinical implications of molecular mimicry-induced autoimmunity. Immun Inflamm Dis. 2024;12:e1178. 10.1002/iid3.1178. 38415936 10.1002/iid3.1178 PMC10832321

[26] Donath MY, Dalmas É, Sauter NS, Böni-Schnetzler M. Inflammation in obesity and diabetes: islet dysfunction and therapeutic opportunity. Cell Metab. 2013;17:860–72. 10.1016/j.cmet.2013.05.001. 23747245 10.1016/j.cmet.2013.05.001

[27] Ubogu EE. Biology of the human blood-nerve barrier in health and disease. Exp Neurol. 2020;328:113272. 10.1016/j.expneurol.2020.113272. 32142802 10.1016/j.expneurol.2020.113272 PMC7145763

[28] Yuki N, Hartung H-P. Guillain-barré syndrome. N Engl J Med. 2012;366:2294–304. 10.1056/NEJMra1114525. 22694000 10.1056/NEJMra1114525

[29] Gong Q, Liu S, Xiao Z, Fu X, Lu Z. Elevated blood and cerebrospinal fluid glucose levels affect the severity and short-term prognosis of Guillain-Barré syndrome. Neurol Res. 2022;44:121–7. 10.1080/01616412.2021.1965337. 34382919 10.1080/01616412.2021.1965337

[30] Auger N, Quach C, Healy-Profitós J, Dinh T, Chassé M. Early predictors of Guillain-Barré syndrome in the life course of women. Int J Epidemiol. 2018;47:280–8. 10.1093/ije/dyx181. 29024971 10.1093/ije/dyx181 PMC5837790

[31] Bouchra A, Benbouazza K, Hajjaj-Hassouni N. Guillain–Barre in a patient with ankylosing spondylitis secondary to ulcerative colitis on infliximab therapy. Clin Rheumatol. 2009;28:53–5. 10.1007/s10067-009-1154-7. 19277812 10.1007/s10067-009-1154-7

[32] de Azevedo MFC, Queiroz NSF, Damião AOMC. Guillain-barré syndrome in a crohn`s disease patient treated with vedolizumab. J Crohns Colitis. 2020;14:1788–9. 10.1093/ecco-jcc/jjaa101. 32417923 10.1093/ecco-jcc/jjaa101

