# Supplementary material for: Association between Guillain–Barré syndrome and 7 autoimmune diseases: a mendelian randomization study
Source: BMC Neurol. 2026 May 9;26:425. doi: 10.1186/s12883-026-04957-8 (PMC13326399; doi:10.1186/s12883-026-04957-8)
Supplement: Supplementary file 1 — Supplementary Material 1. Table S1. [file 12883_2026_4957_MOESM1_ESM.docx]

| **Disease** | **Sample size** | **Cases** | **Years** | **Population** | **GWAS ID** | **ICD-10 codes** |
| --- | --- | --- | --- | --- | --- | --- |
| Psoriasis vulgaris | 483,174 | 5,072 | 2021 | European | ebi-a-GCST90018907 | L40 |
| Rheumatoid arthritis | 417,256 | 8,255 | 2021 | European | ebi-a-GCST90018910 | M06 |
| Sarcoidosis | 486,673 | 1,718 | 2021 | European | ebi-a-GCST90018918 | D86 |
| Systemic lupus erythematosus | 482,911 | 647 | 2021 | European | ebi-a-GCST90018917 | M32 |
| Type 1 diabetes | 457,695 | 6,447 | 2021 | European | ebi-a-GCST90018925 | E10 |
| Asthma | 449,500 | 38,369 | 2021 | European | ebi-a-GCST90018795 | J45 |
| Graves' disease | 458,620 | 1,678 | 2021 | European | ebi-a-GCST90018847 | E05.0 |
| Guillain–Barré syndrome | 215,931 | 213 | 2021 | European | finn-b-G6_GUILBAR | G61.0 |

Supplementary Table S1: Detailed characteristics of GWAS datasets for candidate autoimmune diseases and Guillain-Barré syndrome.
