# Supplementary material for: Association between Guillain–Barré syndrome and 7 autoimmune diseases: a mendelian randomization study
Source: BMC Neurol. 2026 May 9;26:425. doi: 10.1186/s12883-026-04957-8 (PMC13326399; doi:10.1186/s12883-026-04957-8)
Supplement: Supplementary file 2 — Supplementary Material 2. Table S2. [file 12883_2026_4957_MOESM2_ESM.docx]

| **Exposure** | **SNP ID** | **Effect**  **Allele** | **Other**  **Allele** | **EAF** | **Beta**  **Exposure** | **SE**  **Exposure** | **P_val**  **Exposure** | **Beta**  **Outcome** | **SE**  **Outcome** | **P_val**  **Outcome** | **R2**  **Explained** | **F_Statistic** |
| --- | --- | --- | --- | --- | --- | --- | --- | --- | --- | --- | --- | --- |
| Psoriasis vulgaris | rs10040411 | T | C | 0.368 | -0.237 | 0.0297 | 1.45E-15 | 0.143 | 0.107 | 0.182 | 0.000132 | 63.8 |
| Psoriasis vulgaris | rs1131896 | A | G | 0.281 | -0.227 | 0.0245 | 1.67E-20 | -0.0018 | 0.119 | 0.988 | 0.000178 | 86 |
| Psoriasis vulgaris | rs1177203 | C | G | 0.364 | -0.121 | 0.0205 | 3.77E-09 | 0.0166 | 0.0993 | 0.868 | 7.22E-05 | 34.9 |
| Psoriasis vulgaris | rs12188300 | T | A | 0.0849 | 0.395 | 0.0397 | 2.57E-23 | -0.18 | 0.208 | 0.386 | 0.000205 | 99.1 |
| Psoriasis vulgaris | rs1233621 | G | T | 0.0925 | 0.259 | 0.0392 | 4.04E-11 | -0.0053 | 0.109 | 0.961 | 9.02E-05 | 43.6 |
| Psoriasis vulgaris | rs1233621 | G | T | 0.0925 | 0.259 | 0.0392 | 4.04E-11 | 0.0316 | 0.195 | 0.871 | 9.02E-05 | 43.6 |
| Psoriasis vulgaris | rs2154026 | G | C | 0.0831 | 0.393 | 0.0509 | 1.11E-14 | 0.0316 | 0.195 | 0.871 | 0.000124 | 59.7 |
| Psoriasis vulgaris | rs2249937 | G | T | 0.769 | -0.158 | 0.0233 | 1.18E-11 | -0.0999 | 0.118 | 0.399 | 9.52E-05 | 46 |
| Psoriasis vulgaris | rs2312786 | A | G | 0.231 | 0.14 | 0.0229 | 9.61E-10 | -0.14 | 0.104 | 0.178 | 7.77E-05 | 37.5 |
| Psoriasis vulgaris | rs28894993 | T | G | 0.0624 | 1.34 | 0.0437 | 1.00E-200 | -0.376 | 0.202 | 0.0628 | 0.00195 | 945 |
| Psoriasis vulgaris | rs34725611 | G | A | 0.305 | -0.164 | 0.0225 | 3.80E-13 | 0.001 | 0.111 | 0.993 | 0.000109 | 52.9 |
| Psoriasis vulgaris | rs6672420 | T | A | 0.52 | 0.124 | 0.0201 | 7.01E-10 | 0.0493 | 0.0971 | 0.612 | 7.84E-05 | 37.9 |
| Psoriasis vulgaris | rs6933987 | T | C | 0.225 | 0.126 | 0.0223 | 1.78E-08 | -0.0219 | 0.103 | 0.832 | 6.59E-05 | 31.8 |
| Psoriasis vulgaris | rs9481169 | T | G | 0.0776 | 0.256 | 0.0373 | 6.58E-12 | -0.0263 | 0.183 | 0.886 | 9.75E-05 | 47.1 |
| Rheumatoid arthritis | rs10261758 | A | G | 0.711 | -0.081 | 0.0143 | 1.47E-08 | 0.16 | 0.1 | 0.112 | 7.69E-05 | 32.1 |
| Rheumatoid arthritis | rs10797431 | T | G | 0.404 | -0.074 | 0.0129 | 1.06E-08 | 0.0731 | 0.1 | 0.466 | 7.89E-05 | 32.9 |
| Rheumatoid arthritis | rs11889341 | T | C | 0.25 | 0.123 | 0.0146 | 2.71E-17 | -0.111 | 0.115 | 0.336 | 0.000171 | 71.2 |
| Rheumatoid arthritis | rs142144003 | A | G | 0.111 | 0.413 | 0.0207 | 1.09E-88 | 0.192 | 0.235 | 0.413 | 0.000955 | 399 |
| Rheumatoid arthritis | rs1776616 | G | A | 0.665 | 0.0832 | 0.0139 | 2.41E-09 | -0.0812 | 0.118 | 0.491 | 8.59E-05 | 35.8 |
| Rheumatoid arthritis | rs2156698 | A | G | 0.456 | -0.0745 | 0.0131 | 1.44E-08 | 0.0923 | 0.0974 | 0.343 | 7.75E-05 | 32.3 |
| Rheumatoid arthritis | rs2618444 | C | A | 0.387 | 0.1 | 0.0146 | 6.71E-12 | 0.122 | 0.127 | 0.338 | 0.000114 | 47.4 |
| Rheumatoid arthritis | rs28362859 | C | A | 0.1 | 0.146 | 0.0205 | 9.90E-13 | -0.0753 | 0.199 | 0.705 | 0.000122 | 51.1 |
| Rheumatoid arthritis | rs3093017 | G | C | 0.546 | -0.118 | 0.0128 | 4.34E-20 | -0.009 | 0.0982 | 0.927 | 0.000203 | 84.8 |
| Rheumatoid arthritis | rs34536443 | C | G | 0.0428 | -0.237 | 0.0427 | 3.03E-08 | 0.425 | 0.283 | 0.133 | 7.36E-05 | 30.7 |
| Rheumatoid arthritis | rs35139284 | T | C | 0.319 | 0.539 | 0.0149 | 1.00E-200 | 0.279 | 0.13 | 0.0319 | 0.00312 | 1310 |
| Rheumatoid arthritis | rs35511257 | C | G | 0.0746 | 0.689 | 0.0408 | 4.38E-64 | 0.495 | 0.753 | 0.511 | 0.000683 | 285 |
| Rheumatoid arthritis | rs362531 | A | G | 0.0615 | -0.212 | 0.0252 | 3.47E-17 | 0.423 | 0.599 | 0.48 | 0.00017 | 70.8 |
| Rheumatoid arthritis | rs3757387 | C | T | 0.344 | 0.109 | 0.0147 | 1.47E-13 | 0.0313 | 0.0984 | 0.75 | 0.000131 | 54.6 |
| Rheumatoid arthritis | rs56376587 | C | A | 0.429 | 0.0785 | 0.0133 | 3.77E-09 | 0.0812 | 0.0993 | 0.413 | 8.35E-05 | 34.8 |
| Rheumatoid arthritis | rs5754100 | C | T | 0.244 | 0.086 | 0.015 | 9.20E-09 | -0.224 | 0.107 | 0.0363 | 7.88E-05 | 32.9 |
| Rheumatoid arthritis | rs5757628 | A | G | 0.428 | 0.0966 | 0.0157 | 7.58E-10 | 0.121 | 0.106 | 0.255 | 9.07E-05 | 37.9 |
| Rheumatoid arthritis | rs62395272 | T | C | 0.111 | 0.315 | 0.0206 | 6.60E-53 | -0.19 | 0.155 | 0.221 | 0.000559 | 233 |
| Rheumatoid arthritis | rs6679677 | A | C | 0.113 | 0.305 | 0.0254 | 2.54E-33 | -0.0788 | 0.137 | 0.565 | 0.000346 | 144 |
| Rheumatoid arthritis | rs71508903 | T | C | 0.211 | 0.0872 | 0.0157 | 2.54E-08 | -0.282 | 0.126 | 0.0251 | 7.39E-05 | 30.8 |
| Rheumatoid arthritis | rs7731626 | A | G | 0.279 | -0.134 | 0.0157 | 9.77E-18 | -0.122 | 0.108 | 0.261 | 0.000175 | 73.2 |
| Rheumatoid arthritis | rs9277814 | G | A | 0.51 | 0.132 | 0.0145 | 6.12E-20 | -0.129 | 0.104 | 0.216 | 0.000199 | 83.1 |
| Rheumatoid arthritis | rs9469053 | G | A | 0.0454 | -0.291 | 0.0312 | 1.22E-20 | 0.461 | 0.496 | 0.352 | 0.000208 | 86.8 |
| Rheumatoid arthritis | rs9494894 | C | T | 0.0417 | 0.173 | 0.031 | 2.48E-08 | -0.0478 | 0.361 | 0.895 | 7.43E-05 | 31 |
| Sarcoidosis | rs12563505 | T | C | 0.0327 | 0.703 | 0.11 | 1.68E-10 | 0.0852 | 0.365 | 0.816 | 8.38E-05 | 40.8 |
| Sarcoidosis | rs2245168 | C | T | 0.484 | -0.27 | 0.033 | 2.93E-16 | 0.0145 | 0.0975 | 0.882 | 0.000138 | 66.9 |
| Sarcoidosis | rs28688207 | C | T | 0.0739 | 0.744 | 0.0927 | 1.08E-15 | 0.144 | 0.44 | 0.744 | 0.000132 | 64.3 |
| Sarcoidosis | rs3093958 | G | A | 0.129 | 0.323 | 0.0565 | 1.07E-08 | 0.209 | 0.181 | 0.247 | 6.71E-05 | 32.7 |
| Sarcoidosis | rs72928925 | C | A | 0.143 | 0.252 | 0.0436 | 7.77E-09 | -0.149 | 0.12 | 0.213 | 6.86E-05 | 33.4 |
| Systemic lupus erythematosus | rs1131114 | C | T | 0.179 | 0.423 | 0.0678 | 4.36E-10 | -0.0484 | 0.132 | 0.714 | 8.07E-05 | 39 |
| Systemic lupus erythematosus | rs17849502 | T | G | 0.0483 | 0.786 | 0.14 | 1.95E-08 | 0.184 | 0.254 | 0.469 | 6.53E-05 | 31.5 |
| Systemic lupus erythematosus | rs3021302 | C | T | 0.142 | 0.626 | 0.0724 | 5.25E-18 | -0.17 | 0.129 | 0.186 | 0.000155 | 74.8 |
| Systemic lupus erythematosus | rs4274624 | T | C | 0.744 | -0.378 | 0.0529 | 9.37E-13 | 0.122 | 0.116 | 0.29 | 0.000106 | 51 |
| Systemic lupus erythematosus | rs9494895 | T | C | 0.0398 | 0.666 | 0.119 | 2.31E-08 | -0.0213 | 0.365 | 0.954 | 6.46E-05 | 31.2 |
| Type 1 diabetes | rs10811662 | A | G | 0.23 | -0.141 | 0.0219 | 1.14E-10 | 0.0471 | 0.138 | 0.734 | 9.08E-05 | 41.6 |
| Type 1 diabetes | rs10876866 | A | G | 0.338 | 0.11 | 0.0199 | 2.69E-08 | 0.0269 | 0.104 | 0.796 | 6.74E-05 | 30.8 |
| Type 1 diabetes | rs115380430 | C | A | 0.0244 | 0.837 | 0.0886 | 3.45E-21 | 0.412 | 0.328 | 0.209 | 0.000195 | 89.3 |
| Type 1 diabetes | rs13204736 | A | G | 0.376 | 0.516 | 0.0218 | 3.30E-123 | 0.127 | 0.117 | 0.277 | 0.00122 | 560 |
| Type 1 diabetes | rs13204736 | A | G | 0.376 | 0.516 | 0.0218 | 3.30E-123 | -0.0824 | 0.211 | 0.696 | 0.00122 | 560 |
| Type 1 diabetes | rs17711850 | C | T | 0.335 | 0.124 | 0.0206 | 1.93E-09 | -0.072 | 0.112 | 0.521 | 7.86E-05 | 36 |
| Type 1 diabetes | rs189853531 | A | C | 0.00474 | 0.687 | 0.118 | 5.81E-09 | 0.572 | 0.635 | 0.368 | 7.41E-05 | 33.9 |
| Type 1 diabetes | rs192460891 | G | T | 0.14 | -0.286 | 0.0241 | 2.38E-32 | -0.177 | 0.126 | 0.157 | 0.000307 | 140 |
| Type 1 diabetes | rs2847278 | T | C | 0.837 | -0.155 | 0.0257 | 1.42E-09 | -0.299 | 0.137 | 0.0295 | 7.99E-05 | 36.6 |
| Type 1 diabetes | rs28891414 | T | C | 0.217 | -0.305 | 0.0227 | 5.24E-41 | -0.188 | 0.138 | 0.173 | 0.000394 | 180 |
| Type 1 diabetes | rs3184504 | C | T | 0.641 | -0.143 | 0.0189 | 3.76E-14 | 0.0054 | 0.0987 | 0.957 | 0.000126 | 57.5 |
| Type 1 diabetes | rs3842753 | G | T | 0.784 | 0.308 | 0.0222 | 8.31E-44 | 0.0599 | 0.12 | 0.619 | 0.000422 | 193 |
| Type 1 diabetes | rs6679677 | A | C | 0.114 | 0.426 | 0.0288 | 8.96E-50 | -0.0788 | 0.137 | 0.565 | 0.000479 | 219 |
| Type 1 diabetes | rs706779 | C | T | 0.434 | -0.0963 | 0.0174 | 3.23E-08 | 0.0261 | 0.1 | 0.794 | 6.69E-05 | 30.6 |
| Type 1 diabetes | rs7167984 | A | G | 0.221 | -0.126 | 0.0227 | 2.75E-08 | -0.208 | 0.136 | 0.125 | 6.75E-05 | 30.9 |
| Type 1 diabetes | rs72805613 | G | A | 0.361 | 0.0982 | 0.0179 | 4.04E-08 | 0.15 | 0.0992 | 0.129 | 6.58E-05 | 30.1 |
| Type 1 diabetes | rs7567242 | A | C | 0.167 | 0.142 | 0.0241 | 4.17E-09 | -0.0803 | 0.139 | 0.565 | 7.54E-05 | 34.5 |
| Type 1 diabetes | rs7903146 | T | C | 0.213 | 0.167 | 0.0217 | 1.48E-14 | 0.0614 | 0.121 | 0.613 | 0.000129 | 59.1 |
| Type 1 diabetes | rs9273363 | A | C | 0.323 | 0.73 | 0.0195 | 1.00E-200 | 0.0976 | 0.114 | 0.394 | 0.00306 | 1400 |
| Asthma | rs1011082 | C | T | 0.546 | 0.0823 | 0.0071 | 5.33E-31 | 0.0218 | 0.0979 | 0.824 | 0.000299 | 134 |
| Asthma | rs10934957 | C | G | 0.572 | -0.0397 | 0.0072 | 3.32E-08 | -0.143 | 0.0991 | 0.148 | 6.76E-05 | 30.4 |
| Asthma | rs11178649 | T | G | 0.375 | -0.0391 | 0.0071 | 4.33E-08 | -0.108 | 0.101 | 0.284 | 6.75E-05 | 30.3 |
| Asthma | rs11539209 | A | T | 0.0619 | -0.0837 | 0.0148 | 1.72E-08 | -0.2 | 0.219 | 0.361 | 7.11E-05 | 32 |
| Asthma | rs117710327 | A | C | 0.0624 | -0.109 | 0.0144 | 3.48E-14 | -0.0608 | 0.165 | 0.713 | 0.000128 | 57.6 |
| Asthma | rs12122629 | C | A | 0.0427 | 0.138 | 0.02 | 5.57E-12 | 0.373 | 0.252 | 0.138 | 0.000106 | 47.7 |
| Asthma | rs12253380 | G | A | 0.196 | -0.12 | 0.0109 | 3.48E-28 | 0.0906 | 0.187 | 0.628 | 0.000271 | 122 |
| Asthma | rs12697352 | A | G | 0.309 | -0.0572 | 0.0075 | 2.80E-14 | 0.0639 | 0.0996 | 0.521 | 0.000129 | 58.2 |
| Asthma | rs12700215 | A | G | 0.331 | -0.0477 | 0.0074 | 9.60E-11 | -0.0758 | 0.0982 | 0.44 | 9.24E-05 | 41.6 |
| Asthma | rs12935657 | A | G | 0.195 | -0.0722 | 0.0088 | 3.15E-16 | -0.0299 | 0.119 | 0.802 | 0.00015 | 67.3 |
| Asthma | rs1295685 | G | A | 0.754 | -0.082 | 0.008 | 2.29E-24 | -0.0546 | 0.102 | 0.59 | 0.000234 | 105 |
| Asthma | rs13098877 | T | C | 0.485 | -0.0499 | 0.007 | 9.53E-13 | -0.0224 | 0.0976 | 0.818 | 0.000113 | 50.8 |
| Asthma | rs13188700 | C | T | 0.625 | 0.0459 | 0.0071 | 1.20E-10 | -0.155 | 0.102 | 0.13 | 9.30E-05 | 41.8 |
| Asthma | rs13263709 | C | T | 0.589 | -0.053 | 0.0072 | 1.85E-13 | -0.0805 | 0.105 | 0.444 | 0.000121 | 54.2 |
| Asthma | rs1504215 | A | G | 0.325 | -0.0877 | 0.0087 | 5.33E-24 | -0.105 | 0.114 | 0.357 | 0.000226 | 102 |
| Asthma | rs1702877 | T | C | 0.295 | 0.0663 | 0.0076 | 2.91E-18 | 0.0273 | 0.104 | 0.792 | 0.000169 | 76.1 |
| Asthma | rs1741708 | T | G | 0.328 | -0.0434 | 0.0078 | 3.01E-08 | -0.164 | 0.122 | 0.178 | 6.89E-05 | 31 |
| Asthma | rs174568 | T | C | 0.369 | -0.0437 | 0.0071 | 9.67E-10 | 0.0101 | 0.0988 | 0.918 | 8.43E-05 | 37.9 |
| Asthma | rs1775550 | A | G | 0.798 | -0.114 | 0.0085 | 7.95E-41 | 0.145 | 0.108 | 0.179 | 0.000401 | 181 |
| Asthma | rs1904522 | A | G | 0.403 | 0.0546 | 0.0071 | 1.57E-14 | -0.0935 | 0.0975 | 0.338 | 0.000132 | 59.1 |
| Asthma | rs2070901 | T | G | 0.334 | 0.0469 | 0.0075 | 3.72E-10 | 0.0434 | 0.108 | 0.688 | 8.70E-05 | 39.1 |
| Asthma | rs2248352 | G | A | 0.359 | 0.0584 | 0.0073 | 1.60E-15 | 0.276 | 0.11 | 0.0119 | 0.000142 | 64 |
| Asthma | rs2279802 | A | G | 0.22 | 0.0537 | 0.0097 | 3.58E-08 | -0.0497 | 0.158 | 0.753 | 6.82E-05 | 30.6 |
| Asthma | rs2834787 | G | A | 0.112 | 0.0779 | 0.011 | 1.49E-12 | 0.248 | 0.138 | 0.0716 | 0.000112 | 50.2 |
| Asthma | rs3024556 | A | G | 0.386 | 0.0764 | 0.0074 | 1.11E-24 | 0.189 | 0.109 | 0.0827 | 0.000237 | 107 |
| Asthma | rs34276574 | T | C | 0.313 | -0.0489 | 0.0088 | 2.66E-08 | 0.169 | 0.11 | 0.124 | 6.87E-05 | 30.9 |
| Asthma | rs34290285 | A | G | 0.221 | -0.108 | 0.0085 | 5.71E-37 | -0.0826 | 0.116 | 0.478 | 0.000359 | 161 |
| Asthma | rs346835 | T | C | 0.316 | -0.0537 | 0.0074 | 5.32E-13 | 0.0655 | 0.102 | 0.523 | 0.000117 | 52.7 |
| Asthma | rs34809330 | T | G | 0.377 | 0.0533 | 0.0086 | 5.63E-10 | 0.0343 | 0.131 | 0.793 | 8.54E-05 | 38.4 |
| Asthma | rs35570272 | T | G | 0.421 | 0.0542 | 0.0071 | 1.92E-14 | -0.081 | 0.104 | 0.437 | 0.00013 | 58.3 |
| Asthma | rs3732192 | C | T | 0.0927 | -0.073 | 0.0125 | 5.71E-09 | -0.504 | 0.218 | 0.0206 | 7.59E-05 | 34.1 |
| Asthma | rs3771175 | A | T | 0.148 | -0.124 | 0.0097 | 2.48E-37 | 0.0282 | 0.132 | 0.831 | 0.000363 | 163 |
| Asthma | rs3920174 | G | A | 0.464 | 0.0388 | 0.0071 | 4.60E-08 | 0.0744 | 0.104 | 0.476 | 6.64E-05 | 29.9 |
| Asthma | rs4776887 | A | G | 0.532 | 0.0388 | 0.0069 | 2.13E-08 | -0.26 | 0.0977 | 0.00768 | 7.03E-05 | 31.6 |
| Asthma | rs55730955 | A | T | 0.18 | -0.081 | 0.0108 | 6.80E-14 | 0.0712 | 0.189 | 0.707 | 0.000125 | 56.2 |
| Asthma | rs5743618 | A | C | 0.428 | -0.0664 | 0.01 | 2.66E-11 | 0.0264 | 0.142 | 0.852 | 9.81E-05 | 44.1 |
| Asthma | rs61907683 | C | T | 0.212 | -0.0582 | 0.0084 | 4.88E-12 | -0.028 | 0.113 | 0.804 | 0.000107 | 48 |
| Asthma | rs6687430 | A | G | 0.599 | 0.0431 | 0.0072 | 2.06E-09 | 0.0777 | 0.0978 | 0.427 | 7.97E-05 | 35.8 |
| Asthma | rs703816 | C | T | 0.376 | 0.0578 | 0.0073 | 2.45E-15 | -0.0349 | 0.0985 | 0.723 | 0.000139 | 62.7 |
| Asthma | rs7099257 | A | T | 0.532 | -0.0458 | 0.0072 | 1.71E-10 | 0.0992 | 0.1 | 0.324 | 9.00E-05 | 40.5 |
| Asthma | rs7258329 | A | C | 0.308 | 0.0492 | 0.0078 | 2.25E-10 | -0.106 | 0.115 | 0.356 | 8.85E-05 | 39.8 |
| Asthma | rs72743461 | A | C | 0.187 | 0.107 | 0.009 | 5.42E-33 | -0.0624 | 0.111 | 0.573 | 0.000317 | 142 |
| Asthma | rs72837816 | C | A | 0.103 | 0.103 | 0.0132 | 6.12E-15 | 0.138 | 0.157 | 0.379 | 0.000135 | 60.9 |
| Asthma | rs72941069 | C | T | 0.126 | -0.0662 | 0.0106 | 3.90E-10 | 0.112 | 0.154 | 0.469 | 8.68E-05 | 39 |
| Asthma | rs7521458 | C | T | 0.397 | 0.0407 | 0.0071 | 1.00E-08 | -0.0993 | 0.106 | 0.346 | 7.31E-05 | 32.9 |
| Asthma | rs76181804 | G | A | 0.108 | -0.0808 | 0.0132 | 8.74E-10 | 0.0076 | 0.187 | 0.968 | 8.34E-05 | 37.5 |
| Asthma | rs77450473 | A | G | 0.104 | 0.0776 | 0.0112 | 5.20E-12 | -0.0152 | 0.121 | 0.9 | 0.000107 | 48 |
| Asthma | rs8129030 | A | T | 0.594 | 0.0512 | 0.0071 | 5.86E-13 | -0.0636 | 0.102 | 0.533 | 0.000116 | 52 |
| Asthma | rs9272245 | G | C | 0.571 | -0.088 | 0.0072 | 1.73E-34 | -0.0664 | 0.104 | 0.523 | 0.000332 | 149 |
| Asthma | rs9405074 | G | A | 0.0607 | -0.0931 | 0.0155 | 2.17E-09 | -0.242 | 0.296 | 0.414 | 8.03E-05 | 36.1 |
| Asthma | rs9517638 | C | T | 0.624 | 0.0492 | 0.0073 | 2.10E-11 | 0.0728 | 0.102 | 0.474 | 0.000101 | 45.4 |
| Asthma | rs9603616 | T | C | 0.322 | -0.0408 | 0.0074 | 3.76E-08 | -0.0771 | 0.103 | 0.453 | 6.76E-05 | 30.4 |
| Asthma | rs992969 | G | A | 0.812 | -0.1 | 0.009 | 1.60E-28 | -0.0588 | 0.114 | 0.606 | 0.000275 | 124 |
| Graves' disease | rs10821944 | T | G | 0.703 | -0.157 | 0.0232 | 1.43E-11 | 0.226 | 0.108 | 0.0366 | 9.93E-05 | 45.6 |
| Graves' disease | rs12612769 | C | A | 0.218 | 0.145 | 0.0252 | 8.78E-09 | -0.16 | 0.121 | 0.187 | 7.21E-05 | 33.1 |
| Graves' disease | rs13136820 | T | C | 0.7 | -0.136 | 0.0244 | 2.37E-08 | -0.0671 | 0.11 | 0.543 | 6.78E-05 | 31.1 |
| Graves' disease | rs13210649 | G | T | 0.442 | 0.135 | 0.0219 | 6.87E-10 | 0.0133 | 0.0993 | 0.894 | 8.33E-05 | 38.2 |
| Graves' disease | rs1569723 | A | C | 0.712 | 0.164 | 0.0232 | 1.62E-12 | 0.184 | 0.11 | 0.0919 | 0.000109 | 50 |
| Graves' disease | rs17689159 | C | T | 0.305 | 0.162 | 0.024 | 1.55E-11 | 0.0056 | 0.105 | 0.958 | 9.95E-05 | 45.6 |
| Graves' disease | rs1977710 | G | A | 0.444 | 0.146 | 0.0223 | 5.19E-11 | 0.13 | 0.0982 | 0.186 | 9.36E-05 | 42.9 |
| Graves' disease | rs231779 | T | C | 0.468 | 0.235 | 0.0222 | 3.61E-26 | 0.18 | 0.0971 | 0.064 | 0.000245 | 112 |
| Graves' disease | rs2466028 | C | T | 0.361 | -0.145 | 0.0235 | 6.28E-10 | -0.0795 | 0.103 | 0.442 | 8.30E-05 | 38.1 |
| Graves' disease | rs28414437 | C | A | 0.424 | 0.287 | 0.0228 | 1.98E-36 | -0.0927 | 0.106 | 0.384 | 0.000346 | 159 |
| Graves' disease | rs34544259 | G | A | 0.405 | 0.141 | 0.0228 | 6.75E-10 | -0.0703 | 0.101 | 0.488 | 8.29E-05 | 38 |
| Graves' disease | rs4409785 | C | T | 0.144 | 0.203 | 0.035 | 6.81E-09 | -0.0415 | 0.13 | 0.749 | 7.33E-05 | 33.6 |
| Graves' disease | rs5751536 | A | G | 0.451 | -0.21 | 0.038 | 3.37E-08 | -0.216 | 0.104 | 0.0365 | 6.63E-05 | 30.4 |
| Graves' disease | rs61226717 | T | G | 0.109 | 0.236 | 0.0357 | 3.60E-11 | 0.18 | 0.196 | 0.356 | 9.57E-05 | 43.9 |
| Graves' disease | rs61850863 | A | G | 0.145 | 0.203 | 0.0339 | 2.18E-09 | -0.216 | 0.122 | 0.0766 | 7.80E-05 | 35.8 |
| Graves' disease | rs6679677 | A | C | 0.112 | 0.377 | 0.0536 | 2.14E-12 | -0.0788 | 0.137 | 0.565 | 0.000108 | 49.4 |
| Graves' disease | rs6780858 | G | A | 0.468 | -0.163 | 0.0225 | 3.85E-13 | 0.0366 | 0.0984 | 0.71 | 0.000115 | 52.7 |
| Graves' disease | rs6936707 | T | C | 0.0351 | -0.463 | 0.0479 | 4.61E-22 | 0.844 | 0.96 | 0.379 | 0.000203 | 93.3 |
| Graves' disease | rs7741597 | C | T | 0.144 | -0.222 | 0.029 | 2.27E-14 | -0.103 | 0.153 | 0.501 | 0.000127 | 58.3 |
| Graves' disease | rs7746061 | A | G | 0.0557 | -0.384 | 0.0516 | 1.02E-13 | 0.0118 | 0.273 | 0.966 | 0.00012 | 55.3 |
| Graves' disease | rs7754251 | C | G | 0.49 | 0.131 | 0.0227 | 7.16E-09 | -0.157 | 0.0976 | 0.108 | 7.28E-05 | 33.4 |
| Graves' disease | rs860262 | A | C | 0.418 | -0.137 | 0.0245 | 2.11E-08 | -0.268 | 0.0974 | 0.00602 | 6.85E-05 | 31.4 |
| Graves' disease | rs9260041 | T | C | 0.29 | -0.342 | 0.0239 | 1.77E-46 | 0.18 | 0.127 | 0.156 | 0.000446 | 205 |
| Graves' disease | rs9273410 | A | C | 0.429 | 0.307 | 0.024 | 1.52E-37 | -0.0636 | 0.104 | 0.541 | 0.000357 | 164 |
| Graves' disease | rs9357156 | C | A | 0.231 | 0.44 | 0.0251 | 1.09E-68 | -0.0352 | 0.171 | 0.837 | 0.000668 | 307 |

Supplementary Table S2: Characteristics of genetic instruments and harmonized SNPs for the seven systemic autoimmune diseases.
