# Supplementary material for: Association between Guillain–Barré syndrome and 7 autoimmune diseases: a mendelian randomization study
Source: BMC Neurol. 2026 May 9;26:425. doi: 10.1186/s12883-026-04957-8 (PMC13326399; doi:10.1186/s12883-026-04957-8)
Supplement: Supplementary file 3 — Supplementary Material 3. Table S3. [file 12883_2026_4957_MOESM3_ESM.docx]

| Exposure | Outcome | nSNP | Reverse_P_Value |
| --- | --- | --- | --- |
| GBS | Psoriasis vulgaris | 6 | 0.793875553 |
| GBS | Rheumatoid arthritis | 6 | 0.888102039 |
| GBS | Sarcoidosis | 6 | 0.401545361 |
| GBS | Systemic lupus erythematosus | 6 | 0.150632589 |
| GBS | Type 1 diabetes | 6 | 0.511974385 |
| GBS | Asthma | 6 | 0.644873996 |
| GBS | Graves' disease | 6 | 0.216188151 |

Supplementary Table S3: Results of reverse Mendelian randomization analysis evaluating the potential effect of Guillain-Barré syndrome on autoimmune diseases.
