# Supplementary material for: Association between Guillain–Barré syndrome and 7 autoimmune diseases: a mendelian randomization study
Source: BMC Neurol. 2026 May 9;26:425. doi: 10.1186/s12883-026-04957-8 (PMC13326399; doi:10.1186/s12883-026-04957-8)
Supplement: Supplementary file 4 — Supplementary Material 4. Table S4. [file 12883_2026_4957_MOESM4_ESM.docx]

| **Exposure** | **Mediator_ID** | **Total_Beta** | **Indirect_Beta** | **Indirect_SE** | **Indirect_P** | **Proportion_Mediated** | **Indirect_FDR** | **Significance** |
| --- | --- | --- | --- | --- | --- | --- | --- | --- |
| Type 1 diabetes | ebi-a-GCST90002087 | 0.23 | 0.0773 | 0.0367 | 0.0351 | 0.336 | 1 | Non-significant |
| Psoriasis vulgaris | ebi-a-GCST90002010 | -0.244 | -0.0374 | 0.023 | 0.104 | 0.153 | 1 | Non-significant |
| Psoriasis vulgaris | ebi-a-GCST90002104 | -0.244 | -0.0321 | 0.0211 | 0.128 | 0.132 | 1 | Non-significant |
| Psoriasis vulgaris | ebi-a-GCST90002106 | -0.244 | -0.0411 | 0.0275 | 0.136 | 0.168 | 1 | Non-significant |
| Type 1 diabetes | ebi-a-GCST90001752 | 0.23 | 0.046 | 0.0311 | 0.139 | 0.2 | 1 | Non-significant |
| Psoriasis vulgaris | ebi-a-GCST90001991 | -0.244 | -0.0287 | 0.0206 | 0.164 | 0.118 | 1 | Non-significant |
| Psoriasis vulgaris | ebi-a-GCST90001988 | -0.244 | -0.0275 | 0.0201 | 0.171 | 0.113 | 1 | Non-significant |
| Psoriasis vulgaris | ebi-a-GCST90002014 | -0.244 | -0.0466 | 0.0342 | 0.172 | 0.191 | 1 | Non-significant |
| Psoriasis vulgaris | ebi-a-GCST90001714 | -0.244 | -0.433 | 0.322 | 0.179 | 1.78 | 1 | Non-significant |
| Type 1 diabetes | ebi-a-GCST90001757 | 0.23 | 0.0407 | 0.0303 | 0.179 | 0.177 | 1 | Non-significant |
| Psoriasis vulgaris | ebi-a-GCST90001471 | -0.244 | 0.0378 | 0.0287 | 0.187 | -0.155 | 1 | Non-significant |
| Type 1 diabetes | ebi-a-GCST90002062 | 0.23 | 0.0585 | 0.0446 | 0.189 | 0.255 | 1 | Non-significant |
| Psoriasis vulgaris | ebi-a-GCST90002109 | -0.244 | -0.0468 | 0.0357 | 0.19 | 0.192 | 1 | Non-significant |
| Psoriasis vulgaris | ebi-a-GCST90002108 | -0.244 | -0.0426 | 0.0326 | 0.192 | 0.175 | 1 | Non-significant |
| Type 1 diabetes | ebi-a-GCST90001761 | 0.23 | 0.0382 | 0.0299 | 0.201 | 0.166 | 1 | Non-significant |
| Type 1 diabetes | ebi-a-GCST90001412 | 0.23 | 0.174 | 0.137 | 0.206 | 0.756 | 1 | Non-significant |
| Psoriasis vulgaris | ebi-a-GCST90001451 | -0.244 | -0.0497 | 0.0395 | 0.208 | 0.204 | 1 | Non-significant |
| Psoriasis vulgaris | ebi-a-GCST90002112 | -0.244 | -0.0364 | 0.0305 | 0.232 | 0.149 | 1 | Non-significant |
| Psoriasis vulgaris | ebi-a-GCST90001907 | -0.244 | 0.0318 | 0.0267 | 0.233 | -0.13 | 1 | Non-significant |
| Psoriasis vulgaris | ebi-a-GCST90002105 | -0.244 | -0.0313 | 0.0265 | 0.237 | 0.128 | 1 | Non-significant |
| Type 1 diabetes | ebi-a-GCST90001799 | 0.23 | -0.0328 | 0.0283 | 0.246 | -0.143 | 1 | Non-significant |
| Type 1 diabetes | ebi-a-GCST90001759 | 0.23 | 0.0367 | 0.0318 | 0.248 | 0.16 | 1 | Non-significant |
| Type 1 diabetes | ebi-a-GCST90001863 | 0.23 | -0.0201 | 0.0174 | 0.249 | -0.0874 | 1 | Non-significant |
| Type 1 diabetes | ebi-a-GCST90001418 | 0.23 | 0.138 | 0.121 | 0.255 | 0.599 | 1 | Non-significant |
| Psoriasis vulgaris | ebi-a-GCST90002103 | -0.244 | 0.138 | 0.123 | 0.263 | -0.565 | 1 | Non-significant |
| Psoriasis vulgaris | ebi-a-GCST90001943 | -0.244 | -0.0403 | 0.0363 | 0.267 | 0.165 | 1 | Non-significant |
| Type 1 diabetes | ebi-a-GCST90001804 | 0.23 | -0.00982 | 0.00886 | 0.267 | -0.0427 | 1 | Non-significant |
| Psoriasis vulgaris | ebi-a-GCST90001530 | -0.244 | -0.0184 | 0.0167 | 0.272 | 0.0754 | 1 | Non-significant |
| Psoriasis vulgaris | ebi-a-GCST90002116 | -0.244 | 0.0112 | 0.0103 | 0.275 | -0.0461 | 1 | Non-significant |
| Type 1 diabetes | ebi-a-GCST90001523 | 0.23 | 0.043 | 0.0396 | 0.277 | 0.187 | 1 | Non-significant |
| Type 1 diabetes | ebi-a-GCST90001996 | 0.23 | -0.0129 | 0.012 | 0.282 | -0.0562 | 1 | Non-significant |
| Type 1 diabetes | ebi-a-GCST90002065 | 0.23 | 0.0416 | 0.0389 | 0.285 | 0.181 | 1 | Non-significant |
| Type 1 diabetes | ebi-a-GCST90001840 | 0.23 | -0.0304 | 0.0285 | 0.286 | -0.132 | 1 | Non-significant |
| Type 1 diabetes | ebi-a-GCST90002056 | 0.23 | -0.0201 | 0.0191 | 0.292 | -0.0876 | 1 | Non-significant |
| Type 1 diabetes | ebi-a-GCST90001747 | 0.23 | 0.0358 | 0.034 | 0.293 | 0.156 | 1 | Non-significant |
| Type 1 diabetes | ebi-a-GCST90001834 | 0.23 | 0.019 | 0.0181 | 0.294 | 0.0828 | 1 | Non-significant |
| Type 1 diabetes | ebi-a-GCST90002116 | 0.23 | 0.0232 | 0.0221 | 0.294 | 0.101 | 1 | Non-significant |
| Psoriasis vulgaris | ebi-a-GCST90001857 | -0.244 | 0.0167 | 0.016 | 0.296 | -0.0686 | 1 | Non-significant |
| Type 1 diabetes | ebi-a-GCST90001885 | 0.23 | 0.038 | 0.0364 | 0.296 | 0.165 | 1 | Non-significant |
| Psoriasis vulgaris | ebi-a-GCST90001476 | -0.244 | -0.0284 | 0.0274 | 0.298 | 0.117 | 1 | Non-significant |
| Psoriasis vulgaris | ebi-a-GCST90001675 | -0.244 | 0.0908 | 0.0874 | 0.299 | -0.372 | 1 | Non-significant |
| Psoriasis vulgaris | ebi-a-GCST90001854 | -0.244 | 0.0131 | 0.0127 | 0.303 | -0.0537 | 1 | Non-significant |
| Type 1 diabetes | ebi-a-GCST90002008 | 0.23 | 0.0286 | 0.0278 | 0.303 | 0.125 | 1 | Non-significant |
| Type 1 diabetes | ebi-a-GCST90002057 | 0.23 | -0.0225 | 0.0219 | 0.304 | -0.098 | 1 | Non-significant |
| Type 1 diabetes | ebi-a-GCST90001525 | 0.23 | -0.0296 | 0.029 | 0.307 | -0.129 | 1 | Non-significant |
| Type 1 diabetes | ebi-a-GCST90002067 | 0.23 | 0.0281 | 0.0277 | 0.31 | 0.122 | 1 | Non-significant |
| Psoriasis vulgaris | ebi-a-GCST90001467 | -0.244 | 0.0508 | 0.0501 | 0.311 | -0.208 | 1 | Non-significant |
| Type 1 diabetes | ebi-a-GCST90001850 | 0.23 | -0.0272 | 0.027 | 0.313 | -0.119 | 1 | Non-significant |
| Type 1 diabetes | ebi-a-GCST90001904 | 0.23 | 0.0182 | 0.0181 | 0.316 | 0.0791 | 1 | Non-significant |
| Type 1 diabetes | ebi-a-GCST90001832 | 0.23 | 0.0253 | 0.0253 | 0.317 | 0.11 | 1 | Non-significant |
| Psoriasis vulgaris | ebi-a-GCST90001650 | -0.244 | 0.0167 | 0.0168 | 0.319 | -0.0685 | 1 | Non-significant |
| Type 1 diabetes | ebi-a-GCST90001582 | 0.23 | -0.0261 | 0.0262 | 0.319 | -0.113 | 1 | Non-significant |
| Type 1 diabetes | ebi-a-GCST90001906 | 0.23 | -0.0466 | 0.0468 | 0.319 | -0.203 | 1 | Non-significant |
| Type 1 diabetes | ebi-a-GCST90001868 | 0.23 | -0.093 | 0.0938 | 0.322 | -0.405 | 1 | Non-significant |
| Psoriasis vulgaris | ebi-a-GCST90001789 | -0.244 | -0.0225 | 0.0228 | 0.324 | 0.0923 | 1 | Non-significant |
| Type 1 diabetes | ebi-a-GCST90001399 | 0.23 | -0.0188 | 0.0191 | 0.324 | -0.0818 | 1 | Non-significant |
| Type 1 diabetes | ebi-a-GCST90001678 | 0.23 | -0.0394 | 0.0401 | 0.326 | -0.172 | 1 | Non-significant |
| Type 1 diabetes | ebi-a-GCST90001980 | 0.23 | -0.0108 | 0.011 | 0.327 | -0.0468 | 1 | Non-significant |
| Psoriasis vulgaris | ebi-a-GCST90001932 | -0.244 | -0.0246 | 0.0251 | 0.328 | 0.101 | 1 | Non-significant |
| Psoriasis vulgaris | ebi-a-GCST90002111 | -0.244 | -0.0304 | 0.0311 | 0.328 | 0.125 | 1 | Non-significant |
| Type 1 diabetes | ebi-a-GCST90001974 | 0.23 | 0.0683 | 0.0698 | 0.328 | 0.297 | 1 | Non-significant |
| Psoriasis vulgaris | ebi-a-GCST90001450 | -0.244 | -0.0388 | 0.0397 | 0.329 | 0.159 | 1 | Non-significant |
| Psoriasis vulgaris | ebi-a-GCST90001701 | -0.244 | -0.0219 | 0.0226 | 0.332 | 0.0899 | 1 | Non-significant |
| Type 1 diabetes | ebi-a-GCST90001407 | 0.23 | 0.131 | 0.136 | 0.334 | 0.57 | 1 | Non-significant |
| Psoriasis vulgaris | ebi-a-GCST90001649 | -0.244 | 0.0157 | 0.0164 | 0.339 | -0.0642 | 1 | Non-significant |
| Psoriasis vulgaris | ebi-a-GCST90001840 | -0.244 | -0.027 | 0.0283 | 0.34 | 0.111 | 1 | Non-significant |
| Type 1 diabetes | ebi-a-GCST90001704 | 0.23 | -0.00524 | 0.00553 | 0.343 | -0.0228 | 1 | Non-significant |
| Psoriasis vulgaris | ebi-a-GCST90001808 | -0.244 | -0.00894 | 0.00944 | 0.343 | 0.0367 | 1 | Non-significant |
| Type 1 diabetes | ebi-a-GCST90002104 | 0.23 | 0.0195 | 0.0207 | 0.347 | 0.0848 | 1 | Non-significant |
| Type 1 diabetes | ebi-a-GCST90001762 | 0.23 | 0.0282 | 0.0299 | 0.347 | 0.123 | 1 | Non-significant |
| Psoriasis vulgaris | ebi-a-GCST90001399 | -0.244 | -0.0194 | 0.0207 | 0.349 | 0.0796 | 1 | Non-significant |
| Type 1 diabetes | ebi-a-GCST90001855 | 0.23 | -0.00875 | 0.00933 | 0.349 | -0.0381 | 1 | Non-significant |
| Psoriasis vulgaris | ebi-a-GCST90001429 | -0.244 | 0.22 | 0.236 | 0.35 | -0.904 | 1 | Non-significant |
| Psoriasis vulgaris | ebi-a-GCST90001391 | -0.244 | -0.051 | 0.0546 | 0.35 | 0.209 | 1 | Non-significant |
| Psoriasis vulgaris | ebi-a-GCST90001798 | -0.244 | -0.00938 | 0.0101 | 0.356 | 0.0385 | 1 | Non-significant |
| Psoriasis vulgaris | ebi-a-GCST90001853 | -0.244 | 0.0144 | 0.0156 | 0.357 | -0.0589 | 1 | Non-significant |
| Type 1 diabetes | ebi-a-GCST90002079 | 0.23 | 0.0583 | 0.0635 | 0.359 | 0.254 | 1 | Non-significant |
| Type 1 diabetes | ebi-a-GCST90001688 | 0.23 | -0.00059 | 0.000648 | 0.36 | -0.00258 | 1 | Non-significant |
| Psoriasis vulgaris | ebi-a-GCST90002001 | -0.244 | -0.0235 | 0.0258 | 0.361 | 0.0966 | 1 | Non-significant |
| Psoriasis vulgaris | ebi-a-GCST90001984 | -0.244 | -0.0166 | 0.0182 | 0.362 | 0.0682 | 1 | Non-significant |
| Type 1 diabetes | ebi-a-GCST90001391 | 0.23 | -0.042 | 0.0461 | 0.363 | -0.183 | 1 | Non-significant |
| Psoriasis vulgaris | ebi-a-GCST90001908 | -0.244 | 0.0299 | 0.033 | 0.365 | -0.123 | 1 | Non-significant |
| Psoriasis vulgaris | ebi-a-GCST90001547 | -0.244 | -0.0112 | 0.0125 | 0.371 | 0.0461 | 1 | Non-significant |
| Psoriasis vulgaris | ebi-a-GCST90001475 | -0.244 | -0.0192 | 0.0217 | 0.375 | 0.0789 | 1 | Non-significant |
| Psoriasis vulgaris | ebi-a-GCST90001648 | -0.244 | 0.0139 | 0.0157 | 0.376 | -0.057 | 1 | Non-significant |
| Psoriasis vulgaris | ebi-a-GCST90001927 | -0.244 | 0.0339 | 0.0383 | 0.376 | -0.139 | 1 | Non-significant |
| Type 1 diabetes | ebi-a-GCST90001866 | 0.23 | -0.0154 | 0.0175 | 0.377 | -0.0672 | 1 | Non-significant |
| Type 1 diabetes | ebi-a-GCST90002021 | 0.23 | 0.0217 | 0.0246 | 0.378 | 0.0944 | 1 | Non-significant |
| Psoriasis vulgaris | ebi-a-GCST90001582 | -0.244 | -0.0338 | 0.0384 | 0.379 | 0.139 | 1 | Non-significant |
| Type 1 diabetes | ebi-a-GCST90001755 | 0.23 | 0.0211 | 0.0241 | 0.38 | 0.092 | 1 | Non-significant |
| Psoriasis vulgaris | ebi-a-GCST90001470 | -0.244 | 0.0237 | 0.027 | 0.38 | -0.0971 | 1 | Non-significant |
| Psoriasis vulgaris | ebi-a-GCST90001417 | -0.244 | -0.0219 | 0.025 | 0.381 | 0.0897 | 1 | Non-significant |
| Type 1 diabetes | ebi-a-GCST90002040 | 0.23 | -0.0192 | 0.0219 | 0.381 | -0.0836 | 1 | Non-significant |
| Type 1 diabetes | ebi-a-GCST90001514 | 0.23 | 0.00903 | 0.0103 | 0.382 | 0.0393 | 1 | Non-significant |
| Type 1 diabetes | ebi-a-GCST90001708 | 0.23 | -0.00498 | 0.00572 | 0.384 | -0.0217 | 1 | Non-significant |
| Psoriasis vulgaris | ebi-a-GCST90001924 | -0.244 | -0.0265 | 0.0305 | 0.384 | 0.109 | 1 | Non-significant |
| Type 1 diabetes | ebi-a-GCST90001843 | 0.23 | -0.0143 | 0.0165 | 0.386 | -0.0621 | 1 | Non-significant |
| Type 1 diabetes | ebi-a-GCST90001675 | 0.23 | -0.0413 | 0.0477 | 0.386 | -0.18 | 1 | Non-significant |
| Type 1 diabetes | ebi-a-GCST90001753 | 0.23 | 0.0464 | 0.0537 | 0.387 | 0.202 | 1 | Non-significant |
| Type 1 diabetes | ebi-a-GCST90001583 | 0.23 | -0.0315 | 0.0366 | 0.389 | -0.137 | 1 | Non-significant |
| Psoriasis vulgaris | ebi-a-GCST90002000 | -0.244 | -0.0446 | 0.0523 | 0.394 | 0.183 | 1 | Non-significant |
| Type 1 diabetes | ebi-a-GCST90001450 | 0.23 | 0.0288 | 0.0338 | 0.394 | 0.125 | 1 | Non-significant |
| Type 1 diabetes | ebi-a-GCST90002076 | 0.23 | 0.0199 | 0.0235 | 0.397 | 0.0866 | 1 | Non-significant |
| Psoriasis vulgaris | ebi-a-GCST90001583 | -0.244 | -0.0524 | 0.0621 | 0.399 | 0.215 | 1 | Non-significant |
| Psoriasis vulgaris | ebi-a-GCST90002076 | -0.244 | 0.0238 | 0.0284 | 0.401 | -0.0978 | 1 | Non-significant |
| Psoriasis vulgaris | ebi-a-GCST90001543 | -0.244 | 0.0274 | 0.0327 | 0.402 | -0.112 | 1 | Non-significant |
| Type 1 diabetes | ebi-a-GCST90001542 | 0.23 | -0.0204 | 0.0244 | 0.404 | -0.0886 | 1 | Non-significant |
| Psoriasis vulgaris | ebi-a-GCST90001855 | -0.244 | 0.00975 | 0.0117 | 0.404 | -0.04 | 1 | Non-significant |
| Psoriasis vulgaris | ebi-a-GCST90001531 | -0.244 | 0.00586 | 0.00704 | 0.405 | -0.024 | 1 | Non-significant |
| Psoriasis vulgaris | ebi-a-GCST90002005 | -0.244 | 0.0117 | 0.0141 | 0.407 | -0.0479 | 1 | Non-significant |
| Type 1 diabetes | ebi-a-GCST90001993 | 0.23 | 0.04 | 0.0484 | 0.409 | 0.174 | 1 | Non-significant |
| Type 1 diabetes | ebi-a-GCST90002039 | 0.23 | 0.00669 | 0.00816 | 0.412 | 0.0291 | 1 | Non-significant |
| Psoriasis vulgaris | ebi-a-GCST90002087 | -0.244 | -0.0368 | 0.0449 | 0.414 | 0.151 | 1 | Non-significant |
| Psoriasis vulgaris | ebi-a-GCST90002007 | -0.244 | -0.0169 | 0.0207 | 0.414 | 0.0694 | 1 | Non-significant |
| Type 1 diabetes | ebi-a-GCST90001926 | 0.23 | -0.0057 | 0.00699 | 0.415 | -0.0248 | 1 | Non-significant |
| Psoriasis vulgaris | ebi-a-GCST90001532 | -0.244 | 0.00562 | 0.0069 | 0.416 | -0.023 | 1 | Non-significant |
| Type 1 diabetes | ebi-a-GCST90001932 | 0.23 | 0.0198 | 0.0243 | 0.416 | 0.0862 | 1 | Non-significant |
| Type 1 diabetes | ebi-a-GCST90001721 | 0.23 | 0.0111 | 0.0136 | 0.416 | 0.0482 | 1 | Non-significant |
| Psoriasis vulgaris | ebi-a-GCST90001760 | -0.244 | -0.019 | 0.0234 | 0.417 | 0.0778 | 1 | Non-significant |
| Psoriasis vulgaris | ebi-a-GCST90001804 | -0.244 | -0.00733 | 0.00904 | 0.417 | 0.0301 | 1 | Non-significant |
| Type 1 diabetes | ebi-a-GCST90001827 | 0.23 | -0.0111 | 0.0137 | 0.418 | -0.0482 | 1 | Non-significant |
| Type 1 diabetes | ebi-a-GCST90001527 | 0.23 | -0.0222 | 0.0274 | 0.419 | -0.0964 | 1 | Non-significant |
| Psoriasis vulgaris | ebi-a-GCST90002008 | -0.244 | -0.0261 | 0.0324 | 0.421 | 0.107 | 1 | Non-significant |
| Psoriasis vulgaris | ebi-a-GCST90001934 | -0.244 | 0.0209 | 0.0261 | 0.423 | -0.0857 | 1 | Non-significant |
| Type 1 diabetes | ebi-a-GCST90001404 | 0.23 | 0.0494 | 0.0616 | 0.423 | 0.215 | 1 | Non-significant |
| Type 1 diabetes | ebi-a-GCST90001928 | 0.23 | -0.0196 | 0.0246 | 0.425 | -0.0852 | 1 | Non-significant |
| Type 1 diabetes | ebi-a-GCST90001822 | 0.23 | -0.0152 | 0.0192 | 0.428 | -0.0663 | 1 | Non-significant |
| Psoriasis vulgaris | ebi-a-GCST90001500 | -0.244 | 0.00845 | 0.0107 | 0.428 | -0.0347 | 1 | Non-significant |
| Psoriasis vulgaris | ebi-a-GCST90001802 | -0.244 | -0.00847 | 0.0107 | 0.429 | 0.0347 | 1 | Non-significant |
| Type 1 diabetes | ebi-a-GCST90001426 | 0.23 | -0.0261 | 0.033 | 0.429 | -0.113 | 1 | Non-significant |
| Psoriasis vulgaris | ebi-a-GCST90001491 | -0.244 | -0.00465 | 0.00589 | 0.43 | 0.0191 | 1 | Non-significant |
| Type 1 diabetes | ebi-a-GCST90001859 | 0.23 | -0.0108 | 0.0137 | 0.43 | -0.047 | 1 | Non-significant |
| Type 1 diabetes | ebi-a-GCST90002106 | 0.23 | 0.0139 | 0.0177 | 0.434 | 0.0603 | 1 | Non-significant |
| Type 1 diabetes | ebi-a-GCST90001709 | 0.23 | -0.00423 | 0.00541 | 0.434 | -0.0184 | 1 | Non-significant |
| Type 1 diabetes | ebi-a-GCST90001545 | 0.23 | -0.0262 | 0.0335 | 0.434 | -0.114 | 1 | Non-significant |
| Psoriasis vulgaris | ebi-a-GCST90001579 | -0.244 | 0.024 | 0.0307 | 0.435 | -0.0984 | 1 | Non-significant |
| Type 1 diabetes | ebi-a-GCST90001823 | 0.23 | -0.0159 | 0.0204 | 0.436 | -0.0693 | 1 | Non-significant |
| Psoriasis vulgaris | ebi-a-GCST90001555 | -0.244 | -0.00825 | 0.0106 | 0.438 | 0.0338 | 1 | Non-significant |
| Psoriasis vulgaris | ebi-a-GCST90002009 | -0.244 | -0.0316 | 0.0407 | 0.438 | 0.13 | 1 | Non-significant |
| Psoriasis vulgaris | ebi-a-GCST90001545 | -0.244 | -0.0209 | 0.0269 | 0.438 | 0.0856 | 1 | Non-significant |
| Type 1 diabetes | ebi-a-GCST90002089 | 0.23 | -0.0193 | 0.0249 | 0.439 | -0.0839 | 1 | Non-significant |
| Type 1 diabetes | ebi-a-GCST90001857 | 0.23 | -0.00904 | 0.0117 | 0.439 | -0.0393 | 1 | Non-significant |
| Type 1 diabetes | ebi-a-GCST90001801 | 0.23 | 0.00705 | 0.00914 | 0.441 | 0.0307 | 1 | Non-significant |
| Psoriasis vulgaris | ebi-a-GCST90001806 | -0.244 | -0.00841 | 0.0109 | 0.441 | 0.0345 | 1 | Non-significant |
| Psoriasis vulgaris | ebi-a-GCST90001678 | -0.244 | 0.0605 | 0.0786 | 0.441 | -0.248 | 1 | Non-significant |
| Psoriasis vulgaris | ebi-a-GCST90001541 | -0.244 | 0.0144 | 0.0187 | 0.442 | -0.0589 | 1 | Non-significant |
| Psoriasis vulgaris | ebi-a-GCST90001969 | -0.244 | 0.018 | 0.0235 | 0.443 | -0.0737 | 1 | Non-significant |
| Psoriasis vulgaris | ebi-a-GCST90001659 | -0.244 | -0.00409 | 0.00535 | 0.444 | 0.0168 | 1 | Non-significant |
| Type 1 diabetes | ebi-a-GCST90001860 | 0.23 | -0.0084 | 0.011 | 0.445 | -0.0365 | 1 | Non-significant |
| Type 1 diabetes | ebi-a-GCST90002055 | 0.23 | -0.0176 | 0.0231 | 0.446 | -0.0767 | 1 | Non-significant |
| Type 1 diabetes | ebi-a-GCST90001398 | 0.23 | 0.0426 | 0.0561 | 0.447 | 0.186 | 1 | Non-significant |
| Type 1 diabetes | ebi-a-GCST90001701 | 0.23 | 0.011 | 0.0145 | 0.447 | 0.0479 | 1 | Non-significant |
| Type 1 diabetes | ebi-a-GCST90001689 | 0.23 | -0.00882 | 0.0116 | 0.447 | -0.0384 | 1 | Non-significant |
| Psoriasis vulgaris | ebi-a-GCST90001937 | -0.244 | 0.123 | 0.162 | 0.448 | -0.505 | 1 | Non-significant |
| Type 1 diabetes | ebi-a-GCST90001856 | 0.23 | -0.00617 | 0.00816 | 0.449 | -0.0269 | 1 | Non-significant |
| Type 1 diabetes | ebi-a-GCST90001897 | 0.23 | -0.00484 | 0.00644 | 0.452 | -0.0211 | 1 | Non-significant |
| Type 1 diabetes | ebi-a-GCST90001431 | 0.23 | 0.0567 | 0.0757 | 0.454 | 0.247 | 1 | Non-significant |
| Psoriasis vulgaris | ebi-a-GCST90001929 | -0.244 | -0.027 | 0.0362 | 0.455 | 0.111 | 1 | Non-significant |
| Type 1 diabetes | ebi-a-GCST90001417 | 0.23 | 0.0108 | 0.0145 | 0.455 | 0.047 | 1 | Non-significant |
| Psoriasis vulgaris | ebi-a-GCST90001733 | -0.244 | 0.0409 | 0.0549 | 0.456 | -0.168 | 1 | Non-significant |
| Psoriasis vulgaris | ebi-a-GCST90001872 | -0.244 | 0.0434 | 0.0583 | 0.457 | -0.178 | 1 | Non-significant |
| Psoriasis vulgaris | ebi-a-GCST90001708 | -0.244 | 0.00849 | 0.0114 | 0.457 | -0.0348 | 1 | Non-significant |
| Psoriasis vulgaris | ebi-a-GCST90001849 | -0.244 | -0.0113 | 0.0152 | 0.458 | 0.0462 | 1 | Non-significant |
| Type 1 diabetes | ebi-a-GCST90001451 | 0.23 | 0.0248 | 0.0335 | 0.458 | 0.108 | 1 | Non-significant |
| Type 1 diabetes | ebi-a-GCST90001907 | 0.23 | 0.0109 | 0.0148 | 0.459 | 0.0476 | 1 | Non-significant |
| Psoriasis vulgaris | ebi-a-GCST90001497 | -0.244 | -0.00376 | 0.00511 | 0.461 | 0.0154 | 1 | Non-significant |
| Psoriasis vulgaris | ebi-a-GCST90001544 | -0.244 | 0.0147 | 0.0202 | 0.466 | -0.0604 | 1 | Non-significant |
| Type 1 diabetes | ebi-a-GCST90001872 | 0.23 | -0.0294 | 0.0405 | 0.467 | -0.128 | 1 | Non-significant |
| Psoriasis vulgaris | ebi-a-GCST90001561 | -0.244 | -0.00262 | 0.00361 | 0.468 | 0.0107 | 1 | Non-significant |
| Type 1 diabetes | ebi-a-GCST90002063 | 0.23 | 0.0299 | 0.0412 | 0.468 | 0.13 | 1 | Non-significant |
| Type 1 diabetes | ebi-a-GCST90002061 | 0.23 | 0.00737 | 0.0102 | 0.468 | 0.0321 | 1 | Non-significant |
| Type 1 diabetes | ebi-a-GCST90002064 | 0.23 | 0.0392 | 0.0541 | 0.469 | 0.17 | 1 | Non-significant |
| Psoriasis vulgaris | ebi-a-GCST90001465 | -0.244 | -0.00913 | 0.0126 | 0.47 | 0.0375 | 1 | Non-significant |
| Type 1 diabetes | ebi-a-GCST90001478 | 0.23 | 0.0355 | 0.0493 | 0.471 | 0.155 | 1 | Non-significant |
| Psoriasis vulgaris | ebi-a-GCST90001426 | -0.244 | -0.0331 | 0.0459 | 0.471 | 0.136 | 1 | Non-significant |
| Psoriasis vulgaris | ebi-a-GCST90001462 | -0.244 | 0.0086 | 0.0119 | 0.471 | -0.0353 | 1 | Non-significant |
| Type 1 diabetes | ebi-a-GCST90002066 | 0.23 | 0.0275 | 0.0382 | 0.472 | 0.12 | 1 | Non-significant |
| Type 1 diabetes | ebi-a-GCST90001743 | 0.23 | 0.0213 | 0.0297 | 0.473 | 0.0927 | 1 | Non-significant |
| Type 1 diabetes | ebi-a-GCST90001468 | 0.23 | -0.00672 | 0.00939 | 0.474 | -0.0293 | 1 | Non-significant |
| Type 1 diabetes | ebi-a-GCST90002070 | 0.23 | 0.0275 | 0.0384 | 0.475 | 0.12 | 1 | Non-significant |
| Type 1 diabetes | ebi-a-GCST90002023 | 0.23 | 0.0171 | 0.0239 | 0.476 | 0.0743 | 1 | Non-significant |
| Type 1 diabetes | ebi-a-GCST90001584 | 0.23 | -0.0137 | 0.0193 | 0.478 | -0.0596 | 1 | Non-significant |
| Type 1 diabetes | ebi-a-GCST90001580 | 0.23 | 0.0132 | 0.0186 | 0.479 | 0.0575 | 1 | Non-significant |
| Type 1 diabetes | ebi-a-GCST90001526 | 0.23 | 0.00694 | 0.00983 | 0.48 | 0.0302 | 1 | Non-significant |
| Type 1 diabetes | ebi-a-GCST90001806 | 0.23 | 0.00561 | 0.00795 | 0.48 | 0.0244 | 1 | Non-significant |
| Type 1 diabetes | ebi-a-GCST90001927 | 0.23 | 0.0187 | 0.0265 | 0.481 | 0.0814 | 1 | Non-significant |
| Type 1 diabetes | ebi-a-GCST90001833 | 0.23 | -0.0179 | 0.0254 | 0.481 | -0.0778 | 1 | Non-significant |
| Type 1 diabetes | ebi-a-GCST90001462 | 0.23 | -0.00657 | 0.00934 | 0.482 | -0.0286 | 1 | Non-significant |
| Type 1 diabetes | ebi-a-GCST90002004 | 0.23 | 0.0152 | 0.0217 | 0.483 | 0.0662 | 1 | Non-significant |
| Psoriasis vulgaris | ebi-a-GCST90001911 | -0.244 | 0.0193 | 0.0275 | 0.483 | -0.0791 | 1 | Non-significant |
| Type 1 diabetes | ebi-a-GCST90001720 | 0.23 | -0.00339 | 0.00485 | 0.484 | -0.0148 | 1 | Non-significant |
| Type 1 diabetes | ebi-a-GCST90001847 | 0.23 | -0.00884 | 0.0127 | 0.487 | -0.0385 | 1 | Non-significant |
| Type 1 diabetes | ebi-a-GCST90001826 | 0.23 | -0.0112 | 0.0162 | 0.489 | -0.0487 | 1 | Non-significant |
| Psoriasis vulgaris | ebi-a-GCST90001720 | -0.244 | 0.00666 | 0.00963 | 0.489 | -0.0273 | 1 | Non-significant |
| Type 1 diabetes | ebi-a-GCST90001722 | 0.23 | 0.0273 | 0.0395 | 0.489 | 0.119 | 1 | Non-significant |
| Psoriasis vulgaris | ebi-a-GCST90001919 | -0.244 | 0.026 | 0.0379 | 0.492 | -0.107 | 1 | Non-significant |
| Psoriasis vulgaris | ebi-a-GCST90001982 | -0.244 | -0.0261 | 0.038 | 0.493 | 0.107 | 1 | Non-significant |
| Type 1 diabetes | ebi-a-GCST90001666 | 0.23 | -0.00077 | 0.00112 | 0.493 | -0.00334 | 1 | Non-significant |
| Type 1 diabetes | ebi-a-GCST90001719 | 0.23 | -0.00369 | 0.0054 | 0.494 | -0.0161 | 1 | Non-significant |
| Psoriasis vulgaris | ebi-a-GCST90001463 | -0.244 | 0.0124 | 0.0182 | 0.496 | -0.0508 | 1 | Non-significant |
| Psoriasis vulgaris | ebi-a-GCST90001397 | -0.244 | -0.0287 | 0.0422 | 0.496 | 0.118 | 1 | Non-significant |
| Type 1 diabetes | ebi-a-GCST90001820 | 0.23 | -0.00995 | 0.0146 | 0.496 | -0.0433 | 1 | Non-significant |
| Type 1 diabetes | ebi-a-GCST90001433 | 0.23 | -0.0188 | 0.0276 | 0.496 | -0.0817 | 1 | Non-significant |
| Psoriasis vulgaris | ebi-a-GCST90001574 | -0.244 | -0.0207 | 0.0304 | 0.497 | 0.0847 | 1 | Non-significant |
| Type 1 diabetes | ebi-a-GCST90002080 | 0.23 | 0.0438 | 0.0647 | 0.498 | 0.191 | 1 | Non-significant |
| Type 1 diabetes | ebi-a-GCST90002054 | 0.23 | -0.0127 | 0.0188 | 0.499 | -0.0553 | 1 | Non-significant |
| Type 1 diabetes | ebi-a-GCST90002068 | 0.23 | 0.0219 | 0.0325 | 0.5 | 0.0952 | 1 | Non-significant |
| Type 1 diabetes | ebi-a-GCST90001509 | 0.23 | 0.00574 | 0.00855 | 0.502 | 0.025 | 1 | Non-significant |
| Type 1 diabetes | ebi-a-GCST90001986 | 0.23 | 0.0101 | 0.015 | 0.502 | 0.0438 | 1 | Non-significant |
| Type 1 diabetes | ebi-a-GCST90001628 | 0.23 | 0.00942 | 0.0141 | 0.503 | 0.041 | 1 | Non-significant |
| Psoriasis vulgaris | ebi-a-GCST90001638 | -0.244 | -0.0152 | 0.0226 | 0.503 | 0.0622 | 1 | Non-significant |
| Type 1 diabetes | ebi-a-GCST90001534 | 0.23 | -0.0128 | 0.0192 | 0.504 | -0.0558 | 1 | Non-significant |
| Type 1 diabetes | ebi-a-GCST90001551 | 0.23 | 0.022 | 0.033 | 0.505 | 0.0957 | 1 | Non-significant |
| Type 1 diabetes | ebi-a-GCST90001508 | 0.23 | 0.0057 | 0.00859 | 0.507 | 0.0248 | 1 | Non-significant |
| Type 1 diabetes | ebi-a-GCST90001865 | 0.23 | -0.0102 | 0.0154 | 0.508 | -0.0444 | 1 | Non-significant |
| Psoriasis vulgaris | ebi-a-GCST90001464 | -0.244 | -0.00782 | 0.0119 | 0.51 | 0.0321 | 1 | Non-significant |
| Type 1 diabetes | ebi-a-GCST90001540 | 0.23 | 0.0193 | 0.0294 | 0.511 | 0.0842 | 1 | Non-significant |
| Psoriasis vulgaris | ebi-a-GCST90001580 | -0.244 | 0.0146 | 0.0222 | 0.511 | -0.06 | 1 | Non-significant |
| Type 1 diabetes | ebi-a-GCST90001716 | 0.23 | -0.00341 | 0.0052 | 0.512 | -0.0148 | 1 | Non-significant |
| Psoriasis vulgaris | ebi-a-GCST90001981 | -0.244 | 0.00473 | 0.00727 | 0.515 | -0.0194 | 1 | Non-significant |
| Psoriasis vulgaris | ebi-a-GCST90001721 | -0.244 | -0.00998 | 0.0154 | 0.516 | 0.041 | 1 | Non-significant |
| Psoriasis vulgaris | ebi-a-GCST90001540 | -0.244 | 0.0138 | 0.0213 | 0.517 | -0.0565 | 1 | Non-significant |
| Psoriasis vulgaris | ebi-a-GCST90001457 | -0.244 | -0.0205 | 0.0317 | 0.518 | 0.0841 | 1 | Non-significant |
| Type 1 diabetes | ebi-a-GCST90001934 | 0.23 | 0.0154 | 0.0238 | 0.518 | 0.067 | 1 | Non-significant |
| Type 1 diabetes | ebi-a-GCST90001463 | 0.23 | -0.00897 | 0.0139 | 0.518 | -0.039 | 1 | Non-significant |
| Type 1 diabetes | ebi-a-GCST90002117 | 0.23 | -0.0135 | 0.0211 | 0.523 | -0.0587 | 1 | Non-significant |
| Type 1 diabetes | ebi-a-GCST90001624 | 0.23 | -0.0225 | 0.0353 | 0.524 | -0.098 | 1 | Non-significant |
| Type 1 diabetes | ebi-a-GCST90002028 | 0.23 | -0.0342 | 0.0537 | 0.524 | -0.149 | 1 | Non-significant |
| Psoriasis vulgaris | ebi-a-GCST90001577 | -0.244 | -0.0356 | 0.056 | 0.525 | 0.146 | 1 | Non-significant |
| Psoriasis vulgaris | ebi-a-GCST90002040 | -0.244 | 0.014 | 0.022 | 0.525 | -0.0574 | 1 | Non-significant |
| Psoriasis vulgaris | ebi-a-GCST90001584 | -0.244 | 0.0118 | 0.0187 | 0.527 | -0.0485 | 1 | Non-significant |
| Type 1 diabetes | ebi-a-GCST90001706 | 0.23 | -0.00376 | 0.00595 | 0.528 | -0.0163 | 1 | Non-significant |
| Type 1 diabetes | ebi-a-GCST90001684 | 0.23 | -0.0193 | 0.0306 | 0.529 | -0.0839 | 1 | Non-significant |
| Type 1 diabetes | ebi-a-GCST90001908 | 0.23 | 0.0149 | 0.0236 | 0.529 | 0.0648 | 1 | Non-significant |
| Psoriasis vulgaris | ebi-a-GCST90001546 | -0.244 | -0.0076 | 0.0121 | 0.53 | 0.0312 | 1 | Non-significant |
| Type 1 diabetes | ebi-a-GCST90001528 | 0.23 | 0.00615 | 0.00983 | 0.532 | 0.0268 | 1 | Non-significant |
| Type 1 diabetes | ebi-a-GCST90001559 | 0.23 | -0.0127 | 0.0203 | 0.532 | -0.0551 | 1 | Non-significant |
| Type 1 diabetes | ebi-a-GCST90001970 | 0.23 | -0.011 | 0.0177 | 0.533 | -0.0479 | 1 | Non-significant |
| Psoriasis vulgaris | ebi-a-GCST90001514 | -0.244 | 0.00715 | 0.0115 | 0.534 | -0.0293 | 1 | Non-significant |
| Type 1 diabetes | ebi-a-GCST90001452 | 0.23 | -0.0423 | 0.068 | 0.534 | -0.184 | 1 | Non-significant |
| Type 1 diabetes | ebi-a-GCST90001500 | 0.23 | 0.00473 | 0.00762 | 0.535 | 0.0206 | 1 | Non-significant |
| Psoriasis vulgaris | ebi-a-GCST90002070 | -0.244 | 0.0183 | 0.0296 | 0.536 | -0.0752 | 1 | Non-significant |
| Type 1 diabetes | ebi-a-GCST90002024 | 0.23 | 0.0221 | 0.0358 | 0.537 | 0.0963 | 1 | Non-significant |
| Type 1 diabetes | ebi-a-GCST90002027 | 0.23 | 0.0131 | 0.0213 | 0.538 | 0.0571 | 1 | Non-significant |
| Type 1 diabetes | ebi-a-GCST90002091 | 0.23 | -0.00799 | 0.013 | 0.538 | -0.0348 | 1 | Non-significant |
| Type 1 diabetes | ebi-a-GCST90001646 | 0.23 | 0.00535 | 0.0087 | 0.538 | 0.0233 | 1 | Non-significant |
| Psoriasis vulgaris | ebi-a-GCST90001642 | -0.244 | 0.0237 | 0.0387 | 0.541 | -0.0971 | 1 | Non-significant |
| Type 1 diabetes | ebi-a-GCST90002060 | 0.23 | -0.015 | 0.0246 | 0.541 | -0.0654 | 1 | Non-significant |
| Psoriasis vulgaris | ebi-a-GCST90002004 | -0.244 | -0.0121 | 0.0198 | 0.542 | 0.0496 | 1 | Non-significant |
| Type 1 diabetes | ebi-a-GCST90001469 | 0.23 | -0.0058 | 0.00949 | 0.542 | -0.0252 | 1 | Non-significant |
| Type 1 diabetes | ebi-a-GCST90001851 | 0.23 | -0.00767 | 0.0126 | 0.543 | -0.0334 | 1 | Non-significant |
| Type 1 diabetes | ebi-a-GCST90001547 | 0.23 | -0.00929 | 0.0153 | 0.545 | -0.0404 | 1 | Non-significant |
| Type 1 diabetes | ebi-a-GCST90001985 | 0.23 | -0.00523 | 0.00865 | 0.545 | -0.0228 | 1 | Non-significant |
| Type 1 diabetes | ebi-a-GCST90001672 | 0.23 | 0.00258 | 0.00428 | 0.546 | 0.0112 | 1 | Non-significant |
| Type 1 diabetes | ebi-a-GCST90001808 | 0.23 | -0.00356 | 0.0059 | 0.546 | -0.0155 | 1 | Non-significant |
| Psoriasis vulgaris | ebi-a-GCST90002097 | -0.244 | 0.0113 | 0.0188 | 0.549 | -0.0463 | 1 | Non-significant |
| Psoriasis vulgaris | ebi-a-GCST90002027 | -0.244 | -0.0141 | 0.0236 | 0.549 | 0.058 | 1 | Non-significant |
| Psoriasis vulgaris | ebi-a-GCST90002054 | -0.244 | -0.0137 | 0.0229 | 0.549 | 0.0564 | 1 | Non-significant |
| Type 1 diabetes | ebi-a-GCST90001548 | 0.23 | -0.018 | 0.0301 | 0.55 | -0.0783 | 1 | Non-significant |
| Psoriasis vulgaris | ebi-a-GCST90001865 | -0.244 | -0.0107 | 0.0179 | 0.55 | 0.0438 | 1 | Non-significant |
| Psoriasis vulgaris | ebi-a-GCST90001718 | -0.244 | 0.00403 | 0.00675 | 0.551 | -0.0165 | 1 | Non-significant |
| Type 1 diabetes | ebi-a-GCST90001780 | 0.23 | -0.0185 | 0.031 | 0.551 | -0.0803 | 1 | Non-significant |
| Psoriasis vulgaris | ebi-a-GCST90002066 | -0.244 | 0.0163 | 0.0274 | 0.551 | -0.0671 | 1 | Non-significant |
| Psoriasis vulgaris | ebi-a-GCST90002067 | -0.244 | 0.0147 | 0.0248 | 0.554 | -0.0601 | 1 | Non-significant |
| Type 1 diabetes | ebi-a-GCST90002100 | 0.23 | 0.00967 | 0.0165 | 0.557 | 0.0421 | 1 | Non-significant |
| Psoriasis vulgaris | ebi-a-GCST90002029 | -0.244 | 0.00997 | 0.017 | 0.558 | -0.0409 | 1 | Non-significant |
| Type 1 diabetes | ebi-a-GCST90001763 | 0.23 | 0.00884 | 0.0151 | 0.559 | 0.0385 | 1 | Non-significant |
| Type 1 diabetes | ebi-a-GCST90001670 | 0.23 | 0.00224 | 0.00384 | 0.559 | 0.00976 | 1 | Non-significant |
| Psoriasis vulgaris | ebi-a-GCST90002063 | -0.244 | 0.0155 | 0.0266 | 0.56 | -0.0638 | 1 | Non-significant |
| Psoriasis vulgaris | ebi-a-GCST90001757 | -0.244 | -0.0145 | 0.025 | 0.561 | 0.0595 | 1 | Non-significant |
| Type 1 diabetes | ebi-a-GCST90001397 | 0.23 | 0.0156 | 0.0271 | 0.566 | 0.0677 | 1 | Non-significant |
| Type 1 diabetes | ebi-a-GCST90001937 | 0.23 | -0.0903 | 0.157 | 0.566 | -0.393 | 1 | Non-significant |
| Type 1 diabetes | ebi-a-GCST90002006 | 0.23 | 0.00853 | 0.0149 | 0.566 | 0.0371 | 1 | Non-significant |
| Type 1 diabetes | ebi-a-GCST90001987 | 0.23 | 0.00733 | 0.0129 | 0.57 | 0.0319 | 1 | Non-significant |
| Type 1 diabetes | ebi-a-GCST90001982 | 0.23 | -0.0163 | 0.0288 | 0.57 | -0.0711 | 1 | Non-significant |
| Type 1 diabetes | ebi-a-GCST90001948 | 0.23 | -0.00247 | 0.00436 | 0.57 | -0.0108 | 1 | Non-significant |
| Psoriasis vulgaris | ebi-a-GCST90001637 | -0.244 | -0.0126 | 0.0223 | 0.571 | 0.0519 | 1 | Non-significant |
| Psoriasis vulgaris | ebi-a-GCST90002023 | -0.244 | -0.0142 | 0.0251 | 0.571 | 0.0584 | 1 | Non-significant |
| Type 1 diabetes | ebi-a-GCST90001642 | 0.23 | 0.0155 | 0.0275 | 0.572 | 0.0676 | 1 | Non-significant |
| Type 1 diabetes | ebi-a-GCST90001639 | 0.23 | 0.0123 | 0.0218 | 0.573 | 0.0536 | 1 | Non-significant |
| Psoriasis vulgaris | ebi-a-GCST90001568 | -0.244 | -0.0531 | 0.094 | 0.573 | 0.218 | 1 | Non-significant |
| Type 1 diabetes | ebi-a-GCST90001558 | 0.23 | -0.0099 | 0.0176 | 0.575 | -0.0431 | 1 | Non-significant |
| Psoriasis vulgaris | ebi-a-GCST90001710 | -0.244 | 0.00494 | 0.00886 | 0.577 | -0.0203 | 1 | Non-significant |
| Type 1 diabetes | ebi-a-GCST90002074 | 0.23 | 0.003 | 0.00538 | 0.577 | 0.0131 | 1 | Non-significant |
| Psoriasis vulgaris | ebi-a-GCST90001829 | -0.244 | 0.00531 | 0.00953 | 0.577 | -0.0218 | 1 | Non-significant |
| Psoriasis vulgaris | ebi-a-GCST90002110 | -0.244 | -0.021 | 0.038 | 0.58 | 0.0863 | 1 | Non-significant |
| Type 1 diabetes | ebi-a-GCST90001629 | 0.23 | 0.009 | 0.0163 | 0.581 | 0.0392 | 1 | Non-significant |
| Type 1 diabetes | ebi-a-GCST90001798 | 0.23 | 0.00434 | 0.0079 | 0.583 | 0.0189 | 1 | Non-significant |
| Psoriasis vulgaris | ebi-a-GCST90001784 | -0.244 | 0.00948 | 0.0173 | 0.583 | -0.0389 | 1 | Non-significant |
| Psoriasis vulgaris | ebi-a-GCST90001634 | -0.244 | -0.0149 | 0.0272 | 0.584 | 0.0611 | 1 | Non-significant |
| Psoriasis vulgaris | ebi-a-GCST90002033 | -0.244 | -0.0139 | 0.0254 | 0.584 | 0.0572 | 1 | Non-significant |
| Type 1 diabetes | ebi-a-GCST90001905 | 0.23 | -0.0143 | 0.0262 | 0.585 | -0.0623 | 1 | Non-significant |
| Type 1 diabetes | ebi-a-GCST90001473 | 0.23 | -0.0045 | 0.00825 | 0.586 | -0.0196 | 1 | Non-significant |
| Psoriasis vulgaris | ebi-a-GCST90001525 | -0.244 | 0.0283 | 0.0521 | 0.587 | -0.116 | 1 | Non-significant |
| Type 1 diabetes | ebi-a-GCST90001917 | 0.23 | -0.0038 | 0.00703 | 0.589 | -0.0166 | 1 | Non-significant |
| Psoriasis vulgaris | ebi-a-GCST90001658 | -0.244 | -0.00215 | 0.00398 | 0.59 | 0.00881 | 1 | Non-significant |
| Psoriasis vulgaris | ebi-a-GCST90001459 | -0.244 | 0.0092 | 0.0171 | 0.59 | -0.0377 | 1 | Non-significant |
| Psoriasis vulgaris | ebi-a-GCST90001560 | -0.244 | 0.0104 | 0.0194 | 0.592 | -0.0426 | 1 | Non-significant |
| Psoriasis vulgaris | ebi-a-GCST90001624 | -0.244 | 0.0102 | 0.0191 | 0.595 | -0.0417 | 1 | Non-significant |
| Type 1 diabetes | ebi-a-GCST90002111 | 0.23 | -0.0103 | 0.0194 | 0.596 | -0.0448 | 1 | Non-significant |
| Psoriasis vulgaris | ebi-a-GCST90001820 | -0.244 | -0.00315 | 0.00597 | 0.597 | 0.0129 | 1 | Non-significant |
| Psoriasis vulgaris | ebi-a-GCST90001989 | -0.244 | 0.00275 | 0.00522 | 0.597 | -0.0113 | 1 | Non-significant |
| Type 1 diabetes | ebi-a-GCST90001649 | 0.23 | 0.00655 | 0.0124 | 0.598 | 0.0285 | 1 | Non-significant |
| Psoriasis vulgaris | ebi-a-GCST90001748 | -0.244 | -0.015 | 0.0284 | 0.598 | 0.0615 | 1 | Non-significant |
| Type 1 diabetes | ebi-a-GCST90002096 | 0.23 | 0.00669 | 0.0127 | 0.6 | 0.0291 | 1 | Non-significant |
| Psoriasis vulgaris | ebi-a-GCST90001643 | -0.244 | 0.00406 | 0.00774 | 0.6 | -0.0167 | 1 | Non-significant |
| Type 1 diabetes | ebi-a-GCST90001864 | 0.23 | -0.00681 | 0.013 | 0.6 | -0.0296 | 1 | Non-significant |
| Type 1 diabetes | ebi-a-GCST90001586 | 0.23 | -0.00668 | 0.0128 | 0.601 | -0.0291 | 1 | Non-significant |
| Psoriasis vulgaris | ebi-a-GCST90001596 | -0.244 | -0.026 | 0.0497 | 0.601 | 0.107 | 1 | Non-significant |
| Type 1 diabetes | ebi-a-GCST90002035 | 0.23 | -0.00945 | 0.0181 | 0.601 | -0.0411 | 1 | Non-significant |
| Psoriasis vulgaris | ebi-a-GCST90001433 | -0.244 | -0.0145 | 0.0278 | 0.602 | 0.0596 | 1 | Non-significant |
| Psoriasis vulgaris | ebi-a-GCST90001646 | -0.244 | 0.00555 | 0.0107 | 0.603 | -0.0228 | 1 | Non-significant |
| Psoriasis vulgaris | ebi-a-GCST90001542 | -0.244 | 0.0101 | 0.0195 | 0.603 | -0.0416 | 1 | Non-significant |
| Type 1 diabetes | ebi-a-GCST90001560 | 0.23 | 0.00974 | 0.0188 | 0.604 | 0.0424 | 1 | Non-significant |
| Type 1 diabetes | ebi-a-GCST90001587 | 0.23 | -0.00699 | 0.0135 | 0.604 | -0.0304 | 1 | Non-significant |
| Psoriasis vulgaris | ebi-a-GCST90001607 | -0.244 | -0.014 | 0.0271 | 0.604 | 0.0576 | 1 | Non-significant |
| Psoriasis vulgaris | ebi-a-GCST90001761 | -0.244 | -0.0119 | 0.0231 | 0.605 | 0.0489 | 1 | Non-significant |
| Psoriasis vulgaris | ebi-a-GCST90002095 | -0.244 | 0.00818 | 0.0158 | 0.606 | -0.0336 | 1 | Non-significant |
| Type 1 diabetes | ebi-a-GCST90001652 | 0.23 | 0.0222 | 0.0431 | 0.606 | 0.0967 | 1 | Non-significant |
| Type 1 diabetes | ebi-a-GCST90001655 | 0.23 | -0.0288 | 0.0558 | 0.606 | -0.125 | 1 | Non-significant |
| Type 1 diabetes | ebi-a-GCST90001568 | 0.23 | -0.0158 | 0.0309 | 0.608 | -0.069 | 1 | Non-significant |
| Psoriasis vulgaris | ebi-a-GCST90002016 | -0.244 | -0.00708 | 0.0138 | 0.608 | 0.0291 | 1 | Non-significant |
| Psoriasis vulgaris | ebi-a-GCST90002015 | -0.244 | -0.00708 | 0.0138 | 0.608 | 0.029 | 1 | Non-significant |
| Type 1 diabetes | ebi-a-GCST90001632 | 0.23 | 0.024 | 0.0469 | 0.608 | 0.105 | 1 | Non-significant |
| Psoriasis vulgaris | ebi-a-GCST90001807 | -0.244 | 0.0208 | 0.0407 | 0.609 | -0.0855 | 1 | Non-significant |
| Psoriasis vulgaris | ebi-a-GCST90001928 | -0.244 | -0.0071 | 0.0139 | 0.609 | 0.0291 | 1 | Non-significant |
| Psoriasis vulgaris | ebi-a-GCST90002028 | -0.244 | 0.0214 | 0.0418 | 0.609 | -0.0876 | 1 | Non-significant |
| Psoriasis vulgaris | ebi-a-GCST90002072 | -0.244 | -0.0101 | 0.0197 | 0.61 | 0.0413 | 1 | Non-significant |
| Psoriasis vulgaris | ebi-a-GCST90001626 | -0.244 | 0.0119 | 0.0233 | 0.611 | -0.0487 | 1 | Non-significant |
| Type 1 diabetes | ebi-a-GCST90002025 | 0.23 | 0.00644 | 0.0126 | 0.611 | 0.028 | 1 | Non-significant |
| Type 1 diabetes | ebi-a-GCST90001846 | 0.23 | -0.00647 | 0.0128 | 0.613 | -0.0282 | 1 | Non-significant |
| Type 1 diabetes | ebi-a-GCST90001647 | 0.23 | 0.00582 | 0.0115 | 0.613 | 0.0253 | 1 | Non-significant |
| Psoriasis vulgaris | ebi-a-GCST90001823 | -0.244 | -0.00491 | 0.00972 | 0.613 | 0.0201 | 1 | Non-significant |
| Psoriasis vulgaris | ebi-a-GCST90001841 | -0.244 | 0.00395 | 0.00783 | 0.614 | -0.0162 | 1 | Non-significant |
| Psoriasis vulgaris | ebi-a-GCST90001940 | -0.244 | -0.0133 | 0.0264 | 0.615 | 0.0546 | 1 | Non-significant |
| Psoriasis vulgaris | ebi-a-GCST90002034 | -0.244 | 0.02 | 0.0399 | 0.616 | -0.0821 | 1 | Non-significant |
| Type 1 diabetes | ebi-a-GCST90001740 | 0.23 | 0.0218 | 0.0435 | 0.616 | 0.0949 | 1 | Non-significant |
| Psoriasis vulgaris | ebi-a-GCST90002091 | -0.244 | 0.00765 | 0.0153 | 0.617 | -0.0314 | 1 | Non-significant |
| Type 1 diabetes | ebi-a-GCST90001626 | 0.23 | -0.0116 | 0.0232 | 0.618 | -0.0504 | 1 | Non-significant |
| Psoriasis vulgaris | ebi-a-GCST90001780 | -0.244 | 0.00812 | 0.0163 | 0.618 | -0.0333 | 1 | Non-significant |
| Psoriasis vulgaris | ebi-a-GCST90001655 | -0.244 | -0.0208 | 0.0417 | 0.619 | 0.0851 | 1 | Non-significant |
| Psoriasis vulgaris | ebi-a-GCST90001592 | -0.244 | -0.0135 | 0.0273 | 0.62 | 0.0554 | 1 | Non-significant |
| Psoriasis vulgaris | ebi-a-GCST90001660 | -0.244 | -0.00372 | 0.00752 | 0.621 | 0.0153 | 1 | Non-significant |
| Psoriasis vulgaris | ebi-a-GCST90001599 | -0.244 | -0.0163 | 0.033 | 0.621 | 0.0669 | 1 | Non-significant |
| Type 1 diabetes | ebi-a-GCST90002034 | 0.23 | -0.0159 | 0.0322 | 0.622 | -0.0693 | 1 | Non-significant |
| Type 1 diabetes | ebi-a-GCST90002105 | 0.23 | 0.00641 | 0.013 | 0.622 | 0.0279 | 1 | Non-significant |
| Psoriasis vulgaris | ebi-a-GCST90001885 | -0.244 | 0.0205 | 0.0416 | 0.623 | -0.0841 | 1 | Non-significant |
| Psoriasis vulgaris | ebi-a-GCST90001499 | -0.244 | 0.00314 | 0.00639 | 0.623 | -0.0129 | 1 | Non-significant |
| Psoriasis vulgaris | ebi-a-GCST90002094 | -0.244 | -0.00993 | 0.0202 | 0.624 | 0.0407 | 1 | Non-significant |
| Type 1 diabetes | ebi-a-GCST90001546 | 0.23 | -0.0069 | 0.0141 | 0.625 | -0.03 | 1 | Non-significant |
| Psoriasis vulgaris | ebi-a-GCST90001493 | -0.244 | 0.00306 | 0.00625 | 0.625 | -0.0125 | 1 | Non-significant |
| Psoriasis vulgaris | ebi-a-GCST90001635 | -0.244 | -0.0126 | 0.0259 | 0.627 | 0.0516 | 1 | Non-significant |
| Type 1 diabetes | ebi-a-GCST90001751 | 0.23 | 0.0123 | 0.0254 | 0.627 | 0.0536 | 1 | Non-significant |
| Psoriasis vulgaris | ebi-a-GCST90002077 | -0.244 | 0.0119 | 0.0248 | 0.629 | -0.049 | 1 | Non-significant |
| Psoriasis vulgaris | ebi-a-GCST90001460 | -0.244 | -0.0105 | 0.0219 | 0.633 | 0.043 | 1 | Non-significant |
| Psoriasis vulgaris | ebi-a-GCST90001719 | -0.244 | 0.00481 | 0.0101 | 0.633 | -0.0197 | 1 | Non-significant |
| Psoriasis vulgaris | ebi-a-GCST90001805 | -0.244 | -0.00326 | 0.00682 | 0.633 | 0.0134 | 1 | Non-significant |
| Type 1 diabetes | ebi-a-GCST90001410 | 0.23 | -0.0136 | 0.0284 | 0.633 | -0.0591 | 1 | Non-significant |
| Psoriasis vulgaris | ebi-a-GCST90001715 | -0.244 | 0.00262 | 0.0055 | 0.634 | -0.0107 | 1 | Non-significant |
| Psoriasis vulgaris | ebi-a-GCST90001866 | -0.244 | 0.00675 | 0.0142 | 0.634 | -0.0277 | 1 | Non-significant |
| Psoriasis vulgaris | ebi-a-GCST90002042 | -0.244 | 0.0139 | 0.0292 | 0.635 | -0.0569 | 1 | Non-significant |
| Psoriasis vulgaris | ebi-a-GCST90002057 | -0.244 | -0.0106 | 0.0224 | 0.636 | 0.0436 | 1 | Non-significant |
| Psoriasis vulgaris | ebi-a-GCST90001898 | -0.244 | 0.00632 | 0.0134 | 0.638 | -0.0259 | 1 | Non-significant |
| Type 1 diabetes | ebi-a-GCST90001929 | 0.23 | 0.00587 | 0.0125 | 0.64 | 0.0255 | 1 | Non-significant |
| Psoriasis vulgaris | ebi-a-GCST90002068 | -0.244 | 0.0104 | 0.0224 | 0.641 | -0.0429 | 1 | Non-significant |
| Type 1 diabetes | ebi-a-GCST90001725 | 0.23 | 0.0031 | 0.00665 | 0.641 | 0.0135 | 1 | Non-significant |
| Type 1 diabetes | ebi-a-GCST90001784 | 0.23 | -0.0119 | 0.0256 | 0.643 | -0.0517 | 1 | Non-significant |
| Type 1 diabetes | ebi-a-GCST90002072 | 0.23 | 0.00787 | 0.017 | 0.644 | 0.0343 | 1 | Non-significant |
| Psoriasis vulgaris | ebi-a-GCST90001586 | -0.244 | 0.00615 | 0.0133 | 0.644 | -0.0252 | 1 | Non-significant |
| Psoriasis vulgaris | ebi-a-GCST90001709 | -0.244 | 0.00487 | 0.0105 | 0.644 | -0.02 | 1 | Non-significant |
| Psoriasis vulgaris | ebi-a-GCST90001415 | -0.244 | -0.0125 | 0.0272 | 0.644 | 0.0514 | 1 | Non-significant |
| Psoriasis vulgaris | ebi-a-GCST90001412 | -0.244 | 0.0474 | 0.103 | 0.644 | -0.195 | 1 | Non-significant |
| Type 1 diabetes | ebi-a-GCST90001554 | 0.23 | 0.00395 | 0.00856 | 0.645 | 0.0172 | 1 | Non-significant |
| Type 1 diabetes | ebi-a-GCST90001634 | 0.23 | -0.00311 | 0.00675 | 0.645 | -0.0135 | 1 | Non-significant |
| Psoriasis vulgaris | ebi-a-GCST90001845 | -0.244 | 0.0038 | 0.00825 | 0.645 | -0.0156 | 1 | Non-significant |
| Type 1 diabetes | ebi-a-GCST90001950 | 0.23 | -0.00291 | 0.00633 | 0.646 | -0.0127 | 1 | Non-significant |
| Psoriasis vulgaris | ebi-a-GCST90001905 | -0.244 | 0.0114 | 0.0247 | 0.646 | -0.0466 | 1 | Non-significant |
| Psoriasis vulgaris | ebi-a-GCST90001860 | -0.244 | 0.00645 | 0.0141 | 0.646 | -0.0265 | 1 | Non-significant |
| Psoriasis vulgaris | ebi-a-GCST90001458 | -0.244 | 0.00562 | 0.0123 | 0.648 | -0.0231 | 1 | Non-significant |
| Psoriasis vulgaris | ebi-a-GCST90001716 | -0.244 | 0.00452 | 0.0099 | 0.648 | -0.0185 | 1 | Non-significant |
| Psoriasis vulgaris | ebi-a-GCST90001763 | -0.244 | 0.00675 | 0.0148 | 0.649 | -0.0277 | 1 | Non-significant |
| Psoriasis vulgaris | ebi-a-GCST90001558 | -0.244 | 0.005 | 0.011 | 0.649 | -0.0205 | 1 | Non-significant |
| Type 1 diabetes | ebi-a-GCST90001472 | 0.23 | -0.00361 | 0.00793 | 0.649 | -0.0157 | 1 | Non-significant |
| Psoriasis vulgaris | ebi-a-GCST90001894 | -0.244 | 0.00523 | 0.0115 | 0.649 | -0.0215 | 1 | Non-significant |
| Psoriasis vulgaris | ebi-a-GCST90001527 | -0.244 | 0.0112 | 0.0247 | 0.65 | -0.0461 | 1 | Non-significant |
| Type 1 diabetes | ebi-a-GCST90001894 | 0.23 | 0.00732 | 0.0162 | 0.651 | 0.0318 | 1 | Non-significant |
| Psoriasis vulgaris | ebi-a-GCST90001787 | -0.244 | -0.00644 | 0.0143 | 0.652 | 0.0264 | 1 | Non-significant |
| Type 1 diabetes | ebi-a-GCST90001911 | 0.23 | 0.00732 | 0.0162 | 0.652 | 0.0318 | 1 | Non-significant |
| Psoriasis vulgaris | ebi-a-GCST90001572 | -0.244 | -0.0449 | 0.0996 | 0.652 | 0.184 | 1 | Non-significant |
| Psoriasis vulgaris | ebi-a-GCST90001535 | -0.244 | -0.00365 | 0.00814 | 0.654 | 0.015 | 1 | Non-significant |
| Psoriasis vulgaris | ebi-a-GCST90001722 | -0.244 | -0.0137 | 0.0306 | 0.654 | 0.0563 | 1 | Non-significant |
| Psoriasis vulgaris | ebi-a-GCST90001587 | -0.244 | 0.00588 | 0.0131 | 0.654 | -0.0241 | 1 | Non-significant |
| Psoriasis vulgaris | ebi-a-GCST90001906 | -0.244 | 0.0189 | 0.0424 | 0.655 | -0.0777 | 1 | Non-significant |
| Type 1 diabetes | ebi-a-GCST90001849 | 0.23 | -0.0031 | 0.00696 | 0.655 | -0.0135 | 1 | Non-significant |
| Type 1 diabetes | ebi-a-GCST90001713 | 0.23 | 0.00356 | 0.00798 | 0.656 | 0.0155 | 1 | Non-significant |
| Psoriasis vulgaris | ebi-a-GCST90001559 | -0.244 | 0.00736 | 0.0165 | 0.656 | -0.0302 | 1 | Non-significant |
| Psoriasis vulgaris | ebi-a-GCST90001670 | -0.244 | 0.00156 | 0.00349 | 0.656 | -0.00639 | 1 | Non-significant |
| Type 1 diabetes | ebi-a-GCST90001959 | 0.23 | 0.0113 | 0.0254 | 0.656 | 0.0493 | 1 | Non-significant |
| Type 1 diabetes | ebi-a-GCST90001486 | 0.23 | -0.00551 | 0.0124 | 0.656 | -0.024 | 1 | Non-significant |
| Psoriasis vulgaris | ebi-a-GCST90001979 | -0.244 | 0.00363 | 0.00815 | 0.656 | -0.0149 | 1 | Non-significant |
| Psoriasis vulgaris | ebi-a-GCST90001452 | -0.244 | 0.0291 | 0.0655 | 0.657 | -0.119 | 1 | Non-significant |
| Psoriasis vulgaris | ebi-a-GCST90001913 | -0.244 | 0.0148 | 0.0335 | 0.658 | -0.0608 | 1 | Non-significant |
| Psoriasis vulgaris | ebi-a-GCST90002073 | -0.244 | -0.00225 | 0.00508 | 0.658 | 0.00922 | 1 | Non-significant |
| Psoriasis vulgaris | ebi-a-GCST90001652 | -0.244 | 0.0148 | 0.0336 | 0.659 | -0.0609 | 1 | Non-significant |
| Psoriasis vulgaris | ebi-a-GCST90001791 | -0.244 | 0.00377 | 0.00858 | 0.66 | -0.0155 | 1 | Non-significant |
| Psoriasis vulgaris | ebi-a-GCST90001702 | -0.244 | 0.00229 | 0.00522 | 0.661 | -0.00941 | 1 | Non-significant |
| Type 1 diabetes | ebi-a-GCST90001787 | 0.23 | -0.0101 | 0.0229 | 0.661 | -0.0437 | 1 | Non-significant |
| Psoriasis vulgaris | ebi-a-GCST90001602 | -0.244 | 0.0158 | 0.036 | 0.661 | -0.0647 | 1 | Non-significant |
| Type 1 diabetes | ebi-a-GCST90001812 | 0.23 | -0.00496 | 0.0113 | 0.662 | -0.0216 | 1 | Non-significant |
| Psoriasis vulgaris | ebi-a-GCST90001859 | -0.244 | -0.00527 | 0.0121 | 0.662 | 0.0216 | 1 | Non-significant |
| Psoriasis vulgaris | ebi-a-GCST90001967 | -0.244 | -0.0221 | 0.0507 | 0.662 | 0.0908 | 1 | Non-significant |
| Type 1 diabetes | ebi-a-GCST90001869 | 0.23 | 0.00381 | 0.00875 | 0.663 | 0.0166 | 1 | Non-significant |
| Psoriasis vulgaris | ebi-a-GCST90001684 | -0.244 | -0.0102 | 0.0233 | 0.663 | 0.0417 | 1 | Non-significant |
| Type 1 diabetes | ebi-a-GCST90001839 | 0.23 | -0.00618 | 0.0142 | 0.664 | -0.0269 | 1 | Non-significant |
| Type 1 diabetes | ebi-a-GCST90001533 | 0.23 | -0.00308 | 0.00711 | 0.665 | -0.0134 | 1 | Non-significant |
| Type 1 diabetes | ebi-a-GCST90002058 | 0.23 | -0.00956 | 0.0221 | 0.665 | -0.0416 | 1 | Non-significant |
| Type 1 diabetes | ebi-a-GCST90001653 | 0.23 | -0.0168 | 0.0389 | 0.665 | -0.0732 | 1 | Non-significant |
| Type 1 diabetes | ebi-a-GCST90001656 | 0.23 | -0.0168 | 0.0389 | 0.665 | -0.0732 | 1 | Non-significant |
| Psoriasis vulgaris | ebi-a-GCST90001800 | -0.244 | -0.00547 | 0.0127 | 0.666 | 0.0224 | 1 | Non-significant |
| Psoriasis vulgaris | ebi-a-GCST90001959 | -0.244 | -0.0108 | 0.025 | 0.667 | 0.0442 | 1 | Non-significant |
| Psoriasis vulgaris | ebi-a-GCST90001864 | -0.244 | -0.00537 | 0.0125 | 0.667 | 0.022 | 1 | Non-significant |
| Type 1 diabetes | ebi-a-GCST90001662 | 0.23 | -0.0128 | 0.0297 | 0.667 | -0.0557 | 1 | Non-significant |
| Psoriasis vulgaris | ebi-a-GCST90001883 | -0.244 | 0.00668 | 0.0155 | 0.667 | -0.0274 | 1 | Non-significant |
| Psoriasis vulgaris | ebi-a-GCST90001619 | -0.244 | 0.00298 | 0.00693 | 0.667 | -0.0122 | 1 | Non-significant |
| Type 1 diabetes | ebi-a-GCST90001401 | 0.23 | -0.00708 | 0.0165 | 0.668 | -0.0308 | 1 | Non-significant |
| Type 1 diabetes | ebi-a-GCST90001883 | 0.23 | 0.00467 | 0.0109 | 0.669 | 0.0203 | 1 | Non-significant |
| Psoriasis vulgaris | ebi-a-GCST90001486 | -0.244 | 0.00467 | 0.0109 | 0.669 | -0.0191 | 1 | Non-significant |
| Psoriasis vulgaris | ebi-a-GCST90001666 | -0.244 | 0.00046 | 0.00108 | 0.669 | -0.00189 | 1 | Non-significant |
| Type 1 diabetes | ebi-a-GCST90001838 | 0.23 | -0.00231 | 0.00542 | 0.67 | -0.0101 | 1 | Non-significant |
| Type 1 diabetes | ebi-a-GCST90001898 | 0.23 | 0.00587 | 0.0138 | 0.67 | 0.0256 | 1 | Non-significant |
| Psoriasis vulgaris | ebi-a-GCST90001653 | -0.244 | 0.0157 | 0.0369 | 0.67 | -0.0644 | 1 | Non-significant |
| Psoriasis vulgaris | ebi-a-GCST90001656 | -0.244 | 0.0157 | 0.0369 | 0.67 | -0.0644 | 1 | Non-significant |
| Psoriasis vulgaris | ebi-a-GCST90001509 | -0.244 | 0.00412 | 0.00969 | 0.67 | -0.0169 | 1 | Non-significant |
| Psoriasis vulgaris | ebi-a-GCST90001672 | -0.244 | 0.00133 | 0.00314 | 0.671 | -0.00546 | 1 | Non-significant |
| Type 1 diabetes | ebi-a-GCST90001852 | 0.23 | -0.0047 | 0.0111 | 0.672 | -0.0204 | 1 | Non-significant |
| Psoriasis vulgaris | ebi-a-GCST90001593 | -0.244 | 0.0183 | 0.0432 | 0.672 | -0.075 | 1 | Non-significant |
| Type 1 diabetes | ebi-a-GCST90001648 | 0.23 | 0.00552 | 0.0131 | 0.673 | 0.024 | 1 | Non-significant |
| Psoriasis vulgaris | ebi-a-GCST90002056 | -0.244 | -0.00706 | 0.0167 | 0.673 | 0.029 | 1 | Non-significant |
| Psoriasis vulgaris | ebi-a-GCST90002098 | -0.244 | -0.00389 | 0.00924 | 0.674 | 0.016 | 1 | Non-significant |
| Type 1 diabetes | ebi-a-GCST90002012 | 0.23 | -0.0125 | 0.0298 | 0.675 | -0.0544 | 1 | Non-significant |
| Psoriasis vulgaris | ebi-a-GCST90001812 | -0.244 | -0.00526 | 0.0125 | 0.675 | 0.0216 | 1 | Non-significant |
| Type 1 diabetes | ebi-a-GCST90001660 | 0.23 | 0.00222 | 0.00533 | 0.677 | 0.00966 | 1 | Non-significant |
| Psoriasis vulgaris | ebi-a-GCST90001521 | -0.244 | 0.0117 | 0.0281 | 0.677 | -0.048 | 1 | Non-significant |
| Type 1 diabetes | ebi-a-GCST90002042 | 0.23 | -0.00877 | 0.0211 | 0.677 | -0.0381 | 1 | Non-significant |
| Psoriasis vulgaris | ebi-a-GCST90002024 | -0.244 | -0.00631 | 0.0152 | 0.678 | 0.0259 | 1 | Non-significant |
| Psoriasis vulgaris | ebi-a-GCST90001581 | -0.244 | -0.00941 | 0.0227 | 0.679 | 0.0386 | 1 | Non-significant |
| Type 1 diabetes | ebi-a-GCST90001845 | 0.23 | -0.00263 | 0.00636 | 0.679 | -0.0114 | 1 | Non-significant |
| Psoriasis vulgaris | ebi-a-GCST90001818 | -0.244 | -0.0108 | 0.0262 | 0.679 | 0.0443 | 1 | Non-significant |
| Type 1 diabetes | ebi-a-GCST90001981 | 0.23 | 0.00241 | 0.00588 | 0.683 | 0.0105 | 1 | Non-significant |
| Type 1 diabetes | ebi-a-GCST90001643 | 0.23 | 0.00203 | 0.00496 | 0.683 | 0.00882 | 1 | Non-significant |
| Type 1 diabetes | ebi-a-GCST90001745 | 0.23 | 0.0128 | 0.0314 | 0.683 | 0.0557 | 1 | Non-significant |
| Psoriasis vulgaris | ebi-a-GCST90001838 | -0.244 | -0.00298 | 0.00731 | 0.683 | 0.0122 | 1 | Non-significant |
| Psoriasis vulgaris | ebi-a-GCST90001536 | -0.244 | 0.00404 | 0.00994 | 0.684 | -0.0166 | 1 | Non-significant |
| Type 1 diabetes | ebi-a-GCST90001949 | 0.23 | -0.00125 | 0.00308 | 0.685 | -0.00544 | 1 | Non-significant |
| Psoriasis vulgaris | ebi-a-GCST90001641 | -0.244 | 0.0118 | 0.029 | 0.685 | -0.0482 | 1 | Non-significant |
| Psoriasis vulgaris | ebi-a-GCST90001950 | -0.244 | 0.00266 | 0.00656 | 0.685 | -0.0109 | 1 | Non-significant |
| Type 1 diabetes | ebi-a-GCST90001712 | 0.23 | 0.00172 | 0.00424 | 0.685 | 0.00749 | 1 | Non-significant |
| Psoriasis vulgaris | ebi-a-GCST90002099 | -0.244 | -0.00206 | 0.00508 | 0.686 | 0.00844 | 1 | Non-significant |
| Type 1 diabetes | ebi-a-GCST90002090 | 0.23 | -0.00527 | 0.013 | 0.686 | -0.0229 | 1 | Non-significant |
| Type 1 diabetes | ebi-a-GCST90001429 | 0.23 | 0.0368 | 0.0913 | 0.687 | 0.16 | 1 | Non-significant |
| Psoriasis vulgaris | ebi-a-GCST90001636 | -0.244 | 0.0383 | 0.0952 | 0.688 | -0.157 | 1 | Non-significant |
| Type 1 diabetes | ebi-a-GCST90001758 | 0.23 | 0.00856 | 0.0213 | 0.688 | 0.0372 | 1 | Non-significant |
| Psoriasis vulgaris | ebi-a-GCST90001606 | -0.244 | 0.0118 | 0.0294 | 0.688 | -0.0483 | 1 | Non-significant |
| Psoriasis vulgaris | ebi-a-GCST90001410 | -0.244 | 0.00797 | 0.02 | 0.69 | -0.0327 | 1 | Non-significant |
| Type 1 diabetes | ebi-a-GCST90002108 | 0.23 | 0.0121 | 0.0305 | 0.69 | 0.0528 | 1 | Non-significant |
| Type 1 diabetes | ebi-a-GCST90001707 | 0.23 | 0.00242 | 0.00609 | 0.691 | 0.0105 | 1 | Non-significant |
| Type 1 diabetes | ebi-a-GCST90002120 | 0.23 | -0.00822 | 0.0207 | 0.691 | -0.0358 | 1 | Non-significant |
| Psoriasis vulgaris | ebi-a-GCST90001728 | -0.244 | 0.00744 | 0.0188 | 0.691 | -0.0305 | 1 | Non-significant |
| Type 1 diabetes | ebi-a-GCST90001802 | 0.23 | -0.00324 | 0.00817 | 0.692 | -0.0141 | 1 | Non-significant |
| Type 1 diabetes | ebi-a-GCST90001705 | 0.23 | -0.0017 | 0.00429 | 0.692 | -0.00741 | 1 | Non-significant |
| Type 1 diabetes | ebi-a-GCST90001687 | 0.23 | -0.00684 | 0.0173 | 0.693 | -0.0298 | 1 | Non-significant |
| Psoriasis vulgaris | ebi-a-GCST90001623 | -0.244 | -0.00413 | 0.0105 | 0.695 | 0.0169 | 1 | Non-significant |
| Type 1 diabetes | ebi-a-GCST90001693 | 0.23 | 0.00185 | 0.00473 | 0.696 | 0.00803 | 1 | Non-significant |
| Type 1 diabetes | ebi-a-GCST90001460 | 0.23 | 0.00685 | 0.0176 | 0.697 | 0.0298 | 1 | Non-significant |
| Psoriasis vulgaris | ebi-a-GCST90001628 | -0.244 | 0.00403 | 0.0103 | 0.697 | -0.0165 | 1 | Non-significant |
| Psoriasis vulgaris | ebi-a-GCST90001489 | -0.244 | -0.00264 | 0.00678 | 0.697 | 0.0108 | 1 | Non-significant |
| Type 1 diabetes | ebi-a-GCST90001502 | 0.23 | 0.000959 | 0.00248 | 0.698 | 0.00417 | 1 | Non-significant |
| Psoriasis vulgaris | ebi-a-GCST90001826 | -0.244 | -0.00219 | 0.00567 | 0.699 | 0.00899 | 1 | Non-significant |
| Type 1 diabetes | ebi-a-GCST90002002 | 0.23 | -0.00799 | 0.0207 | 0.7 | -0.0348 | 1 | Non-significant |
| Psoriasis vulgaris | ebi-a-GCST90002021 | -0.244 | 0.0111 | 0.0288 | 0.7 | -0.0456 | 1 | Non-significant |
| Psoriasis vulgaris | ebi-a-GCST90001663 | -0.244 | -0.012 | 0.031 | 0.7 | 0.049 | 1 | Non-significant |
| Type 1 diabetes | ebi-a-GCST90001702 | 0.23 | 0.00115 | 0.003 | 0.701 | 0.00501 | 1 | Non-significant |
| Psoriasis vulgaris | ebi-a-GCST90001662 | -0.244 | 0.0089 | 0.0232 | 0.701 | -0.0365 | 1 | Non-significant |
| Psoriasis vulgaris | ebi-a-GCST90002083 | -0.244 | -0.0143 | 0.0375 | 0.703 | 0.0586 | 1 | Non-significant |
| Type 1 diabetes | ebi-a-GCST90001623 | 0.23 | -0.00267 | 0.00701 | 0.704 | -0.0116 | 1 | Non-significant |
| Psoriasis vulgaris | ebi-a-GCST90001824 | -0.244 | 0.00293 | 0.00771 | 0.704 | -0.012 | 1 | Non-significant |
| Psoriasis vulgaris | ebi-a-GCST90001858 | -0.244 | -0.00219 | 0.00576 | 0.704 | 0.00897 | 1 | Non-significant |
| Psoriasis vulgaris | ebi-a-GCST90001474 | -0.244 | 0.012 | 0.0316 | 0.705 | -0.0491 | 1 | Non-significant |
| Type 1 diabetes | ebi-a-GCST90001635 | 0.23 | -0.00194 | 0.00512 | 0.705 | -0.00843 | 1 | Non-significant |
| Type 1 diabetes | ebi-a-GCST90001479 | 0.23 | 0.0143 | 0.0378 | 0.705 | 0.0623 | 1 | Non-significant |
| Type 1 diabetes | ebi-a-GCST90001853 | 0.23 | -0.00436 | 0.0115 | 0.705 | -0.019 | 1 | Non-significant |
| Type 1 diabetes | ebi-a-GCST90001504 | 0.23 | 0.00955 | 0.0253 | 0.706 | 0.0415 | 1 | Non-significant |
| Type 1 diabetes | ebi-a-GCST90001892 | 0.23 | -0.0016 | 0.00426 | 0.706 | -0.00698 | 1 | Non-significant |
| Type 1 diabetes | ebi-a-GCST90001943 | 0.23 | 0.0117 | 0.0311 | 0.707 | 0.051 | 1 | Non-significant |
| Psoriasis vulgaris | ebi-a-GCST90001801 | -0.244 | -0.00396 | 0.0105 | 0.707 | 0.0163 | 1 | Non-significant |
| Psoriasis vulgaris | ebi-a-GCST90001976 | -0.244 | -0.0109 | 0.029 | 0.707 | 0.0447 | 1 | Non-significant |
| Type 1 diabetes | ebi-a-GCST90001824 | 0.23 | 0.00237 | 0.00631 | 0.707 | 0.0103 | 1 | Non-significant |
| Psoriasis vulgaris | ebi-a-GCST90001918 | -0.244 | -0.00833 | 0.0222 | 0.707 | 0.0342 | 1 | Non-significant |
| Type 1 diabetes | ebi-a-GCST90002083 | 0.23 | 0.011 | 0.0294 | 0.709 | 0.0478 | 1 | Non-significant |
| Type 1 diabetes | ebi-a-GCST90001841 | 0.23 | -0.00183 | 0.00493 | 0.71 | -0.00798 | 1 | Non-significant |
| Psoriasis vulgaris | ebi-a-GCST90001519 | -0.244 | -0.00922 | 0.0248 | 0.71 | 0.0378 | 1 | Non-significant |
| Type 1 diabetes | ebi-a-GCST90001831 | 0.23 | -0.00574 | 0.0154 | 0.71 | -0.025 | 1 | Non-significant |
| Type 1 diabetes | ebi-a-GCST90002112 | 0.23 | 0.00588 | 0.0159 | 0.711 | 0.0256 | 1 | Non-significant |
| Type 1 diabetes | ebi-a-GCST90001557 | 0.23 | 0.00845 | 0.0228 | 0.711 | 0.0368 | 1 | Non-significant |
| Type 1 diabetes | ebi-a-GCST90002022 | 0.23 | 0.00943 | 0.0258 | 0.715 | 0.041 | 1 | Non-significant |
| Type 1 diabetes | ebi-a-GCST90001842 | 0.23 | -0.00138 | 0.0038 | 0.715 | -0.00602 | 1 | Non-significant |
| Psoriasis vulgaris | ebi-a-GCST90001961 | -0.244 | -0.003 | 0.00822 | 0.715 | 0.0123 | 1 | Non-significant |
| Psoriasis vulgaris | ebi-a-GCST90001992 | -0.244 | -0.00643 | 0.0176 | 0.716 | 0.0264 | 1 | Non-significant |
| Psoriasis vulgaris | ebi-a-GCST90001725 | -0.244 | -0.00254 | 0.00698 | 0.716 | 0.0104 | 1 | Non-significant |
| Type 1 diabetes | ebi-a-GCST90001821 | 0.23 | -0.00246 | 0.00678 | 0.716 | -0.0107 | 1 | Non-significant |
| Psoriasis vulgaris | ebi-a-GCST90002089 | -0.244 | 0.0102 | 0.0281 | 0.717 | -0.0418 | 1 | Non-significant |
| Psoriasis vulgaris | ebi-a-GCST90002084 | -0.244 | 0.0151 | 0.0418 | 0.717 | -0.0621 | 1 | Non-significant |
| Type 1 diabetes | ebi-a-GCST90001714 | 0.23 | -0.0653 | 0.18 | 0.717 | -0.284 | 1 | Non-significant |
| Type 1 diabetes | ebi-a-GCST90001474 | 0.23 | -0.00633 | 0.0175 | 0.718 | -0.0275 | 1 | Non-significant |
| Type 1 diabetes | ebi-a-GCST90001552 | 0.23 | -0.0116 | 0.0323 | 0.719 | -0.0505 | 1 | Non-significant |
| Psoriasis vulgaris | ebi-a-GCST90001688 | -0.244 | -0.00023 | 0.000636 | 0.719 | 0.000937 | 1 | Non-significant |
| Type 1 diabetes | ebi-a-GCST90001544 | 0.23 | -0.00665 | 0.0186 | 0.72 | -0.0289 | 1 | Non-significant |
| Type 1 diabetes | ebi-a-GCST90001664 | 0.23 | -0.00131 | 0.00367 | 0.72 | -0.00572 | 1 | Non-significant |
| Type 1 diabetes | ebi-a-GCST90001825 | 0.23 | -0.00345 | 0.00967 | 0.721 | -0.015 | 1 | Non-significant |
| Psoriasis vulgaris | ebi-a-GCST90002011 | -0.244 | 0.00712 | 0.02 | 0.722 | -0.0292 | 1 | Non-significant |
| Type 1 diabetes | ebi-a-GCST90002052 | 0.23 | -0.00344 | 0.00967 | 0.722 | -0.015 | 1 | Non-significant |
| Psoriasis vulgaris | ebi-a-GCST90001923 | -0.244 | 0.0144 | 0.0407 | 0.723 | -0.0593 | 1 | Non-significant |
| Psoriasis vulgaris | ebi-a-GCST90001712 | -0.244 | 0.0026 | 0.00734 | 0.723 | -0.0107 | 1 | Non-significant |
| Psoriasis vulgaris | ebi-a-GCST90001686 | -0.244 | -0.00879 | 0.0248 | 0.723 | 0.0361 | 1 | Non-significant |
| Type 1 diabetes | ebi-a-GCST90002099 | 0.23 | 0.00111 | 0.00312 | 0.723 | 0.00481 | 1 | Non-significant |
| Psoriasis vulgaris | ebi-a-GCST90001633 | -0.244 | -0.00936 | 0.0265 | 0.724 | 0.0384 | 1 | Non-significant |
| Type 1 diabetes | ebi-a-GCST90001483 | 0.23 | -0.00082 | 0.00233 | 0.724 | -0.00358 | 1 | Non-significant |
| Psoriasis vulgaris | ebi-a-GCST90001917 | -0.244 | 0.00271 | 0.00771 | 0.725 | -0.0111 | 1 | Non-significant |
| Type 1 diabetes | ebi-a-GCST90001535 | 0.23 | -0.0017 | 0.00483 | 0.725 | -0.00739 | 1 | Non-significant |
| Psoriasis vulgaris | ebi-a-GCST90001703 | -0.244 | 0.00255 | 0.00727 | 0.726 | -0.0105 | 1 | Non-significant |
| Type 1 diabetes | ebi-a-GCST90001903 | 0.23 | 0.00717 | 0.0205 | 0.726 | 0.0312 | 1 | Non-significant |
| Psoriasis vulgaris | ebi-a-GCST90001473 | -0.244 | -0.00349 | 0.01 | 0.728 | 0.0143 | 1 | Non-significant |
| Psoriasis vulgaris | ebi-a-GCST90002035 | -0.244 | -0.0053 | 0.0152 | 0.728 | 0.0217 | 1 | Non-significant |
| Psoriasis vulgaris | ebi-a-GCST90002081 | -0.244 | 0.00407 | 0.0117 | 0.728 | -0.0167 | 1 | Non-significant |
| Psoriasis vulgaris | ebi-a-GCST90001856 | -0.244 | 0.00285 | 0.00821 | 0.728 | -0.0117 | 1 | Non-significant |
| Type 1 diabetes | ebi-a-GCST90002019 | 0.23 | 0.00356 | 0.0103 | 0.73 | 0.0155 | 1 | Non-significant |
| Type 1 diabetes | ebi-a-GCST90002007 | 0.23 | 0.00516 | 0.0149 | 0.73 | 0.0224 | 1 | Non-significant |
| Psoriasis vulgaris | ebi-a-GCST90002022 | -0.244 | -0.00696 | 0.0202 | 0.73 | 0.0286 | 1 | Non-significant |
| Psoriasis vulgaris | ebi-a-GCST90001632 | -0.244 | 0.0191 | 0.0553 | 0.73 | -0.0782 | 1 | Non-significant |
| Psoriasis vulgaris | ebi-a-GCST90002012 | -0.244 | -0.00704 | 0.0204 | 0.73 | 0.0289 | 1 | Non-significant |
| Type 1 diabetes | ebi-a-GCST90002110 | 0.23 | -0.00454 | 0.0132 | 0.731 | -0.0198 | 1 | Non-significant |
| Type 1 diabetes | ebi-a-GCST90002032 | 0.23 | 0.000788 | 0.00229 | 0.731 | 0.00343 | 1 | Non-significant |
| Psoriasis vulgaris | ebi-a-GCST90001935 | -0.244 | -0.00883 | 0.0257 | 0.731 | 0.0362 | 1 | Non-significant |
| Psoriasis vulgaris | ebi-a-GCST90001629 | -0.244 | 0.00334 | 0.00974 | 0.731 | -0.0137 | 1 | Non-significant |
| Type 1 diabetes | ebi-a-GCST90001631 | 0.23 | 0.0115 | 0.0337 | 0.732 | 0.0502 | 1 | Non-significant |
| Psoriasis vulgaris | ebi-a-GCST90001794 | -0.244 | 0.00393 | 0.0115 | 0.733 | -0.0161 | 1 | Non-significant |
| Type 1 diabetes | ebi-a-GCST90001913 | 0.23 | 0.00656 | 0.0192 | 0.733 | 0.0285 | 1 | Non-significant |
| Type 1 diabetes | ebi-a-GCST90001493 | 0.23 | 0.000727 | 0.00214 | 0.734 | 0.00316 | 1 | Non-significant |
| Type 1 diabetes | ebi-a-GCST90001650 | 0.23 | 0.0043 | 0.0128 | 0.736 | 0.0187 | 1 | Non-significant |
| Psoriasis vulgaris | ebi-a-GCST90001846 | -0.244 | 0.00295 | 0.00875 | 0.736 | -0.0121 | 1 | Non-significant |
| Type 1 diabetes | ebi-a-GCST90001794 | 0.23 | -0.00622 | 0.0185 | 0.737 | -0.0271 | 1 | Non-significant |
| Type 1 diabetes | ebi-a-GCST90001572 | 0.23 | 0.0162 | 0.0483 | 0.737 | 0.0705 | 1 | Non-significant |
| Psoriasis vulgaris | ebi-a-GCST90001639 | -0.244 | 0.00732 | 0.022 | 0.739 | -0.03 | 1 | Non-significant |
| Psoriasis vulgaris | ebi-a-GCST90002102 | -0.244 | 0.00225 | 0.00676 | 0.74 | -0.00921 | 1 | Non-significant |
| Psoriasis vulgaris | ebi-a-GCST90001821 | -0.244 | -0.00156 | 0.00471 | 0.74 | 0.0064 | 1 | Non-significant |
| Type 1 diabetes | ebi-a-GCST90002046 | 0.23 | 0.00943 | 0.0285 | 0.741 | 0.041 | 1 | Non-significant |
| Type 1 diabetes | ebi-a-GCST90001789 | 0.23 | -0.0114 | 0.0346 | 0.741 | -0.0496 | 1 | Non-significant |
| Type 1 diabetes | ebi-a-GCST90001663 | 0.23 | 0.00308 | 0.00935 | 0.742 | 0.0134 | 1 | Non-significant |
| Type 1 diabetes | ebi-a-GCST90001858 | 0.23 | -0.00123 | 0.00374 | 0.742 | -0.00535 | 1 | Non-significant |
| Psoriasis vulgaris | ebi-a-GCST90001997 | -0.244 | 0.00303 | 0.00922 | 0.742 | -0.0124 | 1 | Non-significant |
| Type 1 diabetes | ebi-a-GCST90001415 | 0.23 | 0.0027 | 0.00823 | 0.743 | 0.0118 | 1 | Non-significant |
| Psoriasis vulgaris | ebi-a-GCST90001557 | -0.244 | -0.0094 | 0.0287 | 0.743 | 0.0386 | 1 | Non-significant |
| Psoriasis vulgaris | ebi-a-GCST90001533 | -0.244 | -0.00178 | 0.00543 | 0.743 | 0.0073 | 1 | Non-significant |
| Type 1 diabetes | ebi-a-GCST90002011 | 0.23 | -0.00656 | 0.02 | 0.744 | -0.0285 | 1 | Non-significant |
| Psoriasis vulgaris | ebi-a-GCST90001938 | -0.244 | -0.0173 | 0.0529 | 0.744 | 0.071 | 1 | Non-significant |
| Psoriasis vulgaris | ebi-a-GCST90001861 | -0.244 | 0.00286 | 0.00876 | 0.744 | -0.0117 | 1 | Non-significant |
| Type 1 diabetes | ebi-a-GCST90001482 | 0.23 | -0.00176 | 0.0054 | 0.744 | -0.00766 | 1 | Non-significant |
| Psoriasis vulgaris | ebi-a-GCST90001472 | -0.244 | -0.00317 | 0.00978 | 0.746 | 0.013 | 1 | Non-significant |
| Type 1 diabetes | ebi-a-GCST90001992 | 0.23 | 0.00434 | 0.0134 | 0.746 | 0.0189 | 1 | Non-significant |
| Psoriasis vulgaris | ebi-a-GCST90001960 | -0.244 | 0.00585 | 0.0181 | 0.747 | -0.024 | 1 | Non-significant |
| Type 1 diabetes | ebi-a-GCST90001785 | 0.23 | 0.0108 | 0.0334 | 0.748 | 0.0468 | 1 | Non-significant |
| Type 1 diabetes | ebi-a-GCST90001909 | 0.23 | 0.00734 | 0.0228 | 0.748 | 0.0319 | 1 | Non-significant |
| Psoriasis vulgaris | ebi-a-GCST90001502 | -0.244 | 0.000677 | 0.00211 | 0.748 | -0.00278 | 1 | Non-significant |
| Type 1 diabetes | ebi-a-GCST90001395 | 0.23 | -0.00698 | 0.0218 | 0.748 | -0.0304 | 1 | Non-significant |
| Psoriasis vulgaris | ebi-a-GCST90001890 | -0.244 | 0.00345 | 0.0108 | 0.748 | -0.0142 | 1 | Non-significant |
| Type 1 diabetes | ebi-a-GCST90001819 | 0.23 | 0.00254 | 0.00794 | 0.749 | 0.0111 | 1 | Non-significant |
| Psoriasis vulgaris | ebi-a-GCST90002060 | -0.244 | -0.00669 | 0.0209 | 0.749 | 0.0274 | 1 | Non-significant |
| Type 1 diabetes | ebi-a-GCST90001492 | 0.23 | 0.00172 | 0.00538 | 0.749 | 0.00749 | 1 | Non-significant |
| Type 1 diabetes | ebi-a-GCST90001942 | 0.23 | 0.00417 | 0.013 | 0.749 | 0.0181 | 1 | Non-significant |
| Psoriasis vulgaris | ebi-a-GCST90001707 | -0.244 | -0.00321 | 0.0101 | 0.75 | 0.0132 | 1 | Non-significant |
| Type 1 diabetes | ebi-a-GCST90001484 | 0.23 | -0.00189 | 0.00595 | 0.75 | -0.00824 | 1 | Non-significant |
| Type 1 diabetes | ebi-a-GCST90001503 | 0.23 | 0.00119 | 0.00375 | 0.75 | 0.0052 | 1 | Non-significant |
| Psoriasis vulgaris | ebi-a-GCST90001492 | -0.244 | 0.00177 | 0.00557 | 0.75 | -0.00727 | 1 | Non-significant |
| Type 1 diabetes | ebi-a-GCST90001499 | 0.23 | 0.000653 | 0.00206 | 0.751 | 0.00284 | 1 | Non-significant |
| Psoriasis vulgaris | ebi-a-GCST90001482 | -0.244 | -0.0014 | 0.00443 | 0.751 | 0.00575 | 1 | Non-significant |
| Type 1 diabetes | ebi-a-GCST90001627 | 0.23 | 0.00712 | 0.0225 | 0.752 | 0.031 | 1 | Non-significant |
| Psoriasis vulgaris | ebi-a-GCST90001548 | -0.244 | -0.00596 | 0.0189 | 0.752 | 0.0245 | 1 | Non-significant |
| Psoriasis vulgaris | ebi-a-GCST90002113 | -0.244 | -0.00826 | 0.0261 | 0.752 | 0.0339 | 1 | Non-significant |
| Type 1 diabetes | ebi-a-GCST90001760 | 0.23 | 0.00788 | 0.025 | 0.752 | 0.0343 | 1 | Non-significant |
| Psoriasis vulgaris | ebi-a-GCST90001753 | -0.244 | 0.00844 | 0.0268 | 0.753 | -0.0346 | 1 | Non-significant |
| Psoriasis vulgaris | ebi-a-GCST90001469 | -0.244 | 0.00251 | 0.00798 | 0.753 | -0.0103 | 1 | Non-significant |
| Type 1 diabetes | ebi-a-GCST90001696 | 0.23 | 0.000423 | 0.00135 | 0.753 | 0.00184 | 1 | Non-significant |
| Type 1 diabetes | ebi-a-GCST90001461 | 0.23 | 0.00322 | 0.0103 | 0.754 | 0.014 | 1 | Non-significant |
| Type 1 diabetes | ebi-a-GCST90002114 | 0.23 | 0.00698 | 0.0222 | 0.754 | 0.0304 | 1 | Non-significant |
| Type 1 diabetes | ebi-a-GCST90001519 | 0.23 | -0.00327 | 0.0104 | 0.754 | -0.0142 | 1 | Non-significant |
| Psoriasis vulgaris | ebi-a-GCST90001647 | -0.244 | 0.00222 | 0.00707 | 0.754 | -0.0091 | 1 | Non-significant |
| Type 1 diabetes | ebi-a-GCST90001501 | 0.23 | 0.00176 | 0.00562 | 0.754 | 0.00765 | 1 | Non-significant |
| Psoriasis vulgaris | ebi-a-GCST90001468 | -0.244 | 0.00336 | 0.0108 | 0.755 | -0.0138 | 1 | Non-significant |
| Type 1 diabetes | ebi-a-GCST90001768 | 0.23 | 0.0053 | 0.017 | 0.755 | 0.0231 | 1 | Non-significant |
| Type 1 diabetes | ebi-a-GCST90002001 | 0.23 | 0.00513 | 0.0165 | 0.755 | 0.0223 | 1 | Non-significant |
| Psoriasis vulgaris | ebi-a-GCST90001508 | -0.244 | 0.00267 | 0.00856 | 0.755 | -0.0109 | 1 | Non-significant |
| Psoriasis vulgaris | ebi-a-GCST90001494 | -0.244 | 0.00146 | 0.00469 | 0.756 | -0.00599 | 1 | Non-significant |
| Psoriasis vulgaris | ebi-a-GCST90001774 | -0.244 | -0.00469 | 0.0151 | 0.756 | 0.0192 | 1 | Non-significant |
| Type 1 diabetes | ebi-a-GCST90001941 | 0.23 | 0.00496 | 0.016 | 0.756 | 0.0216 | 1 | Non-significant |
| Type 1 diabetes | ebi-a-GCST90001923 | 0.23 | 0.00499 | 0.0161 | 0.757 | 0.0217 | 1 | Non-significant |
| Type 1 diabetes | ebi-a-GCST90001581 | 0.23 | 0.00221 | 0.00715 | 0.757 | 0.00963 | 1 | Non-significant |
| Type 1 diabetes | ebi-a-GCST90001573 | 0.23 | 0.00278 | 0.00902 | 0.758 | 0.0121 | 1 | Non-significant |
| Psoriasis vulgaris | ebi-a-GCST90001524 | -0.244 | -0.00298 | 0.00966 | 0.758 | 0.0122 | 1 | Non-significant |
| Type 1 diabetes | ebi-a-GCST90002003 | 0.23 | -0.00282 | 0.00915 | 0.758 | -0.0123 | 1 | Non-significant |
| Psoriasis vulgaris | ebi-a-GCST90001839 | -0.244 | 0.00495 | 0.0161 | 0.758 | -0.0203 | 1 | Non-significant |
| Type 1 diabetes | ebi-a-GCST90001459 | 0.23 | 0.00395 | 0.0129 | 0.76 | 0.0172 | 1 | Non-significant |
| Type 1 diabetes | ebi-a-GCST90002031 | 0.23 | -0.00099 | 0.00323 | 0.76 | -0.00429 | 1 | Non-significant |
| Psoriasis vulgaris | ebi-a-GCST90002114 | -0.244 | -0.00674 | 0.0221 | 0.761 | 0.0277 | 1 | Non-significant |
| Psoriasis vulgaris | ebi-a-GCST90001827 | -0.244 | 0.00178 | 0.00589 | 0.762 | -0.00732 | 1 | Non-significant |
| Psoriasis vulgaris | ebi-a-GCST90001711 | -0.244 | 0.00257 | 0.0085 | 0.762 | -0.0105 | 1 | Non-significant |
| Type 1 diabetes | ebi-a-GCST90001494 | 0.23 | -0.00118 | 0.0039 | 0.762 | -0.00513 | 1 | Non-significant |
| Psoriasis vulgaris | ebi-a-GCST90002019 | -0.244 | 0.00332 | 0.011 | 0.763 | -0.0136 | 1 | Non-significant |
| Psoriasis vulgaris | ebi-a-GCST90001664 | -0.244 | 0.00167 | 0.00556 | 0.763 | -0.00687 | 1 | Non-significant |
| Psoriasis vulgaris | ebi-a-GCST90001406 | -0.244 | -0.0171 | 0.057 | 0.764 | 0.0703 | 1 | Non-significant |
| Type 1 diabetes | ebi-a-GCST90001946 | 0.23 | -0.00117 | 0.00389 | 0.764 | -0.00508 | 1 | Non-significant |
| Type 1 diabetes | ebi-a-GCST90001967 | 0.23 | 0.00997 | 0.0332 | 0.764 | 0.0434 | 1 | Non-significant |
| Psoriasis vulgaris | ebi-a-GCST90001408 | -0.244 | -0.017 | 0.0566 | 0.764 | 0.0697 | 1 | Non-significant |
| Psoriasis vulgaris | ebi-a-GCST90001705 | -0.244 | 0.00245 | 0.00815 | 0.764 | -0.01 | 1 | Non-significant |
| Psoriasis vulgaris | ebi-a-GCST90001511 | -0.244 | -0.00315 | 0.0105 | 0.764 | 0.0129 | 1 | Non-significant |
| Type 1 diabetes | ebi-a-GCST90002094 | 0.23 | -0.00497 | 0.0166 | 0.765 | -0.0216 | 1 | Non-significant |
| Psoriasis vulgaris | ebi-a-GCST90001786 | -0.244 | -0.00499 | 0.0167 | 0.765 | 0.0205 | 1 | Non-significant |
| Psoriasis vulgaris | ebi-a-GCST90001868 | -0.244 | -0.0161 | 0.0542 | 0.766 | 0.0661 | 1 | Non-significant |
| Type 1 diabetes | ebi-a-GCST90002000 | 0.23 | 0.0153 | 0.0516 | 0.767 | 0.0665 | 1 | Non-significant |
| Psoriasis vulgaris | ebi-a-GCST90001395 | -0.244 | 0.00603 | 0.0204 | 0.768 | -0.0248 | 1 | Non-significant |
| Psoriasis vulgaris | ebi-a-GCST90001392 | -0.244 | -0.0268 | 0.0909 | 0.768 | 0.11 | 1 | Non-significant |
| Type 1 diabetes | ebi-a-GCST90001955 | 0.23 | -0.00068 | 0.00232 | 0.769 | -0.00297 | 1 | Non-significant |
| Type 1 diabetes | ebi-a-GCST90001969 | 0.23 | 0.00327 | 0.0112 | 0.77 | 0.0142 | 1 | Non-significant |
| Psoriasis vulgaris | ebi-a-GCST90001897 | -0.244 | -0.00123 | 0.00423 | 0.771 | 0.00506 | 1 | Non-significant |
| Type 1 diabetes | ebi-a-GCST90001589 | 0.23 | 0.00812 | 0.0279 | 0.771 | 0.0353 | 1 | Non-significant |
| Psoriasis vulgaris | ebi-a-GCST90001844 | -0.244 | -0.005 | 0.0172 | 0.771 | 0.0205 | 1 | Non-significant |
| Type 1 diabetes | ebi-a-GCST90001998 | 0.23 | 0.0106 | 0.0365 | 0.771 | 0.0461 | 1 | Non-significant |
| Type 1 diabetes | ebi-a-GCST90001406 | 0.23 | 0.00815 | 0.0282 | 0.773 | 0.0355 | 1 | Non-significant |
| Psoriasis vulgaris | ebi-a-GCST90001867 | -0.244 | -0.00367 | 0.0128 | 0.774 | 0.015 | 1 | Non-significant |
| Type 1 diabetes | ebi-a-GCST90001976 | 0.23 | 0.00614 | 0.0214 | 0.774 | 0.0267 | 1 | Non-significant |
| Type 1 diabetes | ebi-a-GCST90001699 | 0.23 | 0.00105 | 0.00368 | 0.775 | 0.00457 | 1 | Non-significant |
| Type 1 diabetes | ebi-a-GCST90001561 | 0.23 | 0.000536 | 0.00189 | 0.777 | 0.00233 | 1 | Non-significant |
| Psoriasis vulgaris | ebi-a-GCST90002002 | -0.244 | -0.00624 | 0.0221 | 0.778 | 0.0256 | 1 | Non-significant |
| Psoriasis vulgaris | ebi-a-GCST90001706 | -0.244 | -0.00292 | 0.0104 | 0.778 | 0.012 | 1 | Non-significant |
| Type 1 diabetes | ebi-a-GCST90001408 | 0.23 | 0.00706 | 0.0251 | 0.779 | 0.0307 | 1 | Non-significant |
| Type 1 diabetes | ebi-a-GCST90001783 | 0.23 | 0.00268 | 0.00954 | 0.779 | 0.0116 | 1 | Non-significant |
| Type 1 diabetes | ebi-a-GCST90002113 | 0.23 | 0.00202 | 0.0072 | 0.78 | 0.00877 | 1 | Non-significant |
| Psoriasis vulgaris | ebi-a-GCST90001704 | -0.244 | 0.00267 | 0.00954 | 0.78 | -0.011 | 1 | Non-significant |
| Type 1 diabetes | ebi-a-GCST90001638 | 0.23 | 0.00154 | 0.00552 | 0.78 | 0.00671 | 1 | Non-significant |
| Type 1 diabetes | ebi-a-GCST90001497 | 0.23 | -0.00112 | 0.004 | 0.78 | -0.00486 | 1 | Non-significant |
| Type 1 diabetes | ebi-a-GCST90001774 | 0.23 | 0.00327 | 0.0117 | 0.781 | 0.0142 | 1 | Non-significant |
| Type 1 diabetes | ebi-a-GCST90001541 | 0.23 | 0.00526 | 0.0189 | 0.781 | 0.0229 | 1 | Non-significant |
| Psoriasis vulgaris | ebi-a-GCST90002120 | -0.244 | 0.00329 | 0.0119 | 0.781 | -0.0135 | 1 | Non-significant |
| Type 1 diabetes | ebi-a-GCST90001556 | 0.23 | -0.00242 | 0.00873 | 0.782 | -0.0105 | 1 | Non-significant |
| Type 1 diabetes | ebi-a-GCST90001570 | 0.23 | 0.0017 | 0.00613 | 0.782 | 0.00739 | 1 | Non-significant |
| Psoriasis vulgaris | ebi-a-GCST90002058 | -0.244 | -0.00316 | 0.0114 | 0.783 | 0.0129 | 1 | Non-significant |
| Type 1 diabetes | ebi-a-GCST90001641 | 0.23 | 0.00354 | 0.0129 | 0.783 | 0.0154 | 1 | Non-significant |
| Psoriasis vulgaris | ebi-a-GCST90001501 | -0.244 | 0.000959 | 0.0035 | 0.784 | -0.00393 | 1 | Non-significant |
| Type 1 diabetes | ebi-a-GCST90001489 | 0.23 | 0.00132 | 0.00483 | 0.785 | 0.00574 | 1 | Non-significant |
| Type 1 diabetes | ebi-a-GCST90001775 | 0.23 | -0.00461 | 0.0169 | 0.785 | -0.0201 | 1 | Non-significant |
| Psoriasis vulgaris | ebi-a-GCST90001775 | -0.244 | 0.00243 | 0.00892 | 0.786 | -0.00995 | 1 | Non-significant |
| Type 1 diabetes | ebi-a-GCST90001769 | 0.23 | 0.00538 | 0.0199 | 0.787 | 0.0234 | 1 | Non-significant |
| Psoriasis vulgaris | ebi-a-GCST90001627 | -0.244 | 0.00249 | 0.00922 | 0.787 | -0.0102 | 1 | Non-significant |
| Psoriasis vulgaris | ebi-a-GCST90001495 | -0.244 | -0.00052 | 0.00193 | 0.788 | 0.00213 | 1 | Non-significant |
| Type 1 diabetes | ebi-a-GCST90001795 | 0.23 | 0.00274 | 0.0102 | 0.789 | 0.0119 | 1 | Non-significant |
| Psoriasis vulgaris | ebi-a-GCST90001418 | -0.244 | -0.0319 | 0.119 | 0.789 | 0.131 | 1 | Non-significant |
| Type 1 diabetes | ebi-a-GCST90001392 | 0.23 | -0.00478 | 0.0179 | 0.789 | -0.0208 | 1 | Non-significant |
| Psoriasis vulgaris | ebi-a-GCST90001612 | -0.244 | -0.0172 | 0.0644 | 0.789 | 0.0706 | 1 | Non-significant |
| Type 1 diabetes | ebi-a-GCST90001446 | 0.23 | -0.00212 | 0.00793 | 0.789 | -0.00922 | 1 | Non-significant |
| Psoriasis vulgaris | ebi-a-GCST90001746 | -0.244 | -0.00586 | 0.022 | 0.79 | 0.024 | 1 | Non-significant |
| Psoriasis vulgaris | ebi-a-GCST90001446 | -0.244 | 0.00273 | 0.0103 | 0.79 | -0.0112 | 1 | Non-significant |
| Psoriasis vulgaris | ebi-a-GCST90001631 | -0.244 | 0.00721 | 0.0271 | 0.79 | -0.0296 | 1 | Non-significant |
| Type 1 diabetes | ebi-a-GCST90001746 | 0.23 | -0.0069 | 0.0259 | 0.79 | -0.03 | 1 | Non-significant |
| Type 1 diabetes | ebi-a-GCST90001524 | 0.23 | -0.00174 | 0.00655 | 0.791 | -0.00755 | 1 | Non-significant |
| Type 1 diabetes | ebi-a-GCST90001434 | 0.23 | 0.00368 | 0.014 | 0.792 | 0.016 | 1 | Non-significant |
| Psoriasis vulgaris | ebi-a-GCST90002090 | -0.244 | 0.00506 | 0.0193 | 0.793 | -0.0208 | 1 | Non-significant |
| Psoriasis vulgaris | ebi-a-GCST90001699 | -0.244 | -0.00082 | 0.00314 | 0.794 | 0.00337 | 1 | Non-significant |
| Psoriasis vulgaris | ebi-a-GCST90001630 | -0.244 | -0.00684 | 0.0262 | 0.794 | 0.028 | 1 | Non-significant |
| Type 1 diabetes | ebi-a-GCST90001791 | 0.23 | -0.00296 | 0.0114 | 0.795 | -0.0129 | 1 | Non-significant |
| Type 1 diabetes | ebi-a-GCST90001829 | 0.23 | -0.00122 | 0.00473 | 0.796 | -0.00532 | 1 | Non-significant |
| Psoriasis vulgaris | ebi-a-GCST90001431 | -0.244 | 0.0199 | 0.0772 | 0.796 | -0.0818 | 1 | Non-significant |
| Psoriasis vulgaris | ebi-a-GCST90001603 | -0.244 | 0.0049 | 0.019 | 0.796 | -0.0201 | 1 | Non-significant |
| Psoriasis vulgaris | ebi-a-GCST90001717 | -0.244 | 0.0013 | 0.00506 | 0.797 | -0.00535 | 1 | Non-significant |
| Psoriasis vulgaris | ebi-a-GCST90001608 | -0.244 | -0.0037 | 0.0144 | 0.797 | 0.0152 | 1 | Non-significant |
| Type 1 diabetes | ebi-a-GCST90001766 | 0.23 | 0.00322 | 0.0125 | 0.797 | 0.014 | 1 | Non-significant |
| Psoriasis vulgaris | ebi-a-GCST90001597 | -0.244 | -0.0158 | 0.0618 | 0.798 | 0.0649 | 1 | Non-significant |
| Psoriasis vulgaris | ebi-a-GCST90001769 | -0.244 | -0.00245 | 0.00957 | 0.798 | 0.01 | 1 | Non-significant |
| Psoriasis vulgaris | ebi-a-GCST90001953 | -0.244 | -0.00075 | 0.00291 | 0.798 | 0.00305 | 1 | Non-significant |
| Psoriasis vulgaris | ebi-a-GCST90001620 | -0.244 | -0.00232 | 0.00907 | 0.798 | 0.00952 | 1 | Non-significant |
| Psoriasis vulgaris | ebi-a-GCST90001479 | -0.244 | -0.00846 | 0.0332 | 0.799 | 0.0347 | 1 | Non-significant |
| Type 1 diabetes | ebi-a-GCST90001718 | 0.23 | 0.000911 | 0.00358 | 0.799 | 0.00397 | 1 | Non-significant |
| Type 1 diabetes | ebi-a-GCST90002038 | 0.23 | -0.00587 | 0.0231 | 0.8 | -0.0255 | 1 | Non-significant |
| Psoriasis vulgaris | ebi-a-GCST90001404 | -0.244 | 0.00918 | 0.0362 | 0.8 | -0.0377 | 1 | Non-significant |
| Psoriasis vulgaris | ebi-a-GCST90001946 | -0.244 | -0.00061 | 0.00242 | 0.8 | 0.00251 | 1 | Non-significant |
| Type 1 diabetes | ebi-a-GCST90001691 | 0.23 | -0.00192 | 0.00761 | 0.801 | -0.00836 | 1 | Non-significant |
| Psoriasis vulgaris | ebi-a-GCST90001852 | -0.244 | -0.00368 | 0.0146 | 0.801 | 0.0151 | 1 | Non-significant |
| Psoriasis vulgaris | ebi-a-GCST90001832 | -0.244 | 0.00496 | 0.0198 | 0.802 | -0.0203 | 1 | Non-significant |
| Psoriasis vulgaris | ebi-a-GCST90001783 | -0.244 | -0.00147 | 0.00585 | 0.802 | 0.00602 | 1 | Non-significant |
| Psoriasis vulgaris | ebi-a-GCST90002003 | -0.244 | 0.00223 | 0.00893 | 0.803 | -0.00916 | 1 | Non-significant |
| Type 1 diabetes | ebi-a-GCST90002037 | 0.23 | -0.00607 | 0.0243 | 0.803 | -0.0264 | 1 | Non-significant |
| Psoriasis vulgaris | ebi-a-GCST90001601 | -0.244 | 0.00434 | 0.0174 | 0.803 | -0.0178 | 1 | Non-significant |
| Type 1 diabetes | ebi-a-GCST90002071 | 0.23 | 0.00596 | 0.0239 | 0.803 | 0.0259 | 1 | Non-significant |
| Psoriasis vulgaris | ebi-a-GCST90001740 | -0.244 | 0.0114 | 0.0459 | 0.804 | -0.0468 | 1 | Non-significant |
| Psoriasis vulgaris | ebi-a-GCST90001795 | -0.244 | -0.00202 | 0.00818 | 0.805 | 0.0083 | 1 | Non-significant |
| Psoriasis vulgaris | ebi-a-GCST90001613 | -0.244 | -0.00694 | 0.0281 | 0.805 | 0.0285 | 1 | Non-significant |
| Psoriasis vulgaris | ebi-a-GCST90001424 | -0.244 | -0.0287 | 0.116 | 0.805 | 0.118 | 1 | Non-significant |
| Psoriasis vulgaris | ebi-a-GCST90001573 | -0.244 | -0.00173 | 0.00701 | 0.805 | 0.00708 | 1 | Non-significant |
| Type 1 diabetes | ebi-a-GCST90001710 | 0.23 | 0.00112 | 0.00454 | 0.805 | 0.00487 | 1 | Non-significant |
| Psoriasis vulgaris | ebi-a-GCST90001973 | -0.244 | 0.00466 | 0.019 | 0.806 | -0.0191 | 1 | Non-significant |
| Type 1 diabetes | ebi-a-GCST90001945 | 0.23 | -0.00599 | 0.0245 | 0.807 | -0.0261 | 1 | Non-significant |
| Psoriasis vulgaris | ebi-a-GCST90001604 | -0.244 | -0.00255 | 0.0104 | 0.807 | 0.0105 | 1 | Non-significant |
| Psoriasis vulgaris | ebi-a-GCST90001878 | -0.244 | 0.00428 | 0.0175 | 0.807 | -0.0176 | 1 | Non-significant |
| Type 1 diabetes | ebi-a-GCST90001424 | 0.23 | -0.0145 | 0.0595 | 0.807 | -0.0633 | 1 | Non-significant |
| Type 1 diabetes | ebi-a-GCST90001805 | 0.23 | -0.0012 | 0.00494 | 0.808 | -0.00523 | 1 | Non-significant |
| Type 1 diabetes | ebi-a-GCST90001636 | 0.23 | -0.0194 | 0.0799 | 0.808 | -0.0844 | 1 | Non-significant |
| Psoriasis vulgaris | ebi-a-GCST90001842 | -0.244 | -0.00097 | 0.00399 | 0.808 | 0.00397 | 1 | Non-significant |
| Psoriasis vulgaris | ebi-a-GCST90001822 | -0.244 | 0.002 | 0.0083 | 0.81 | -0.00821 | 1 | Non-significant |
| Type 1 diabetes | ebi-a-GCST90001944 | 0.23 | -0.00575 | 0.0239 | 0.81 | -0.025 | 1 | Non-significant |
| Psoriasis vulgaris | ebi-a-GCST90002025 | -0.244 | -0.00279 | 0.0117 | 0.811 | 0.0114 | 1 | Non-significant |
| Type 1 diabetes | ebi-a-GCST90001999 | 0.23 | -0.00424 | 0.0177 | 0.811 | -0.0184 | 1 | Non-significant |
| Psoriasis vulgaris | ebi-a-GCST90001687 | -0.244 | -0.00456 | 0.0192 | 0.813 | 0.0187 | 1 | Non-significant |
| Type 1 diabetes | ebi-a-GCST90002098 | 0.23 | -0.00044 | 0.00187 | 0.813 | -0.00192 | 1 | Non-significant |
| Psoriasis vulgaris | ebi-a-GCST90001589 | -0.244 | 0.00634 | 0.0272 | 0.815 | -0.026 | 1 | Non-significant |
| Psoriasis vulgaris | ebi-a-GCST90001980 | -0.244 | -0.00216 | 0.00925 | 0.816 | 0.00885 | 1 | Non-significant |
| Type 1 diabetes | ebi-a-GCST90001878 | 0.23 | -0.00201 | 0.00865 | 0.816 | -0.00877 | 1 | Non-significant |
| Psoriasis vulgaris | ebi-a-GCST90001993 | -0.244 | 0.0125 | 0.0539 | 0.817 | -0.0512 | 1 | Non-significant |
| Type 1 diabetes | ebi-a-GCST90002095 | 0.23 | 0.00213 | 0.00921 | 0.817 | 0.00928 | 1 | Non-significant |
| Type 1 diabetes | ebi-a-GCST90001887 | 0.23 | -0.00147 | 0.00637 | 0.817 | -0.00641 | 1 | Non-significant |
| Type 1 diabetes | ebi-a-GCST90001442 | 0.23 | 0.00787 | 0.034 | 0.817 | 0.0342 | 1 | Non-significant |
| Type 1 diabetes | ebi-a-GCST90001961 | 0.23 | -0.00168 | 0.00728 | 0.817 | -0.00732 | 1 | Non-significant |
| Psoriasis vulgaris | ebi-a-GCST90001523 | -0.244 | 0.00954 | 0.0413 | 0.817 | -0.0391 | 1 | Non-significant |
| Psoriasis vulgaris | ebi-a-GCST90001713 | -0.244 | 0.00257 | 0.0112 | 0.818 | -0.0105 | 1 | Non-significant |
| Psoriasis vulgaris | ebi-a-GCST90001970 | -0.244 | 0.0056 | 0.0244 | 0.818 | -0.023 | 1 | Non-significant |
| Psoriasis vulgaris | ebi-a-GCST90001556 | -0.244 | -0.00361 | 0.0158 | 0.819 | 0.0148 | 1 | Non-significant |
| Psoriasis vulgaris | ebi-a-GCST90001442 | -0.244 | -0.007 | 0.0305 | 0.819 | 0.0287 | 1 | Non-significant |
| Psoriasis vulgaris | ebi-a-GCST90001609 | -0.244 | -0.00282 | 0.0123 | 0.819 | 0.0116 | 1 | Non-significant |
| Type 1 diabetes | ebi-a-GCST90001736 | 0.23 | -0.00171 | 0.00747 | 0.819 | -0.00742 | 1 | Non-significant |
| Psoriasis vulgaris | ebi-a-GCST90002046 | -0.244 | 0.00248 | 0.0109 | 0.819 | -0.0102 | 1 | Non-significant |
| Type 1 diabetes | ebi-a-GCST90001890 | 0.23 | -0.00211 | 0.00928 | 0.82 | -0.00918 | 1 | Non-significant |
| Psoriasis vulgaris | ebi-a-GCST90001986 | -0.244 | -0.00331 | 0.0147 | 0.822 | 0.0136 | 1 | Non-significant |
| Type 1 diabetes | ebi-a-GCST90002029 | 0.23 | -0.00219 | 0.00977 | 0.823 | -0.00953 | 1 | Non-significant |
| Psoriasis vulgaris | ebi-a-GCST90001505 | -0.244 | -0.00182 | 0.00814 | 0.823 | 0.00748 | 1 | Non-significant |
| Psoriasis vulgaris | ebi-a-GCST90001575 | -0.244 | -0.0059 | 0.0264 | 0.823 | 0.0242 | 1 | Non-significant |
| Type 1 diabetes | ebi-a-GCST90001686 | 0.23 | 0.00371 | 0.0167 | 0.824 | 0.0162 | 1 | Non-significant |
| Psoriasis vulgaris | ebi-a-GCST90001995 | -0.244 | 0.00213 | 0.0096 | 0.825 | -0.00872 | 1 | Non-significant |
| Psoriasis vulgaris | ebi-a-GCST90001892 | -0.244 | -0.00088 | 0.00397 | 0.825 | 0.0036 | 1 | Non-significant |
| Psoriasis vulgaris | ebi-a-GCST90001766 | -0.244 | -0.00125 | 0.00574 | 0.827 | 0.00515 | 1 | Non-significant |
| Type 1 diabetes | ebi-a-GCST90001516 | 0.23 | -0.00082 | 0.00374 | 0.827 | -0.00355 | 1 | Non-significant |
| Psoriasis vulgaris | ebi-a-GCST90001736 | -0.244 | 0.00139 | 0.00637 | 0.827 | -0.0057 | 1 | Non-significant |
| Type 1 diabetes | ebi-a-GCST90001877 | 0.23 | 0.00268 | 0.0123 | 0.828 | 0.0117 | 1 | Non-significant |
| Type 1 diabetes | ebi-a-GCST90001818 | 0.23 | -0.00584 | 0.0269 | 0.828 | -0.0254 | 1 | Non-significant |
| Type 1 diabetes | ebi-a-GCST90001505 | 0.23 | -0.00152 | 0.00705 | 0.829 | -0.0066 | 1 | Non-significant |
| Psoriasis vulgaris | ebi-a-GCST90001767 | -0.244 | -0.00178 | 0.00843 | 0.833 | 0.00731 | 1 | Non-significant |
| Type 1 diabetes | ebi-a-GCST90001715 | 0.23 | 0.000573 | 0.00274 | 0.834 | 0.00249 | 1 | Non-significant |
| Psoriasis vulgaris | ebi-a-GCST90001987 | -0.244 | -0.00353 | 0.017 | 0.836 | 0.0145 | 1 | Non-significant |
| Type 1 diabetes | ebi-a-GCST90002014 | 0.23 | 0.00431 | 0.0208 | 0.836 | 0.0187 | 1 | Non-significant |
| Type 1 diabetes | ebi-a-GCST90001476 | 0.23 | -0.00701 | 0.034 | 0.837 | -0.0305 | 1 | Non-significant |
| Type 1 diabetes | ebi-a-GCST90001816 | 0.23 | 0.00273 | 0.0132 | 0.837 | 0.0119 | 1 | Non-significant |
| Psoriasis vulgaris | ebi-a-GCST90001877 | -0.244 | 0.00209 | 0.0102 | 0.837 | -0.00857 | 1 | Non-significant |
| Type 1 diabetes | ebi-a-GCST90001772 | 0.23 | 0.00217 | 0.0106 | 0.838 | 0.00944 | 1 | Non-significant |
| Psoriasis vulgaris | ebi-a-GCST90002079 | -0.244 | 0.00809 | 0.0396 | 0.838 | -0.0332 | 1 | Non-significant |
| Psoriasis vulgaris | ebi-a-GCST90001955 | -0.244 | -0.00046 | 0.00226 | 0.839 | 0.00188 | 1 | Non-significant |
| Psoriasis vulgaris | ebi-a-GCST90001999 | -0.244 | 0.00337 | 0.0167 | 0.84 | -0.0138 | 1 | Non-significant |
| Type 1 diabetes | ebi-a-GCST90001510 | 0.23 | -0.00291 | 0.0144 | 0.84 | -0.0127 | 1 | Non-significant |
| Psoriasis vulgaris | ebi-a-GCST90001850 | -0.244 | 0.00437 | 0.0217 | 0.841 | -0.0179 | 1 | Non-significant |
| Psoriasis vulgaris | ebi-a-GCST90001618 | -0.244 | -0.00093 | 0.00463 | 0.841 | 0.00381 | 1 | Non-significant |
| Psoriasis vulgaris | ebi-a-GCST90001994 | -0.244 | 0.00148 | 0.00738 | 0.841 | -0.00607 | 1 | Non-significant |
| Psoriasis vulgaris | ebi-a-GCST90001562 | -0.244 | 0.00321 | 0.016 | 0.842 | -0.0131 | 1 | Non-significant |
| Psoriasis vulgaris | ebi-a-GCST90001869 | -0.244 | -0.00194 | 0.00971 | 0.842 | 0.00794 | 1 | Non-significant |
| Type 1 diabetes | ebi-a-GCST90001645 | 0.23 | -0.00235 | 0.0118 | 0.843 | -0.0102 | 1 | Non-significant |
| Psoriasis vulgaris | ebi-a-GCST90001496 | -0.244 | -0.00092 | 0.00467 | 0.843 | 0.00379 | 1 | Non-significant |
| Psoriasis vulgaris | ebi-a-GCST90001926 | -0.244 | 0.00138 | 0.00699 | 0.843 | -0.00566 | 1 | Non-significant |
| Type 1 diabetes | ebi-a-GCST90001481 | 0.23 | -0.00039 | 0.00198 | 0.843 | -0.00171 | 1 | Non-significant |
| Psoriasis vulgaris | ebi-a-GCST90001571 | -0.244 | -0.0045 | 0.0228 | 0.844 | 0.0184 | 1 | Non-significant |
| Type 1 diabetes | ebi-a-GCST90001767 | 0.23 | 0.000851 | 0.00433 | 0.844 | 0.0037 | 1 | Non-significant |
| Psoriasis vulgaris | ebi-a-GCST90001401 | -0.244 | 0.00338 | 0.0172 | 0.844 | -0.0139 | 1 | Non-significant |
| Psoriasis vulgaris | ebi-a-GCST90001696 | -0.244 | 0.000329 | 0.00169 | 0.845 | -0.00135 | 1 | Non-significant |
| Type 1 diabetes | ebi-a-GCST90001571 | 0.23 | -0.00197 | 0.0101 | 0.846 | -0.00857 | 1 | Non-significant |
| Psoriasis vulgaris | ebi-a-GCST90001478 | -0.244 | 0.00936 | 0.0482 | 0.846 | -0.0384 | 1 | Non-significant |
| Type 1 diabetes | ebi-a-GCST90002073 | 0.23 | 0.000632 | 0.00326 | 0.846 | 0.00275 | 1 | Non-significant |
| Psoriasis vulgaris | ebi-a-GCST90001901 | -0.244 | 0.00341 | 0.0176 | 0.846 | -0.014 | 1 | Non-significant |
| Type 1 diabetes | ebi-a-GCST90001800 | 0.23 | -0.0018 | 0.0093 | 0.847 | -0.00783 | 1 | Non-significant |
| Psoriasis vulgaris | ebi-a-GCST90001942 | -0.244 | 0.00199 | 0.0103 | 0.847 | -0.00818 | 1 | Non-significant |
| Type 1 diabetes | ebi-a-GCST90001953 | 0.23 | -0.0004 | 0.0021 | 0.847 | -0.00176 | 1 | Non-significant |
| Psoriasis vulgaris | ebi-a-GCST90001504 | -0.244 | 0.00175 | 0.00914 | 0.848 | -0.0072 | 1 | Non-significant |
| Type 1 diabetes | ebi-a-GCST90001938 | 0.23 | 0.00494 | 0.0259 | 0.849 | 0.0215 | 1 | Non-significant |
| Psoriasis vulgaris | ebi-a-GCST90001916 | -0.244 | -0.00241 | 0.0127 | 0.849 | 0.00989 | 1 | Non-significant |
| Type 1 diabetes | ebi-a-GCST90001830 | 0.23 | -0.00307 | 0.0162 | 0.85 | -0.0134 | 1 | Non-significant |
| Psoriasis vulgaris | ebi-a-GCST90001434 | -0.244 | 0.00133 | 0.00702 | 0.85 | -0.00544 | 1 | Non-significant |
| Psoriasis vulgaris | ebi-a-GCST90002017 | -0.244 | 0.00486 | 0.0257 | 0.85 | -0.0199 | 1 | Non-significant |
| Psoriasis vulgaris | ebi-a-GCST90001481 | -0.244 | -0.00034 | 0.0018 | 0.85 | 0.00139 | 1 | Non-significant |
| Psoriasis vulgaris | ebi-a-GCST90002062 | -0.244 | -0.00831 | 0.0443 | 0.851 | 0.0341 | 1 | Non-significant |
| Type 1 diabetes | ebi-a-GCST90001765 | 0.23 | 0.000802 | 0.0043 | 0.852 | 0.00349 | 1 | Non-significant |
| Psoriasis vulgaris | ebi-a-GCST90001830 | -0.244 | -0.00525 | 0.0282 | 0.852 | 0.0215 | 1 | Non-significant |
| Type 1 diabetes | ebi-a-GCST90001496 | 0.23 | -0.00033 | 0.0018 | 0.853 | -0.00145 | 1 | Non-significant |
| Type 1 diabetes | ebi-a-GCST90001491 | 0.23 | -0.00084 | 0.00455 | 0.854 | -0.00364 | 1 | Non-significant |
| Type 1 diabetes | ebi-a-GCST90001475 | 0.23 | 0.00401 | 0.0219 | 0.855 | 0.0174 | 1 | Non-significant |
| Psoriasis vulgaris | ebi-a-GCST90001594 | -0.244 | 0.00331 | 0.0181 | 0.855 | -0.0136 | 1 | Non-significant |
| Type 1 diabetes | ebi-a-GCST90001997 | 0.23 | 0.00137 | 0.00754 | 0.855 | 0.00598 | 1 | Non-significant |
| Type 1 diabetes | ebi-a-GCST90001988 | 0.23 | 0.00615 | 0.0339 | 0.856 | 0.0267 | 1 | Non-significant |
| Type 1 diabetes | ebi-a-GCST90001575 | 0.23 | -0.00213 | 0.0117 | 0.856 | -0.00925 | 1 | Non-significant |
| Type 1 diabetes | ebi-a-GCST90001507 | 0.23 | 0.00355 | 0.0196 | 0.856 | 0.0155 | 1 | Non-significant |
| Psoriasis vulgaris | ebi-a-GCST90001692 | -0.244 | 0.000776 | 0.00429 | 0.857 | -0.00318 | 1 | Non-significant |
| Type 1 diabetes | ebi-a-GCST90001888 | 0.23 | 0.000929 | 0.00514 | 0.857 | 0.00404 | 1 | Non-significant |
| Type 1 diabetes | ebi-a-GCST90001813 | 0.23 | 0.000649 | 0.0036 | 0.857 | 0.00283 | 1 | Non-significant |
| Psoriasis vulgaris | ebi-a-GCST90001691 | -0.244 | -0.00205 | 0.0114 | 0.857 | 0.00841 | 1 | Non-significant |
| Type 1 diabetes | ebi-a-GCST90001735 | 0.23 | 0.00136 | 0.00759 | 0.858 | 0.00591 | 1 | Non-significant |
| Type 1 diabetes | ebi-a-GCST90001861 | 0.23 | -0.00147 | 0.00822 | 0.858 | -0.00639 | 1 | Non-significant |
| Psoriasis vulgaris | ebi-a-GCST90001990 | -0.244 | 0.00232 | 0.013 | 0.859 | -0.0095 | 1 | Non-significant |
| Psoriasis vulgaris | ebi-a-GCST90001516 | -0.244 | -0.00076 | 0.00432 | 0.86 | 0.00312 | 1 | Non-significant |
| Psoriasis vulgaris | ebi-a-GCST90001507 | -0.244 | 0.00169 | 0.0096 | 0.861 | -0.00692 | 1 | Non-significant |
| Type 1 diabetes | ebi-a-GCST90001543 | 0.23 | -0.00427 | 0.0244 | 0.861 | -0.0186 | 1 | Non-significant |
| Psoriasis vulgaris | ebi-a-GCST90001954 | -0.244 | -0.00046 | 0.00268 | 0.863 | 0.0019 | 1 | Non-significant |
| Type 1 diabetes | ebi-a-GCST90001692 | 0.23 | 0.000576 | 0.00334 | 0.863 | 0.0025 | 1 | Non-significant |
| Psoriasis vulgaris | ebi-a-GCST90001735 | -0.244 | 0.000774 | 0.00449 | 0.863 | -0.00317 | 1 | Non-significant |
| Type 1 diabetes | ebi-a-GCST90001679 | 0.23 | -0.00085 | 0.00496 | 0.864 | -0.00369 | 1 | Non-significant |
| Type 1 diabetes | ebi-a-GCST90001973 | 0.23 | -0.00387 | 0.0227 | 0.865 | -0.0168 | 1 | Non-significant |
| Psoriasis vulgaris | ebi-a-GCST90001881 | -0.244 | 0.00508 | 0.0299 | 0.865 | -0.0209 | 1 | Non-significant |
| Type 1 diabetes | ebi-a-GCST90001966 | 0.23 | -0.00286 | 0.0168 | 0.865 | -0.0124 | 1 | Non-significant |
| Psoriasis vulgaris | ebi-a-GCST90001887 | -0.244 | 0.000754 | 0.00444 | 0.865 | -0.00309 | 1 | Non-significant |
| Psoriasis vulgaris | ebi-a-GCST90001879 | -0.244 | -0.00408 | 0.0241 | 0.866 | 0.0167 | 1 | Non-significant |
| Type 1 diabetes | ebi-a-GCST90001531 | 0.23 | -0.00063 | 0.00375 | 0.866 | -0.00276 | 1 | Non-significant |
| Psoriasis vulgaris | ebi-a-GCST90001554 | -0.244 | -0.00149 | 0.00881 | 0.866 | 0.0061 | 1 | Non-significant |
| Psoriasis vulgaris | ebi-a-GCST90001848 | -0.244 | 0.0027 | 0.0161 | 0.867 | -0.0111 | 1 | Non-significant |
| Psoriasis vulgaris | ebi-a-GCST90001534 | -0.244 | 0.00196 | 0.0117 | 0.867 | -0.00803 | 1 | Non-significant |
| Type 1 diabetes | ebi-a-GCST90001585 | 0.23 | 0.00301 | 0.0181 | 0.868 | 0.0131 | 1 | Non-significant |
| Psoriasis vulgaris | ebi-a-GCST90002039 | -0.244 | 0.00122 | 0.00736 | 0.868 | -0.00501 | 1 | Non-significant |
| Type 1 diabetes | ebi-a-GCST90001633 | 0.23 | 0.000497 | 0.00299 | 0.868 | 0.00216 | 1 | Non-significant |
| Psoriasis vulgaris | ebi-a-GCST90002032 | -0.244 | -0.00029 | 0.00176 | 0.868 | 0.0012 | 1 | Non-significant |
| Type 1 diabetes | ebi-a-GCST90001879 | 0.23 | 0.00189 | 0.0114 | 0.869 | 0.00821 | 1 | Non-significant |
| Type 1 diabetes | ebi-a-GCST90001723 | 0.23 | -0.00056 | 0.00343 | 0.869 | -0.00245 | 1 | Non-significant |
| Type 1 diabetes | ebi-a-GCST90002077 | 0.23 | 0.00293 | 0.0178 | 0.87 | 0.0127 | 1 | Non-significant |
| Psoriasis vulgaris | ebi-a-GCST90001825 | -0.244 | 0.000384 | 0.00234 | 0.87 | -0.00157 | 1 | Non-significant |
| Psoriasis vulgaris | ebi-a-GCST90001517 | -0.244 | 0.00146 | 0.00895 | 0.87 | -0.00599 | 1 | Non-significant |
| Psoriasis vulgaris | ebi-a-GCST90001903 | -0.244 | -0.00385 | 0.0237 | 0.871 | 0.0158 | 1 | Non-significant |
| Psoriasis vulgaris | ebi-a-GCST90002006 | -0.244 | -0.00258 | 0.016 | 0.871 | 0.0106 | 1 | Non-significant |
| Type 1 diabetes | ebi-a-GCST90001470 | 0.23 | 0.00243 | 0.0151 | 0.872 | 0.0106 | 1 | Non-significant |
| Psoriasis vulgaris | ebi-a-GCST90001963 | -0.244 | -0.00206 | 0.0129 | 0.873 | 0.00845 | 1 | Non-significant |
| Type 1 diabetes | ebi-a-GCST90001703 | 0.23 | 0.000615 | 0.00387 | 0.874 | 0.00268 | 1 | Non-significant |
| Psoriasis vulgaris | ebi-a-GCST90001909 | -0.244 | 0.00332 | 0.0209 | 0.874 | -0.0136 | 1 | Non-significant |
| Type 1 diabetes | ebi-a-GCST90001952 | 0.23 | -0.00063 | 0.00399 | 0.874 | -0.00276 | 1 | Non-significant |
| Type 1 diabetes | ebi-a-GCST90001963 | 0.23 | 0.00166 | 0.0105 | 0.874 | 0.00723 | 1 | Non-significant |
| Type 1 diabetes | ebi-a-GCST90002069 | 0.23 | -0.00243 | 0.0154 | 0.874 | -0.0106 | 1 | Non-significant |
| Psoriasis vulgaris | ebi-a-GCST90001621 | -0.244 | -0.00135 | 0.00858 | 0.875 | 0.00555 | 1 | Non-significant |
| Psoriasis vulgaris | ebi-a-GCST90001952 | -0.244 | -0.00056 | 0.00355 | 0.875 | 0.0023 | 1 | Non-significant |
| Type 1 diabetes | ebi-a-GCST90001848 | 0.23 | 0.000933 | 0.00597 | 0.876 | 0.00406 | 1 | Non-significant |
| Psoriasis vulgaris | ebi-a-GCST90001816 | -0.244 | 0.000498 | 0.00319 | 0.876 | -0.00204 | 1 | Non-significant |
| Psoriasis vulgaris | ebi-a-GCST90002069 | -0.244 | -0.00171 | 0.011 | 0.876 | 0.00702 | 1 | Non-significant |
| Type 1 diabetes | ebi-a-GCST90001983 | 0.23 | -0.0037 | 0.0238 | 0.876 | -0.0161 | 1 | Non-significant |
| Psoriasis vulgaris | ebi-a-GCST90001723 | -0.244 | 0.000468 | 0.00302 | 0.877 | -0.00192 | 1 | Non-significant |
| Psoriasis vulgaris | ebi-a-GCST90002061 | -0.244 | -0.00135 | 0.00882 | 0.878 | 0.00555 | 1 | Non-significant |
| Psoriasis vulgaris | ebi-a-GCST90002030 | -0.244 | -0.00049 | 0.0032 | 0.879 | 0.002 | 1 | Non-significant |
| Psoriasis vulgaris | ebi-a-GCST90001813 | -0.244 | 0.00038 | 0.00249 | 0.879 | -0.00156 | 1 | Non-significant |
| Type 1 diabetes | ebi-a-GCST90001782 | 0.23 | -0.00109 | 0.00719 | 0.879 | -0.00476 | 1 | Non-significant |
| Type 1 diabetes | ebi-a-GCST90001790 | 0.23 | 0.00228 | 0.0151 | 0.879 | 0.00994 | 1 | Non-significant |
| Psoriasis vulgaris | ebi-a-GCST90001745 | -0.244 | -0.00348 | 0.0231 | 0.88 | 0.0143 | 1 | Non-significant |
| Type 1 diabetes | ebi-a-GCST90001777 | 0.23 | 0.00186 | 0.0124 | 0.881 | 0.00809 | 1 | Non-significant |
| Type 1 diabetes | ebi-a-GCST90001807 | 0.23 | 0.00514 | 0.0343 | 0.881 | 0.0224 | 1 | Non-significant |
| Psoriasis vulgaris | ebi-a-GCST90001888 | -0.244 | -0.00101 | 0.00682 | 0.882 | 0.00416 | 1 | Non-significant |
| Psoriasis vulgaris | ebi-a-GCST90001896 | -0.244 | 0.000563 | 0.0038 | 0.882 | -0.00231 | 1 | Non-significant |
| Psoriasis vulgaris | ebi-a-GCST90001790 | -0.244 | 0.000641 | 0.00433 | 0.882 | -0.00263 | 1 | Non-significant |
| Type 1 diabetes | ebi-a-GCST90001991 | 0.23 | 0.00502 | 0.034 | 0.883 | 0.0219 | 1 | Non-significant |
| Psoriasis vulgaris | ebi-a-GCST90001422 | -0.244 | -0.00198 | 0.0135 | 0.884 | 0.00811 | 1 | Non-significant |
| Psoriasis vulgaris | ebi-a-GCST90001815 | -0.244 | -0.00039 | 0.00268 | 0.884 | 0.0016 | 1 | Non-significant |
| Psoriasis vulgaris | ebi-a-GCST90001996 | -0.244 | 0.0015 | 0.0103 | 0.884 | -0.00617 | 1 | Non-significant |
| Type 1 diabetes | ebi-a-GCST90001665 | 0.23 | -0.00087 | 0.00597 | 0.884 | -0.00378 | 1 | Non-significant |
| Type 1 diabetes | ebi-a-GCST90002036 | 0.23 | 0.00165 | 0.0113 | 0.884 | 0.00717 | 1 | Non-significant |
| Psoriasis vulgaris | ebi-a-GCST90002071 | -0.244 | -0.00086 | 0.00592 | 0.885 | 0.00351 | 1 | Non-significant |
| Type 1 diabetes | ebi-a-GCST90001956 | 0.23 | -0.00045 | 0.00315 | 0.885 | -0.00197 | 1 | Non-significant |
| Type 1 diabetes | ebi-a-GCST90001457 | 0.23 | -0.0023 | 0.0161 | 0.886 | -0.01 | 1 | Non-significant |
| Type 1 diabetes | ebi-a-GCST90001667 | 0.23 | 0.00115 | 0.00804 | 0.887 | 0.00499 | 1 | Non-significant |
| Psoriasis vulgaris | ebi-a-GCST90001667 | -0.244 | 0.00107 | 0.0075 | 0.887 | -0.00439 | 1 | Non-significant |
| Type 1 diabetes | ebi-a-GCST90001562 | 0.23 | -0.00044 | 0.0031 | 0.887 | -0.00191 | 1 | Non-significant |
| Psoriasis vulgaris | ebi-a-GCST90001480 | -0.244 | 0.000991 | 0.00703 | 0.888 | -0.00406 | 1 | Non-significant |
| Type 1 diabetes | ebi-a-GCST90001957 | 0.23 | -0.00046 | 0.00324 | 0.888 | -0.00198 | 1 | Non-significant |
| Psoriasis vulgaris | ebi-a-GCST90001956 | -0.244 | -0.00032 | 0.00224 | 0.888 | 0.00129 | 1 | Non-significant |
| Type 1 diabetes | ebi-a-GCST90001947 | 0.23 | -0.00033 | 0.00235 | 0.888 | -0.00144 | 1 | Non-significant |
| Psoriasis vulgaris | ebi-a-GCST90001528 | -0.244 | 0.00192 | 0.0137 | 0.888 | -0.00788 | 1 | Non-significant |
| Type 1 diabetes | ebi-a-GCST90001422 | 0.23 | -0.00104 | 0.00745 | 0.889 | -0.00454 | 1 | Non-significant |
| Psoriasis vulgaris | ebi-a-GCST90001665 | -0.244 | -0.00062 | 0.0044 | 0.889 | 0.00252 | 1 | Non-significant |
| Psoriasis vulgaris | ebi-a-GCST90002036 | -0.244 | 0.000953 | 0.00685 | 0.889 | -0.00391 | 1 | Non-significant |
| Type 1 diabetes | ebi-a-GCST90001803 | 0.23 | 0.00089 | 0.00641 | 0.89 | 0.00387 | 1 | Non-significant |
| Type 1 diabetes | ebi-a-GCST90001792 | 0.23 | 0.00167 | 0.0122 | 0.89 | 0.00729 | 1 | Non-significant |
| Type 1 diabetes | ebi-a-GCST90001854 | 0.23 | -0.00101 | 0.00738 | 0.891 | -0.0044 | 1 | Non-significant |
| Psoriasis vulgaris | ebi-a-GCST90001520 | -0.244 | 0.00219 | 0.016 | 0.891 | -0.00898 | 1 | Non-significant |
| Psoriasis vulgaris | ebi-a-GCST90001957 | -0.244 | -0.00028 | 0.00202 | 0.891 | 0.00113 | 1 | Non-significant |
| Psoriasis vulgaris | ebi-a-GCST90001949 | -0.244 | -0.00034 | 0.00245 | 0.891 | 0.00137 | 1 | Non-significant |
| Type 1 diabetes | ebi-a-GCST90001881 | 0.23 | -0.00069 | 0.00503 | 0.892 | -0.00298 | 1 | Non-significant |
| Psoriasis vulgaris | ebi-a-GCST90001625 | -0.244 | -0.00243 | 0.0178 | 0.892 | 0.00995 | 1 | Non-significant |
| Type 1 diabetes | ebi-a-GCST90001435 | 0.23 | -0.00471 | 0.0347 | 0.892 | -0.0205 | 1 | Non-significant |
| Type 1 diabetes | ebi-a-GCST90001625 | 0.23 | 0.00193 | 0.0143 | 0.892 | 0.0084 | 1 | Non-significant |
| Type 1 diabetes | ebi-a-GCST90001637 | 0.23 | 0.000588 | 0.00437 | 0.893 | 0.00256 | 1 | Non-significant |
| Psoriasis vulgaris | ebi-a-GCST90002100 | -0.244 | -0.00175 | 0.013 | 0.893 | 0.00716 | 1 | Non-significant |
| Psoriasis vulgaris | ebi-a-GCST90001510 | -0.244 | -0.00042 | 0.00317 | 0.894 | 0.00174 | 1 | Non-significant |
| Psoriasis vulgaris | ebi-a-GCST90001792 | -0.244 | -0.00047 | 0.00355 | 0.894 | 0.00195 | 1 | Non-significant |
| Type 1 diabetes | ebi-a-GCST90001995 | 0.23 | -0.00098 | 0.00736 | 0.894 | -0.00428 | 1 | Non-significant |
| Psoriasis vulgaris | ebi-a-GCST90001966 | -0.244 | -0.0006 | 0.00453 | 0.895 | 0.00246 | 1 | Non-significant |
| Psoriasis vulgaris | ebi-a-GCST90001777 | -0.244 | 0.000357 | 0.00271 | 0.895 | -0.00146 | 1 | Non-significant |
| Psoriasis vulgaris | ebi-a-GCST90001831 | -0.244 | -0.00117 | 0.00912 | 0.898 | 0.00481 | 1 | Non-significant |
| Psoriasis vulgaris | ebi-a-GCST90001765 | -0.244 | -0.00026 | 0.00203 | 0.898 | 0.00107 | 1 | Non-significant |
| Psoriasis vulgaris | ebi-a-GCST90001679 | -0.244 | 0.000568 | 0.00445 | 0.898 | -0.00233 | 1 | Non-significant |
| Psoriasis vulgaris | ebi-a-GCST90001947 | -0.244 | -0.00016 | 0.00129 | 0.899 | 0.000675 | 1 | Non-significant |
| Psoriasis vulgaris | ebi-a-GCST90001435 | -0.244 | 0.00128 | 0.0101 | 0.899 | -0.00526 | 1 | Non-significant |
| Type 1 diabetes | ebi-a-GCST90001896 | 0.23 | 0.000868 | 0.00684 | 0.899 | 0.00378 | 1 | Non-significant |
| Type 1 diabetes | ebi-a-GCST90001668 | 0.23 | -0.00096 | 0.00772 | 0.901 | -0.00418 | 1 | Non-significant |
| Psoriasis vulgaris | ebi-a-GCST90001518 | -0.244 | 0.000407 | 0.00329 | 0.901 | -0.00167 | 1 | Non-significant |
| Type 1 diabetes | ebi-a-GCST90001773 | 0.23 | -0.0032 | 0.0258 | 0.901 | -0.0139 | 1 | Non-significant |
| Psoriasis vulgaris | ebi-a-GCST90001668 | -0.244 | 0.000583 | 0.00474 | 0.902 | -0.00239 | 1 | Non-significant |
| Psoriasis vulgaris | ebi-a-GCST90001819 | -0.244 | 0.000444 | 0.00361 | 0.902 | -0.00182 | 1 | Non-significant |
| Psoriasis vulgaris | ebi-a-GCST90001773 | -0.244 | 0.00134 | 0.0109 | 0.902 | -0.0055 | 1 | Non-significant |
| Type 1 diabetes | ebi-a-GCST90001778 | 0.23 | 0.00154 | 0.0126 | 0.903 | 0.00672 | 1 | Non-significant |
| Type 1 diabetes | ebi-a-GCST90001717 | 0.23 | 0.000326 | 0.00268 | 0.903 | 0.00142 | 1 | Non-significant |
| Psoriasis vulgaris | ebi-a-GCST90001778 | -0.244 | -0.00061 | 0.00501 | 0.904 | 0.00249 | 1 | Non-significant |
| Psoriasis vulgaris | ebi-a-GCST90001645 | -0.244 | 0.00164 | 0.0136 | 0.904 | -0.00674 | 1 | Non-significant |
| Psoriasis vulgaris | ebi-a-GCST90001847 | -0.244 | 0.000904 | 0.00751 | 0.904 | -0.00371 | 1 | Non-significant |
| Psoriasis vulgaris | ebi-a-GCST90002080 | -0.244 | -0.00523 | 0.0435 | 0.904 | 0.0215 | 1 | Non-significant |
| Psoriasis vulgaris | ebi-a-GCST90001407 | -0.244 | -0.0157 | 0.13 | 0.904 | 0.0643 | 1 | Non-significant |
| Type 1 diabetes | ebi-a-GCST90002121 | 0.23 | 0.00148 | 0.0124 | 0.905 | 0.00642 | 1 | Non-significant |
| Type 1 diabetes | ebi-a-GCST90001771 | 0.23 | -0.00151 | 0.0127 | 0.905 | -0.00658 | 1 | Non-significant |
| Psoriasis vulgaris | ebi-a-GCST90001398 | -0.244 | 0.00598 | 0.0505 | 0.906 | -0.0245 | 1 | Non-significant |
| Type 1 diabetes | ebi-a-GCST90001882 | 0.23 | -0.00247 | 0.021 | 0.906 | -0.0107 | 1 | Non-significant |
| Psoriasis vulgaris | ebi-a-GCST90001614 | -0.244 | 0.00156 | 0.0133 | 0.906 | -0.00641 | 1 | Non-significant |
| Type 1 diabetes | ebi-a-GCST90001873 | 0.23 | -0.00223 | 0.0189 | 0.906 | -0.0097 | 1 | Non-significant |
| Type 1 diabetes | ebi-a-GCST90001886 | 0.23 | -0.00059 | 0.005 | 0.906 | -0.00256 | 1 | Non-significant |
| Psoriasis vulgaris | ebi-a-GCST90002121 | -0.244 | -0.00076 | 0.00647 | 0.907 | 0.0031 | 1 | Non-significant |
| Psoriasis vulgaris | ebi-a-GCST90001871 | -0.244 | 0.00175 | 0.015 | 0.907 | -0.00718 | 1 | Non-significant |
| Psoriasis vulgaris | ebi-a-GCST90001876 | -0.244 | 0.00168 | 0.0144 | 0.907 | -0.0069 | 1 | Non-significant |
| Psoriasis vulgaris | ebi-a-GCST90001880 | -0.244 | 0.00142 | 0.0122 | 0.907 | -0.00582 | 1 | Non-significant |
| Type 1 diabetes | ebi-a-GCST90001876 | 0.23 | -0.00129 | 0.0111 | 0.908 | -0.00561 | 1 | Non-significant |
| Psoriasis vulgaris | ebi-a-GCST90001882 | -0.244 | 0.00131 | 0.0112 | 0.908 | -0.00536 | 1 | Non-significant |
| Type 1 diabetes | ebi-a-GCST90001786 | 0.23 | -0.0024 | 0.0208 | 0.908 | -0.0105 | 1 | Non-significant |
| Type 1 diabetes | ebi-a-GCST90001880 | 0.23 | -0.00096 | 0.00833 | 0.908 | -0.00418 | 1 | Non-significant |
| Psoriasis vulgaris | ebi-a-GCST90001873 | -0.244 | 0.00151 | 0.0131 | 0.909 | -0.00618 | 1 | Non-significant |
| Psoriasis vulgaris | ebi-a-GCST90001771 | -0.244 | -0.00055 | 0.0048 | 0.909 | 0.00226 | 1 | Non-significant |
| Type 1 diabetes | ebi-a-GCST90001485 | 0.23 | -0.00024 | 0.00206 | 0.909 | -0.00102 | 1 | Non-significant |
| Type 1 diabetes | ebi-a-GCST90001737 | 0.23 | -0.00667 | 0.0589 | 0.91 | -0.029 | 1 | Non-significant |
| Type 1 diabetes | ebi-a-GCST90001522 | 0.23 | -0.00194 | 0.0173 | 0.911 | -0.00846 | 1 | Non-significant |
| Psoriasis vulgaris | ebi-a-GCST90001958 | -0.244 | 0.00141 | 0.0127 | 0.912 | -0.00579 | 1 | Non-significant |
| Psoriasis vulgaris | ebi-a-GCST90001503 | -0.244 | 0.000444 | 0.004 | 0.912 | -0.00182 | 1 | Non-significant |
| Type 1 diabetes | ebi-a-GCST90001690 | 0.23 | 0.00324 | 0.0294 | 0.912 | 0.0141 | 1 | Non-significant |
| Psoriasis vulgaris | ebi-a-GCST90001737 | -0.244 | 0.00303 | 0.0277 | 0.913 | -0.0124 | 1 | Non-significant |
| Type 1 diabetes | ebi-a-GCST90001464 | 0.23 | 0.000968 | 0.00894 | 0.914 | 0.00421 | 1 | Non-significant |
| Psoriasis vulgaris | ebi-a-GCST90002117 | -0.244 | -0.0022 | 0.0204 | 0.914 | 0.00904 | 1 | Non-significant |
| Psoriasis vulgaris | ebi-a-GCST90002074 | -0.244 | -0.00052 | 0.00482 | 0.914 | 0.00213 | 1 | Non-significant |
| Type 1 diabetes | ebi-a-GCST90001958 | 0.23 | 0.00067 | 0.00621 | 0.914 | 0.00291 | 1 | Non-significant |
| Type 1 diabetes | ebi-a-GCST90001659 | 0.23 | -0.00038 | 0.00354 | 0.915 | -0.00165 | 1 | Non-significant |
| Type 1 diabetes | ebi-a-GCST90001924 | 0.23 | 0.00148 | 0.0138 | 0.915 | 0.00643 | 1 | Non-significant |
| Type 1 diabetes | ebi-a-GCST90001724 | 0.23 | -0.00068 | 0.00637 | 0.915 | -0.00295 | 1 | Non-significant |
| Psoriasis vulgaris | ebi-a-GCST90001983 | -0.244 | 0.000876 | 0.00829 | 0.916 | -0.00359 | 1 | Non-significant |
| Psoriasis vulgaris | ebi-a-GCST90002096 | -0.244 | 0.00107 | 0.0101 | 0.916 | -0.00437 | 1 | Non-significant |
| Psoriasis vulgaris | ebi-a-GCST90001843 | -0.244 | 0.00189 | 0.0182 | 0.917 | -0.00775 | 1 | Non-significant |
| Psoriasis vulgaris | ebi-a-GCST90001724 | -0.244 | 0.000347 | 0.00336 | 0.918 | -0.00142 | 1 | Non-significant |
| Psoriasis vulgaris | ebi-a-GCST90001598 | -0.244 | -0.0029 | 0.0281 | 0.918 | 0.0119 | 1 | Non-significant |
| Psoriasis vulgaris | ebi-a-GCST90001690 | -0.244 | 0.000612 | 0.00595 | 0.918 | -0.00251 | 1 | Non-significant |
| Type 1 diabetes | ebi-a-GCST90001521 | 0.23 | 0.000978 | 0.0096 | 0.919 | 0.00426 | 1 | Non-significant |
| Type 1 diabetes | ebi-a-GCST90001536 | 0.23 | 0.000908 | 0.00904 | 0.92 | 0.00395 | 1 | Non-significant |
| Psoriasis vulgaris | ebi-a-GCST90002065 | -0.244 | 0.00573 | 0.0571 | 0.92 | -0.0235 | 1 | Non-significant |
| Type 1 diabetes | ebi-a-GCST90002101 | 0.23 | -0.00441 | 0.0439 | 0.92 | -0.0192 | 1 | Non-significant |
| Type 1 diabetes | ebi-a-GCST90001741 | 0.23 | 0.00137 | 0.0137 | 0.92 | 0.00596 | 1 | Non-significant |
| Type 1 diabetes | ebi-a-GCST90002016 | 0.23 | 0.000442 | 0.00442 | 0.92 | 0.00192 | 1 | Non-significant |
| Psoriasis vulgaris | ebi-a-GCST90002101 | -0.244 | 0.00269 | 0.027 | 0.921 | -0.011 | 1 | Non-significant |
| Type 1 diabetes | ebi-a-GCST90002017 | 0.23 | -0.00031 | 0.00314 | 0.921 | -0.00136 | 1 | Non-significant |
| Psoriasis vulgaris | ebi-a-GCST90001803 | -0.244 | -0.0008 | 0.00804 | 0.921 | 0.00326 | 1 | Non-significant |
| Psoriasis vulgaris | ebi-a-GCST90002031 | -0.244 | -0.00037 | 0.00373 | 0.922 | 0.0015 | 1 | Non-significant |
| Psoriasis vulgaris | ebi-a-GCST90001886 | -0.244 | -0.00021 | 0.00214 | 0.924 | 0.000841 | 1 | Non-significant |
| Psoriasis vulgaris | ebi-a-GCST90001743 | -0.244 | -0.00225 | 0.0235 | 0.924 | 0.00923 | 1 | Non-significant |
| Type 1 diabetes | ebi-a-GCST90001511 | 0.23 | -0.00093 | 0.00976 | 0.924 | -0.00404 | 1 | Non-significant |
| Psoriasis vulgaris | ebi-a-GCST90001611 | -0.244 | -0.00107 | 0.0113 | 0.925 | 0.00439 | 1 | Non-significant |
| Type 1 diabetes | ebi-a-GCST90001553 | 0.23 | 0.000148 | 0.00157 | 0.925 | 0.000646 | 1 | Non-significant |
| Psoriasis vulgaris | ebi-a-GCST90001772 | -0.244 | -0.00018 | 0.00189 | 0.926 | 0.000723 | 1 | Non-significant |
| Psoriasis vulgaris | ebi-a-GCST90001526 | -0.244 | 0.00124 | 0.0134 | 0.926 | -0.00509 | 1 | Non-significant |
| Type 1 diabetes | ebi-a-GCST90001918 | 0.23 | 0.000518 | 0.0056 | 0.926 | 0.00226 | 1 | Non-significant |
| Type 1 diabetes | ebi-a-GCST90001495 | 0.23 | 0.000113 | 0.00123 | 0.926 | 0.000493 | 1 | Non-significant |
| Type 1 diabetes | ebi-a-GCST90002109 | 0.23 | 0.00286 | 0.031 | 0.927 | 0.0124 | 1 | Non-significant |
| Type 1 diabetes | ebi-a-GCST90001439 | 0.23 | -0.00867 | 0.0948 | 0.927 | -0.0377 | 1 | Non-significant |
| Psoriasis vulgaris | ebi-a-GCST90001485 | -0.244 | 7.74E-05 | 0.000847 | 0.927 | -0.00032 | 1 | Non-significant |
| Type 1 diabetes | ebi-a-GCST90001506 | 0.23 | -0.00067 | 0.00734 | 0.928 | -0.0029 | 1 | Non-significant |
| Psoriasis vulgaris | ebi-a-GCST90002038 | -0.244 | -0.00062 | 0.00702 | 0.93 | 0.00255 | 1 | Non-significant |
| Type 1 diabetes | ebi-a-GCST90001467 | 0.23 | 0.00337 | 0.0385 | 0.93 | 0.0147 | 1 | Non-significant |
| Type 1 diabetes | ebi-a-GCST90001875 | 0.23 | -0.00026 | 0.00304 | 0.931 | -0.00114 | 1 | Non-significant |
| Psoriasis vulgaris | ebi-a-GCST90001506 | -0.244 | 0.000671 | 0.00782 | 0.932 | -0.00275 | 1 | Non-significant |
| Psoriasis vulgaris | ebi-a-GCST90001570 | -0.244 | -0.00047 | 0.00554 | 0.932 | 0.00194 | 1 | Non-significant |
| Psoriasis vulgaris | ebi-a-GCST90002059 | -0.244 | -0.00042 | 0.00497 | 0.933 | 0.00172 | 1 | Non-significant |
| Type 1 diabetes | ebi-a-GCST90001436 | 0.23 | -0.00482 | 0.0577 | 0.933 | -0.021 | 1 | Non-significant |
| Psoriasis vulgaris | ebi-a-GCST90001436 | -0.244 | 0.00409 | 0.0491 | 0.934 | -0.0168 | 1 | Non-significant |
| Psoriasis vulgaris | ebi-a-GCST90001553 | -0.244 | 2.48E-05 | 0.000299 | 0.934 | -0.0001 | 1 | Non-significant |
| Psoriasis vulgaris | ebi-a-GCST90001781 | -0.244 | -0.00039 | 0.00468 | 0.935 | 0.00158 | 1 | Non-significant |
| Type 1 diabetes | ebi-a-GCST90001810 | 0.23 | -0.00073 | 0.00885 | 0.935 | -0.00316 | 1 | Non-significant |
| Psoriasis vulgaris | ebi-a-GCST90001752 | -0.244 | 0.00169 | 0.021 | 0.936 | -0.00694 | 1 | Non-significant |
| Type 1 diabetes | ebi-a-GCST90001733 | 0.23 | -0.00342 | 0.0425 | 0.936 | -0.0149 | 1 | Non-significant |
| Psoriasis vulgaris | ebi-a-GCST90001693 | -0.244 | -0.00033 | 0.0041 | 0.936 | 0.00135 | 1 | Non-significant |
| Psoriasis vulgaris | ebi-a-GCST90001810 | -0.244 | ####### | 0.00879 | 0.936 | 0.00287 | 1 | Non-significant |
| Psoriasis vulgaris | ebi-a-GCST90001863 | -0.244 | 0.00129 | 0.0162 | 0.937 | -0.00529 | 1 | Non-significant |
| Psoriasis vulgaris | ebi-a-GCST90001910 | -0.244 | -0.00216 | 0.0276 | 0.938 | 0.00884 | 1 | Non-significant |
| Psoriasis vulgaris | ebi-a-GCST90001741 | -0.244 | -0.00034 | 0.00441 | 0.938 | 0.0014 | 1 | Non-significant |
| Type 1 diabetes | ebi-a-GCST90001910 | 0.23 | 0.000939 | 0.0121 | 0.938 | 0.00409 | 1 | Non-significant |
| Type 1 diabetes | ebi-a-GCST90002059 | 0.23 | -0.00023 | 0.00297 | 0.939 | -0.00099 | 1 | Non-significant |
| Psoriasis vulgaris | ebi-a-GCST90001644 | -0.244 | -0.00448 | 0.0585 | 0.939 | 0.0184 | 1 | Non-significant |
| Type 1 diabetes | ebi-a-GCST90002009 | 0.23 | 0.00159 | 0.0211 | 0.94 | 0.00692 | 1 | Non-significant |
| Psoriasis vulgaris | ebi-a-GCST90001799 | -0.244 | 0.00181 | 0.0243 | 0.941 | -0.00743 | 1 | Non-significant |
| Type 1 diabetes | ebi-a-GCST90001644 | 0.23 | 0.000893 | 0.012 | 0.941 | 0.00389 | 1 | Non-significant |
| Type 1 diabetes | ebi-a-GCST90001748 | 0.23 | -0.0013 | 0.0175 | 0.941 | -0.00566 | 1 | Non-significant |
| Type 1 diabetes | ebi-a-GCST90002081 | 0.23 | 0.00106 | 0.0143 | 0.941 | 0.00461 | 1 | Non-significant |
| Type 1 diabetes | ebi-a-GCST90001512 | 0.23 | 0.000276 | 0.00379 | 0.942 | 0.0012 | 1 | Non-significant |
| Type 1 diabetes | ebi-a-GCST90001828 | 0.23 | 0.00028 | 0.00387 | 0.942 | 0.00122 | 1 | Non-significant |
| Psoriasis vulgaris | ebi-a-GCST90001828 | -0.244 | 0.000248 | 0.00345 | 0.943 | -0.00102 | 1 | Non-significant |
| Psoriasis vulgaris | ebi-a-GCST90001779 | -0.244 | 0.000743 | 0.0104 | 0.943 | -0.00305 | 1 | Non-significant |
| Psoriasis vulgaris | ebi-a-GCST90001439 | -0.244 | 0.000577 | 0.00805 | 0.943 | -0.00237 | 1 | Non-significant |
| Type 1 diabetes | ebi-a-GCST90002097 | 0.23 | -0.00128 | 0.0182 | 0.944 | -0.00555 | 1 | Non-significant |
| Type 1 diabetes | ebi-a-GCST90001779 | 0.23 | -0.00045 | 0.00636 | 0.944 | -0.00193 | 1 | Non-significant |
| Type 1 diabetes | ebi-a-GCST90002088 | 0.23 | -0.00259 | 0.0371 | 0.944 | -0.0113 | 1 | Non-significant |
| Psoriasis vulgaris | ebi-a-GCST90002088 | -0.244 | 0.00139 | 0.02 | 0.944 | -0.00571 | 1 | Non-significant |
| Type 1 diabetes | ebi-a-GCST90001754 | 0.23 | 0.00243 | 0.0349 | 0.945 | 0.0106 | 1 | Non-significant |
| Psoriasis vulgaris | ebi-a-GCST90001754 | -0.244 | -0.0007 | 0.0102 | 0.946 | 0.00286 | 1 | Non-significant |
| Psoriasis vulgaris | ebi-a-GCST90001759 | -0.244 | 0.00175 | 0.0258 | 0.946 | -0.00718 | 1 | Non-significant |
| Type 1 diabetes | ebi-a-GCST90001902 | 0.23 | -0.00058 | 0.00868 | 0.947 | -0.00251 | 1 | Non-significant |
| Type 1 diabetes | ebi-a-GCST90001889 | 0.23 | -0.00044 | 0.00668 | 0.947 | -0.00193 | 1 | Non-significant |
| Psoriasis vulgaris | ebi-a-GCST90001902 | -0.244 | -0.00038 | 0.0058 | 0.947 | 0.00157 | 1 | Non-significant |
| Psoriasis vulgaris | ebi-a-GCST90001610 | -0.244 | -0.00074 | 0.0113 | 0.947 | 0.00305 | 1 | Non-significant |
| Psoriasis vulgaris | ebi-a-GCST90001948 | -0.244 | -0.00021 | 0.00316 | 0.948 | 0.000845 | 1 | Non-significant |
| Type 1 diabetes | ebi-a-GCST90002015 | 0.23 | 0.000283 | 0.00438 | 0.949 | 0.00123 | 1 | Non-significant |
| Type 1 diabetes | ebi-a-GCST90001844 | 0.23 | -0.00078 | 0.0122 | 0.949 | -0.00341 | 1 | Non-significant |
| Type 1 diabetes | ebi-a-GCST90001728 | 0.23 | -0.00071 | 0.011 | 0.949 | -0.00309 | 1 | Non-significant |
| Type 1 diabetes | ebi-a-GCST90001891 | 0.23 | 0.00069 | 0.0109 | 0.95 | 0.003 | 1 | Non-significant |
| Type 1 diabetes | ebi-a-GCST90001870 | 0.23 | 0.00146 | 0.0232 | 0.95 | 0.00637 | 1 | Non-significant |
| Psoriasis vulgaris | ebi-a-GCST90001870 | -0.244 | -0.00143 | 0.0227 | 0.95 | 0.00586 | 1 | Non-significant |
| Psoriasis vulgaris | ebi-a-GCST90001891 | -0.244 | 0.000365 | 0.00581 | 0.95 | -0.0015 | 1 | Non-significant |
| Type 1 diabetes | ebi-a-GCST90002033 | 0.23 | -0.00058 | 0.00934 | 0.95 | -0.00253 | 1 | Non-significant |
| Psoriasis vulgaris | ebi-a-GCST90002018 | -0.244 | 0.0025 | 0.0406 | 0.951 | -0.0103 | 1 | Non-significant |
| Type 1 diabetes | ebi-a-GCST90002018 | 0.23 | 0.000852 | 0.0138 | 0.951 | 0.00371 | 1 | Non-significant |
| Type 1 diabetes | ebi-a-GCST90002084 | 0.23 | -0.00243 | 0.0395 | 0.951 | -0.0106 | 1 | Non-significant |
| Type 1 diabetes | ebi-a-GCST90001555 | 0.23 | 0.000359 | 0.00586 | 0.951 | 0.00156 | 1 | Non-significant |
| Psoriasis vulgaris | ebi-a-GCST90001747 | -0.244 | -0.0019 | 0.0311 | 0.951 | 0.00781 | 1 | Non-significant |
| Type 1 diabetes | ebi-a-GCST90002045 | 0.23 | -0.00092 | 0.0155 | 0.953 | -0.00399 | 1 | Non-significant |
| Psoriasis vulgaris | ebi-a-GCST90002045 | -0.244 | -0.00071 | 0.0121 | 0.953 | 0.00292 | 1 | Non-significant |
| Psoriasis vulgaris | ebi-a-GCST90001483 | -0.244 | 8.23E-05 | 0.00141 | 0.953 | -0.00034 | 1 | Non-significant |
| Type 1 diabetes | ebi-a-GCST90001579 | 0.23 | 0.000669 | 0.0115 | 0.954 | 0.00291 | 1 | Non-significant |
| Psoriasis vulgaris | ebi-a-GCST90001487 | -0.244 | -0.00091 | 0.0157 | 0.954 | 0.00375 | 1 | Non-significant |
| Psoriasis vulgaris | ebi-a-GCST90001588 | -0.244 | -0.00307 | 0.0532 | 0.954 | 0.0126 | 1 | Non-significant |
| Type 1 diabetes | ebi-a-GCST90001588 | 0.23 | 0.00145 | 0.0251 | 0.954 | 0.00629 | 1 | Non-significant |
| Type 1 diabetes | ebi-a-GCST90001487 | 0.23 | 0.000158 | 0.0028 | 0.955 | 0.000689 | 1 | Non-significant |
| Type 1 diabetes | ebi-a-GCST90001990 | 0.23 | -0.00023 | 0.00404 | 0.955 | -0.001 | 1 | Non-significant |
| Psoriasis vulgaris | ebi-a-GCST90001762 | -0.244 | -0.00138 | 0.0244 | 0.955 | 0.00564 | 1 | Non-significant |
| Type 1 diabetes | ebi-a-GCST90001530 | 0.23 | 0.0013 | 0.0232 | 0.955 | 0.00564 | 1 | Non-significant |
| Type 1 diabetes | ebi-a-GCST90001590 | 0.23 | -0.00182 | 0.0333 | 0.956 | -0.00792 | 1 | Non-significant |
| Type 1 diabetes | ebi-a-GCST90001537 | 0.23 | -0.00145 | 0.0266 | 0.956 | -0.00633 | 1 | Non-significant |
| Psoriasis vulgaris | ebi-a-GCST90001590 | -0.244 | -0.00083 | 0.0152 | 0.957 | 0.00339 | 1 | Non-significant |
| Psoriasis vulgaris | ebi-a-GCST90001537 | -0.244 | -0.00084 | 0.0154 | 0.957 | 0.00344 | 1 | Non-significant |
| Psoriasis vulgaris | ebi-a-GCST90001889 | -0.244 | ####### | 0.00155 | 0.957 | 0.000342 | 1 | Non-significant |
| Type 1 diabetes | ebi-a-GCST90001867 | 0.23 | 0.000638 | 0.0123 | 0.959 | 0.00278 | 1 | Non-significant |
| Type 1 diabetes | ebi-a-GCST90001697 | 0.23 | 2.19E-06 | 4.24E-05 | 0.959 | 9.52E-06 | 1 | Non-significant |
| Type 1 diabetes | ebi-a-GCST90001781 | 0.23 | 0.000234 | 0.00455 | 0.959 | 0.00102 | 1 | Non-significant |
| Psoriasis vulgaris | ebi-a-GCST90001654 | -0.244 | -0.0016 | 0.0315 | 0.959 | 0.00656 | 1 | Non-significant |
| Type 1 diabetes | ebi-a-GCST90001654 | 0.23 | 0.00074 | 0.0146 | 0.96 | 0.00322 | 1 | Non-significant |
| Psoriasis vulgaris | ebi-a-GCST90001998 | -0.244 | -0.00208 | 0.041 | 0.96 | 0.00851 | 1 | Non-significant |
| Type 1 diabetes | ebi-a-GCST90001471 | 0.23 | 0.00121 | 0.024 | 0.96 | 0.00526 | 1 | Non-significant |
| Type 1 diabetes | ebi-a-GCST90001940 | 0.23 | 0.00121 | 0.0243 | 0.96 | 0.00525 | 1 | Non-significant |
| Psoriasis vulgaris | ebi-a-GCST90001755 | -0.244 | -0.00081 | 0.0164 | 0.96 | 0.00333 | 1 | Non-significant |
| Type 1 diabetes | ebi-a-GCST90001630 | 0.23 | 0.000849 | 0.0173 | 0.961 | 0.0037 | 1 | Non-significant |
| Psoriasis vulgaris | ebi-a-GCST90001875 | -0.244 | 0.000121 | 0.00246 | 0.961 | -0.0005 | 1 | Non-significant |
| Psoriasis vulgaris | ebi-a-GCST90001484 | -0.244 | 0.000124 | 0.00254 | 0.961 | -0.00051 | 1 | Non-significant |
| Psoriasis vulgaris | ebi-a-GCST90001974 | -0.244 | -0.00329 | 0.069 | 0.962 | 0.0135 | 1 | Non-significant |
| Psoriasis vulgaris | ebi-a-GCST90001933 | -0.244 | -0.0001 | 0.0022 | 0.962 | 0.000427 | 1 | Non-significant |
| Psoriasis vulgaris | ebi-a-GCST90002052 | -0.244 | -0.00025 | 0.00532 | 0.963 | 0.00102 | 1 | Non-significant |
| Type 1 diabetes | ebi-a-GCST90002118 | 0.23 | 0.000711 | 0.0153 | 0.963 | 0.00309 | 1 | Non-significant |
| Type 1 diabetes | ebi-a-GCST90001729 | 0.23 | -0.00038 | 0.00815 | 0.963 | -0.00164 | 1 | Non-significant |
| Psoriasis vulgaris | ebi-a-GCST90002118 | -0.244 | 0.000244 | 0.00529 | 0.963 | -0.001 | 1 | Non-significant |
| Type 1 diabetes | ebi-a-GCST90001680 | 0.23 | -0.00022 | 0.00493 | 0.964 | -0.00097 | 1 | Non-significant |
| Psoriasis vulgaris | ebi-a-GCST90001689 | -0.244 | -0.00077 | 0.017 | 0.964 | 0.00316 | 1 | Non-significant |
| Psoriasis vulgaris | ebi-a-GCST90001680 | -0.244 | -0.00014 | 0.00315 | 0.964 | 0.00058 | 1 | Non-significant |
| Type 1 diabetes | ebi-a-GCST90001900 | 0.23 | 0.00153 | 0.0341 | 0.964 | 0.00664 | 1 | Non-significant |
| Psoriasis vulgaris | ebi-a-GCST90001964 | -0.244 | 0.00033 | 0.00744 | 0.965 | -0.00135 | 1 | Non-significant |
| Type 1 diabetes | ebi-a-GCST90001711 | 0.23 | -0.0002 | 0.00443 | 0.965 | -0.00085 | 1 | Non-significant |
| Type 1 diabetes | ebi-a-GCST90001964 | 0.23 | -0.00012 | 0.00269 | 0.965 | -0.00051 | 1 | Non-significant |
| Psoriasis vulgaris | ebi-a-GCST90001904 | -0.244 | -0.00068 | 0.0158 | 0.966 | 0.00278 | 1 | Non-significant |
| Type 1 diabetes | ebi-a-GCST90001965 | 0.23 | -0.00128 | 0.03 | 0.966 | -0.00557 | 1 | Non-significant |
| Type 1 diabetes | ebi-a-GCST90001538 | 0.23 | -0.00053 | 0.0125 | 0.966 | -0.0023 | 1 | Non-significant |
| Psoriasis vulgaris | ebi-a-GCST90001538 | -0.244 | 0.000458 | 0.0109 | 0.966 | -0.00188 | 1 | Non-significant |
| Psoriasis vulgaris | ebi-a-GCST90001498 | -0.244 | 0.000453 | 0.0108 | 0.966 | -0.00186 | 1 | Non-significant |
| Type 1 diabetes | ebi-a-GCST90001498 | 0.23 | -0.00048 | 0.0115 | 0.967 | -0.0021 | 1 | Non-significant |
| Psoriasis vulgaris | ebi-a-GCST90001522 | -0.244 | 0.000169 | 0.00404 | 0.967 | -0.00069 | 1 | Non-significant |
| Psoriasis vulgaris | ebi-a-GCST90001768 | -0.244 | 0.00077 | 0.0184 | 0.967 | -0.00316 | 1 | Non-significant |
| Type 1 diabetes | ebi-a-GCST90001730 | 0.23 | ####### | 0.00236 | 0.967 | -0.00043 | 1 | Non-significant |
| Type 1 diabetes | ebi-a-GCST90001396 | 0.23 | -0.00302 | 0.0736 | 0.967 | -0.0131 | 1 | Non-significant |
| Psoriasis vulgaris | ebi-a-GCST90001396 | -0.244 | -0.00265 | 0.0646 | 0.967 | 0.0109 | 1 | Non-significant |
| Type 1 diabetes | ebi-a-GCST90001432 | 0.23 | 0.000997 | 0.0243 | 0.967 | 0.00434 | 1 | Non-significant |
| Psoriasis vulgaris | ebi-a-GCST90001432 | -0.244 | -0.00058 | 0.0143 | 0.967 | 0.00239 | 1 | Non-significant |
| Psoriasis vulgaris | ebi-a-GCST90002064 | -0.244 | 0.000834 | 0.0205 | 0.968 | -0.00342 | 1 | Non-significant |
| Psoriasis vulgaris | ebi-a-GCST90002078 | -0.244 | -0.0005 | 0.0123 | 0.968 | 0.00203 | 1 | Non-significant |
| Type 1 diabetes | ebi-a-GCST90002078 | 0.23 | -0.0003 | 0.00745 | 0.968 | -0.00131 | 1 | Non-significant |
| Psoriasis vulgaris | ebi-a-GCST90001730 | -0.244 | 6.72E-05 | 0.00168 | 0.968 | -0.00028 | 1 | Non-significant |
| Type 1 diabetes | ebi-a-GCST90001994 | 0.23 | 0.000203 | 0.00506 | 0.968 | 0.000882 | 1 | Non-significant |
| Type 1 diabetes | ebi-a-GCST90001901 | 0.23 | -0.00072 | 0.0182 | 0.968 | -0.00314 | 1 | Non-significant |
| Psoriasis vulgaris | ebi-a-GCST90002037 | -0.244 | -0.00026 | 0.00651 | 0.969 | 0.00105 | 1 | Non-significant |
| Psoriasis vulgaris | ebi-a-GCST90001694 | -0.244 | 0.0013 | 0.0329 | 0.969 | -0.00532 | 1 | Non-significant |
| Type 1 diabetes | ebi-a-GCST90001694 | 0.23 | -0.00024 | 0.00612 | 0.969 | -0.00105 | 1 | Non-significant |
| Psoriasis vulgaris | ebi-a-GCST90001833 | -0.244 | -0.00068 | 0.0173 | 0.969 | 0.00278 | 1 | Non-significant |
| Type 1 diabetes | ebi-a-GCST90001518 | 0.23 | ####### | 0.00171 | 0.97 | -0.00028 | 1 | Non-significant |
| Psoriasis vulgaris | ebi-a-GCST90001793 | -0.244 | 0.000322 | 0.00872 | 0.971 | -0.00132 | 1 | Non-significant |
| Type 1 diabetes | ebi-a-GCST90001793 | 0.23 | 0.000465 | 0.0126 | 0.971 | 0.00203 | 1 | Non-significant |
| Type 1 diabetes | ebi-a-GCST90001742 | 0.23 | -0.00036 | 0.00983 | 0.971 | -0.00157 | 1 | Non-significant |
| Psoriasis vulgaris | ebi-a-GCST90001729 | -0.244 | 2.23E-05 | 0.000608 | 0.971 | ####### | 1 | Non-significant |
| Psoriasis vulgaris | ebi-a-GCST90001785 | -0.244 | -0.0009 | 0.0247 | 0.971 | 0.00369 | 1 | Non-significant |
| Type 1 diabetes | ebi-a-GCST90001520 | 0.23 | 5.20E-05 | 0.00143 | 0.971 | 0.000226 | 1 | Non-significant |
| Psoriasis vulgaris | ebi-a-GCST90001742 | -0.244 | ####### | 0.00154 | 0.971 | 0.000226 | 1 | Non-significant |
| Psoriasis vulgaris | ebi-a-GCST90001661 | -0.244 | -0.00092 | 0.0266 | 0.972 | 0.00378 | 1 | Non-significant |
| Type 1 diabetes | ebi-a-GCST90001871 | 0.23 | 7.40E-05 | 0.00222 | 0.973 | 0.000322 | 1 | Non-significant |
| Type 1 diabetes | ebi-a-GCST90001458 | 0.23 | 0.000288 | 0.00866 | 0.974 | 0.00125 | 1 | Non-significant |
| Type 1 diabetes | ebi-a-GCST90001661 | 0.23 | ####### | 0.00121 | 0.974 | -0.00017 | 1 | Non-significant |
| Psoriasis vulgaris | ebi-a-GCST90001477 | -0.244 | -0.00098 | 0.0304 | 0.974 | 0.00403 | 1 | Non-significant |
| Type 1 diabetes | ebi-a-GCST90001681 | 0.23 | -0.00017 | 0.00562 | 0.976 | -0.00075 | 1 | Non-significant |
| Psoriasis vulgaris | ebi-a-GCST90001681 | -0.244 | -0.00015 | 0.00483 | 0.976 | 0.000603 | 1 | Non-significant |
| Psoriasis vulgaris | ebi-a-GCST90001569 | -0.244 | -0.00041 | 0.0147 | 0.978 | 0.00169 | 1 | Non-significant |
| Type 1 diabetes | ebi-a-GCST90001569 | 0.23 | 5.00E-05 | 0.00181 | 0.978 | 0.000218 | 1 | Non-significant |
| Type 1 diabetes | ebi-a-GCST90001893 | 0.23 | -0.00031 | 0.0113 | 0.978 | -0.00133 | 1 | Non-significant |
| Psoriasis vulgaris | ebi-a-GCST90001893 | -0.244 | -0.00023 | 0.00836 | 0.978 | 0.000934 | 1 | Non-significant |
| Psoriasis vulgaris | ebi-a-GCST90001551 | -0.244 | 0.000388 | 0.0143 | 0.978 | -0.00159 | 1 | Non-significant |
| Type 1 diabetes | ebi-a-GCST90001899 | 0.23 | 0.000143 | 0.00528 | 0.978 | 0.000623 | 1 | Non-significant |
| Type 1 diabetes | ebi-a-GCST90001738 | 0.23 | -0.00012 | 0.00438 | 0.978 | -0.00052 | 1 | Non-significant |
| Type 1 diabetes | ebi-a-GCST90001477 | 0.23 | ####### | 0.00188 | 0.979 | -0.00022 | 1 | Non-significant |
| Psoriasis vulgaris | ebi-a-GCST90001738 | -0.244 | 2.74E-05 | 0.00104 | 0.979 | -0.00011 | 1 | Non-significant |
| Psoriasis vulgaris | ebi-a-GCST90001899 | -0.244 | 3.63E-05 | 0.0014 | 0.979 | -0.00015 | 1 | Non-significant |
| Type 1 diabetes | ebi-a-GCST90001935 | 0.23 | 0.000622 | 0.024 | 0.979 | 0.00271 | 1 | Non-significant |
| Psoriasis vulgaris | ebi-a-GCST90001851 | -0.244 | -0.00033 | 0.013 | 0.98 | 0.00135 | 1 | Non-significant |
| Psoriasis vulgaris | ebi-a-GCST90002055 | -0.244 | -0.00026 | 0.0114 | 0.982 | 0.00108 | 1 | Non-significant |
| Type 1 diabetes | ebi-a-GCST90001815 | 0.23 | ####### | 0.00232 | 0.982 | -0.00023 | 1 | Non-significant |
| Psoriasis vulgaris | ebi-a-GCST90001585 | -0.244 | -0.00036 | 0.0162 | 0.982 | 0.00148 | 1 | Non-significant |
| Psoriasis vulgaris | ebi-a-GCST90001965 | -0.244 | ####### | 0.00206 | 0.982 | 0.000186 | 1 | Non-significant |
| Type 1 diabetes | ebi-a-GCST90001989 | 0.23 | 9.84E-05 | 0.0045 | 0.983 | 0.000428 | 1 | Non-significant |
| Psoriasis vulgaris | ebi-a-GCST90001622 | -0.244 | 0.000222 | 0.0104 | 0.983 | -0.00091 | 1 | Non-significant |
| Type 1 diabetes | ebi-a-GCST90001622 | 0.23 | 0.000177 | 0.00828 | 0.983 | 0.00077 | 1 | Non-significant |
| Type 1 diabetes | ebi-a-GCST90002102 | 0.23 | ####### | 0.00124 | 0.983 | -0.00011 | 1 | Non-significant |
| Psoriasis vulgaris | ebi-a-GCST90001669 | -0.244 | -0.00014 | 0.00723 | 0.985 | 0.00057 | 1 | Non-significant |
| Type 1 diabetes | ebi-a-GCST90001669 | 0.23 | ####### | 0.0041 | 0.985 | -0.00034 | 1 | Non-significant |
| Type 1 diabetes | ebi-a-GCST90001933 | 0.23 | 2.80E-05 | 0.00147 | 0.985 | 0.000122 | 1 | Non-significant |
| Psoriasis vulgaris | ebi-a-GCST90001512 | -0.244 | 7.14E-05 | 0.00376 | 0.985 | -0.00029 | 1 | Non-significant |
| Psoriasis vulgaris | ebi-a-GCST90001944 | -0.244 | 0.000111 | 0.00591 | 0.985 | -0.00046 | 1 | Non-significant |
| Type 1 diabetes | ebi-a-GCST90001951 | 0.23 | 5.06E-05 | 0.00274 | 0.985 | 0.00022 | 1 | Non-significant |
| Psoriasis vulgaris | ebi-a-GCST90001941 | -0.244 | 0.000138 | 0.00757 | 0.985 | -0.00057 | 1 | Non-significant |
| Psoriasis vulgaris | ebi-a-GCST90001461 | -0.244 | -0.00022 | 0.0124 | 0.986 | 0.000908 | 1 | Non-significant |
| Type 1 diabetes | ebi-a-GCST90002030 | 0.23 | ####### | 0.00278 | 0.986 | -0.00021 | 1 | Non-significant |
| Type 1 diabetes | ebi-a-GCST90001671 | 0.23 | ####### | 0.00483 | 0.986 | -0.00036 | 1 | Non-significant |
| Psoriasis vulgaris | ebi-a-GCST90001671 | -0.244 | ####### | 0.00395 | 0.986 | 0.000276 | 1 | Non-significant |
| Type 1 diabetes | ebi-a-GCST90001465 | 0.23 | 0.000161 | 0.0101 | 0.987 | 0.000702 | 1 | Non-significant |
| Psoriasis vulgaris | ebi-a-GCST90002013 | -0.244 | -0.00022 | 0.0143 | 0.988 | 0.000918 | 1 | Non-significant |
| Type 1 diabetes | ebi-a-GCST90002013 | 0.23 | 0.00015 | 0.0096 | 0.988 | 0.000652 | 1 | Non-significant |
| Psoriasis vulgaris | ebi-a-GCST90001834 | -0.244 | 0.000222 | 0.0146 | 0.988 | -0.00091 | 1 | Non-significant |
| Psoriasis vulgaris | ebi-a-GCST90001697 | -0.244 | 6.88E-07 | 4.84E-05 | 0.989 | ####### | 1 | Non-significant |
| Type 1 diabetes | ebi-a-GCST90001532 | 0.23 | ####### | 0.00392 | 0.989 | -0.00023 | 1 | Non-significant |
| Type 1 diabetes | ebi-a-GCST90001811 | 0.23 | 0.000173 | 0.0151 | 0.991 | 0.000751 | 1 | Non-significant |
| Psoriasis vulgaris | ebi-a-GCST90001811 | -0.244 | 3.41E-05 | 0.003 | 0.991 | -0.00014 | 1 | Non-significant |
| Psoriasis vulgaris | ebi-a-GCST90001951 | -0.244 | 1.42E-06 | 0.000125 | 0.991 | ####### | 1 | Non-significant |
| Type 1 diabetes | ebi-a-GCST90001517 | 0.23 | 1.86E-05 | 0.00167 | 0.991 | 8.10E-05 | 1 | Non-significant |
| Type 1 diabetes | ebi-a-GCST90001658 | 0.23 | ####### | 0.00316 | 0.992 | -0.00014 | 1 | Non-significant |
| Type 1 diabetes | ebi-a-GCST90002103 | 0.23 | -0.00053 | 0.0526 | 0.992 | -0.00229 | 1 | Non-significant |
| Type 1 diabetes | ebi-a-GCST90001884 | 0.23 | ####### | 0.00638 | 0.992 | -0.00028 | 1 | Non-significant |
| Psoriasis vulgaris | ebi-a-GCST90001884 | -0.244 | 1.33E-05 | 0.00134 | 0.992 | ####### | 1 | Non-significant |
| Type 1 diabetes | ebi-a-GCST90002005 | 0.23 | ####### | 0.00842 | 0.993 | -0.00033 | 1 | Non-significant |
| Psoriasis vulgaris | ebi-a-GCST90001945 | -0.244 | 5.37E-05 | 0.00605 | 0.993 | -0.00022 | 1 | Non-significant |
| Psoriasis vulgaris | ebi-a-GCST90001751 | -0.244 | -0.00022 | 0.0262 | 0.993 | 0.000889 | 1 | Non-significant |
| Type 1 diabetes | ebi-a-GCST90001984 | 0.23 | 7.78E-05 | 0.00951 | 0.993 | 0.000339 | 1 | Non-significant |
| Type 1 diabetes | ebi-a-GCST90001621 | 0.23 | ####### | 0.00134 | 0.994 | ####### | 1 | Non-significant |
| Type 1 diabetes | ebi-a-GCST90001515 | 0.23 | ####### | 0.00532 | 0.995 | -0.00016 | 1 | Non-significant |
| Psoriasis vulgaris | ebi-a-GCST90001515 | -0.244 | 3.50E-05 | 0.00514 | 0.995 | -0.00014 | 1 | Non-significant |
| Type 1 diabetes | ebi-a-GCST90001916 | 0.23 | 6.69E-05 | 0.0112 | 0.995 | 0.000291 | 1 | Non-significant |
| Type 1 diabetes | ebi-a-GCST90001574 | 0.23 | 5.72E-05 | 0.0096 | 0.995 | 0.000249 | 1 | Non-significant |
| Psoriasis vulgaris | ebi-a-GCST90001900 | -0.244 | ####### | 0.00285 | 0.996 | 6.47E-05 | 1 | Non-significant |
| Psoriasis vulgaris | ebi-a-GCST90001920 | -0.244 | ####### | 0.0182 | 0.996 | 0.000406 | 1 | Non-significant |
| Type 1 diabetes | ebi-a-GCST90001920 | 0.23 | 7.72E-05 | 0.0142 | 0.996 | 0.000336 | 1 | Non-significant |
| Type 1 diabetes | ebi-a-GCST90001480 | 0.23 | 5.23E-05 | 0.0101 | 0.996 | 0.000228 | 1 | Non-significant |
| Type 1 diabetes | ebi-a-GCST90001577 | 0.23 | 8.69E-05 | 0.018 | 0.996 | 0.000378 | 1 | Non-significant |
| Type 1 diabetes | ebi-a-GCST90001960 | 0.23 | ####### | 0.0085 | 0.997 | -0.00014 | 1 | Non-significant |
| Type 1 diabetes | ebi-a-GCST90001954 | 0.23 | ####### | 0.00193 | 0.997 | ####### | 1 | Non-significant |
| Type 1 diabetes | ebi-a-GCST90002119 | 0.23 | ####### | 0.0101 | 0.998 | -0.00014 | 1 | Non-significant |
| Psoriasis vulgaris | ebi-a-GCST90002119 | -0.244 | 7.22E-06 | 0.00234 | 0.998 | ####### | 1 | Non-significant |
| Psoriasis vulgaris | ebi-a-GCST90001985 | -0.244 | ####### | 0.00776 | 0.998 | 9.55E-05 | 1 | Non-significant |
| Psoriasis vulgaris | ebi-a-GCST90001758 | -0.244 | 5.23E-05 | 0.0202 | 0.998 | -0.00021 | 1 | Non-significant |
| Psoriasis vulgaris | ebi-a-GCST90001552 | -0.244 | ####### | 0.0168 | 0.998 | 0.000163 | 1 | Non-significant |
| Type 1 diabetes | ebi-a-GCST90001919 | 0.23 | ####### | 0.0243 | 0.999 | -0.00012 | 1 | Non-significant |
| Type 1 diabetes | ebi-a-GCST90001979 | 0.23 | 4.78E-06 | 0.00514 | 0.999 | 2.08E-05 | 1 | Non-significant |
| Psoriasis vulgaris | ebi-a-GCST90001782 | -0.244 | ####### | 0.00329 | 1 | 8.20E-06 | 1 | Non-significant |
| Type 1 diabetes | ebi-a-GCST90002010 | 0.23 | 4.88E-06 | 0.0406 | 1 | 2.12E-05 | 1 | Non-significant |

Supplementary Table S4: High-throughput two-step Mendelian randomization screening for 731 circulating immune traits as potential mediators.
