# Supplementary material for: Association between Guillain–Barré syndrome and 7 autoimmune diseases: a mendelian randomization study
Source: BMC Neurol. 2026 May 9;26:425. doi: 10.1186/s12883-026-04957-8 (PMC13326399; doi:10.1186/s12883-026-04957-8)
Supplement: Supplementary file 5 — Supplementary Material 5. Supplementary Figures. [file 12883_2026_4957_MOESM5_ESM.pdf]

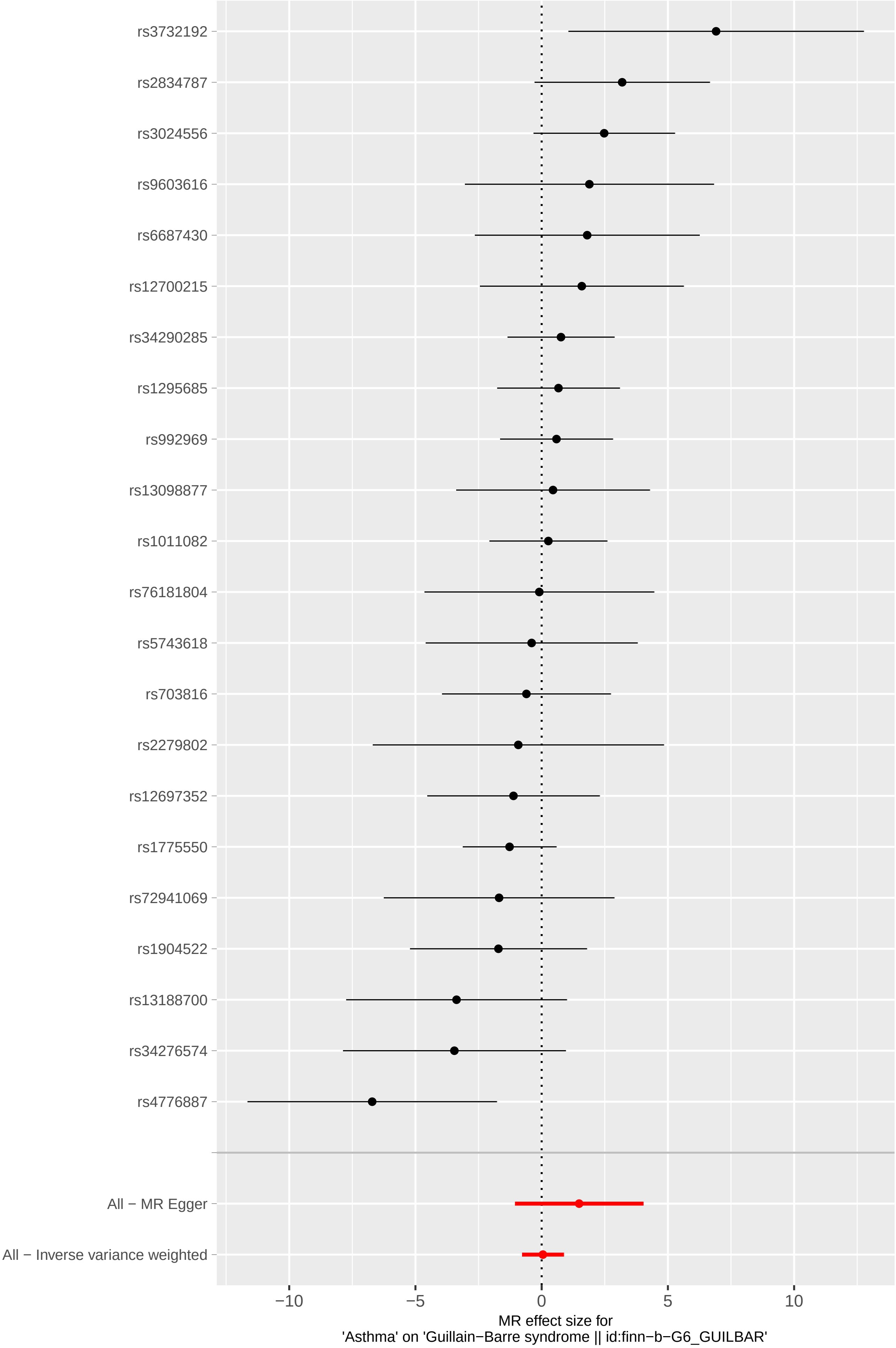

rs3732192
rs2834787
rs3024556
rs9603616
rs6687430
rs12700215
rs34290285
rs1295685
rs992969
rs13098877
rs1011082
rs76181804
rs5743618
rs703816
rs2279802
rs12697352
rs1775550
rs72941069
rs1904522
rs13188700
rs34276574
rs4776887
All − MR Egger
All − Inverse variance weighted
−10
−5
0
5
10
MR effect size for
'Asthma' on 'Guillain−Barre syndrome || id:finn−b−G6_GUILBAR'

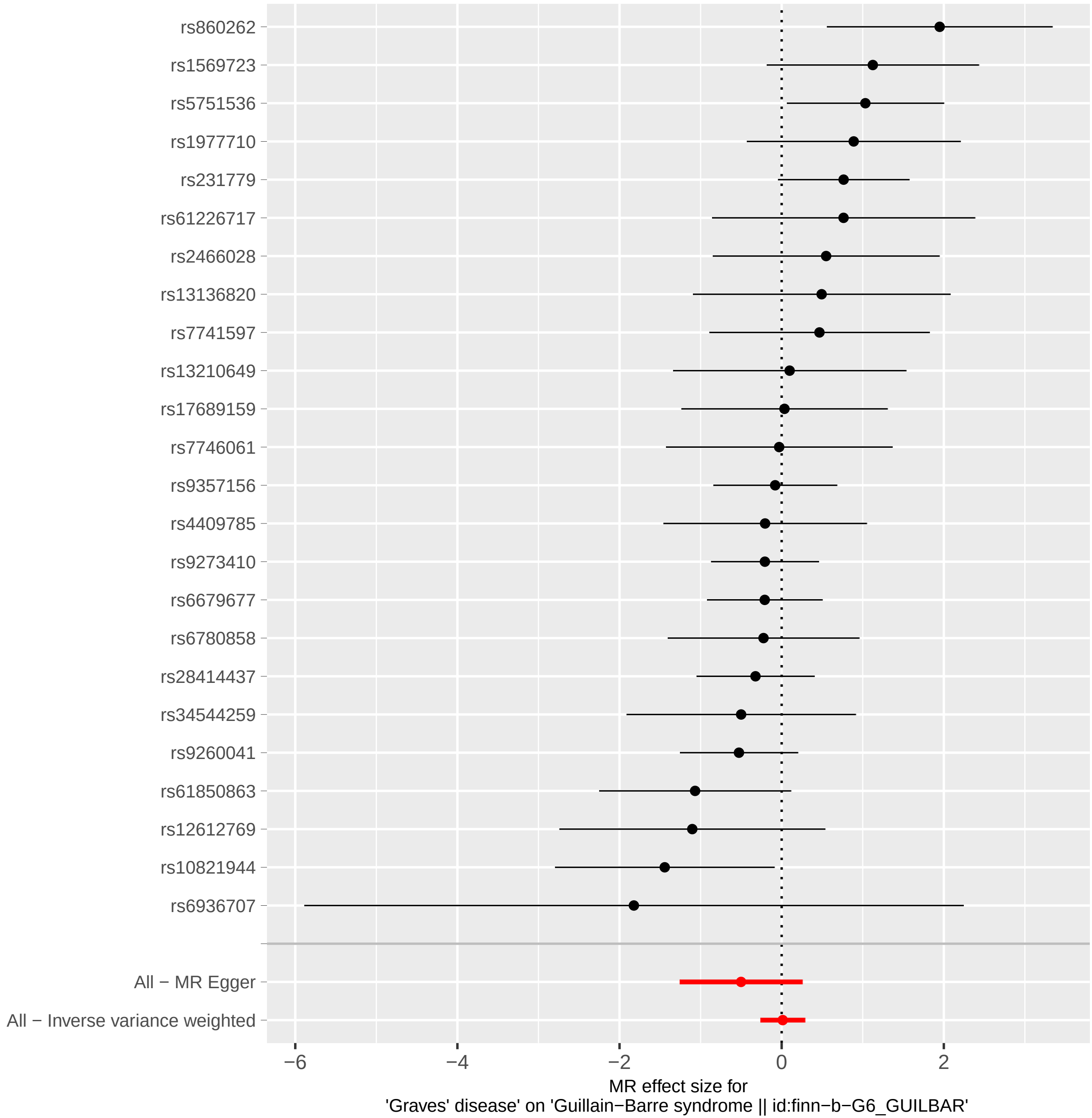
rs860262
rs1569723
rs5751536
rs1977710
rs231779
rs61226717
rs2466028
rs13136820
rs7741597
rs13210649
rs17689159
rs7746061
rs9357156
rs4409785
rs9273410
rs6679677
rs6780858
rs28414437
rs34544259
rs9260041
rs61850863
rs12612769
rs10821944
rs6936707
All − MR Egger
All − Inverse variance weighted
−6
−4
−2
0
2
MR effect size for
'Graves' disease' on 'Guillain−Barre syndrome || id:finn−b−G6_GUILBAR'

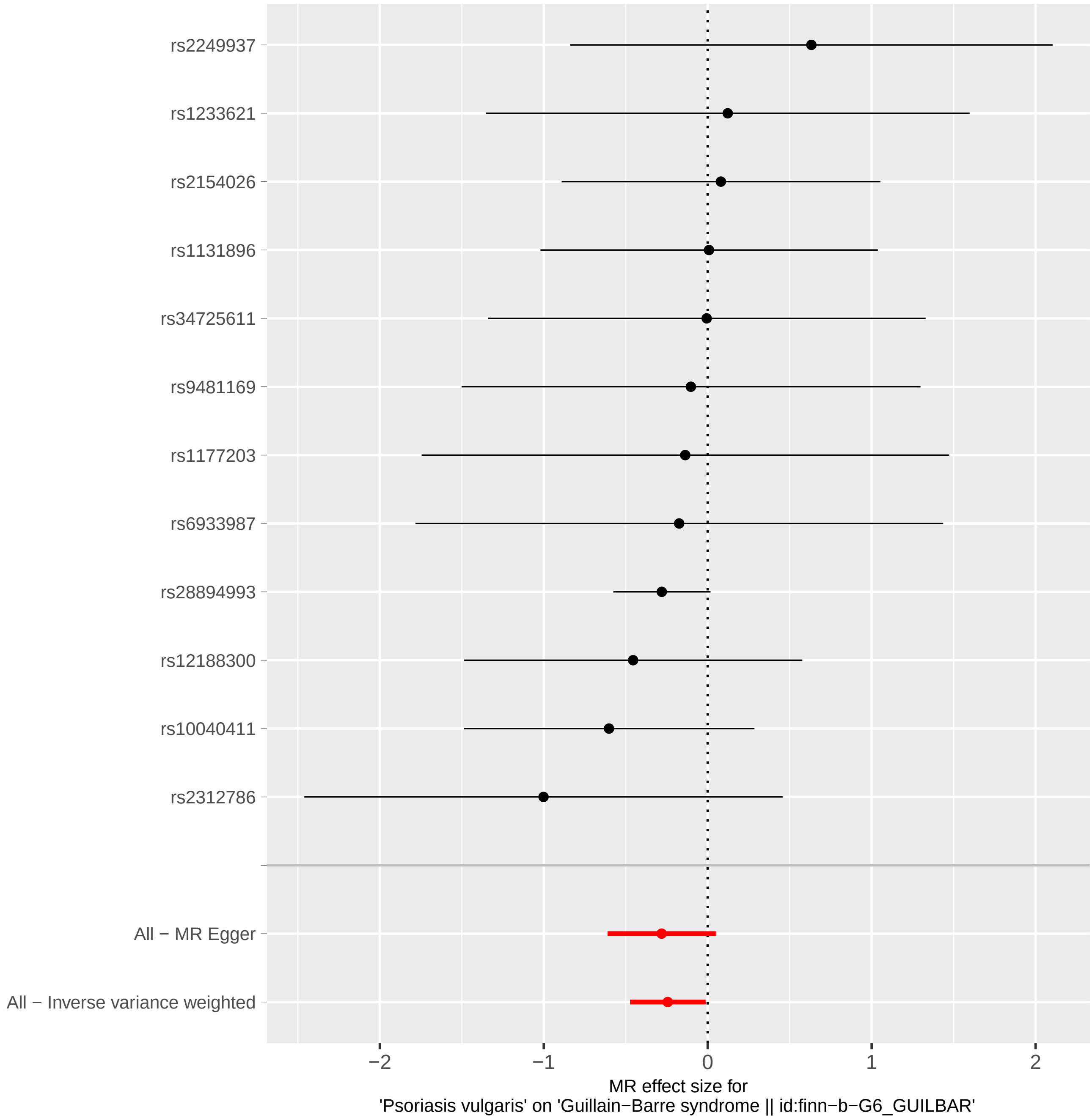
rs2249937
rs1233621
rs2154026
rs1131896
rs34725611
rs9481169
rs1177203
rs6933987
rs28894993
rs12188300
rs10040411
rs2312786
All − MR Egger
All − Inverse variance weighted
−2
−1
0
1
2
MR effect size for
'Psoriasis vulgaris' on 'Guillain−Barre syndrome || id:finn−b−G6_GUILBAR'

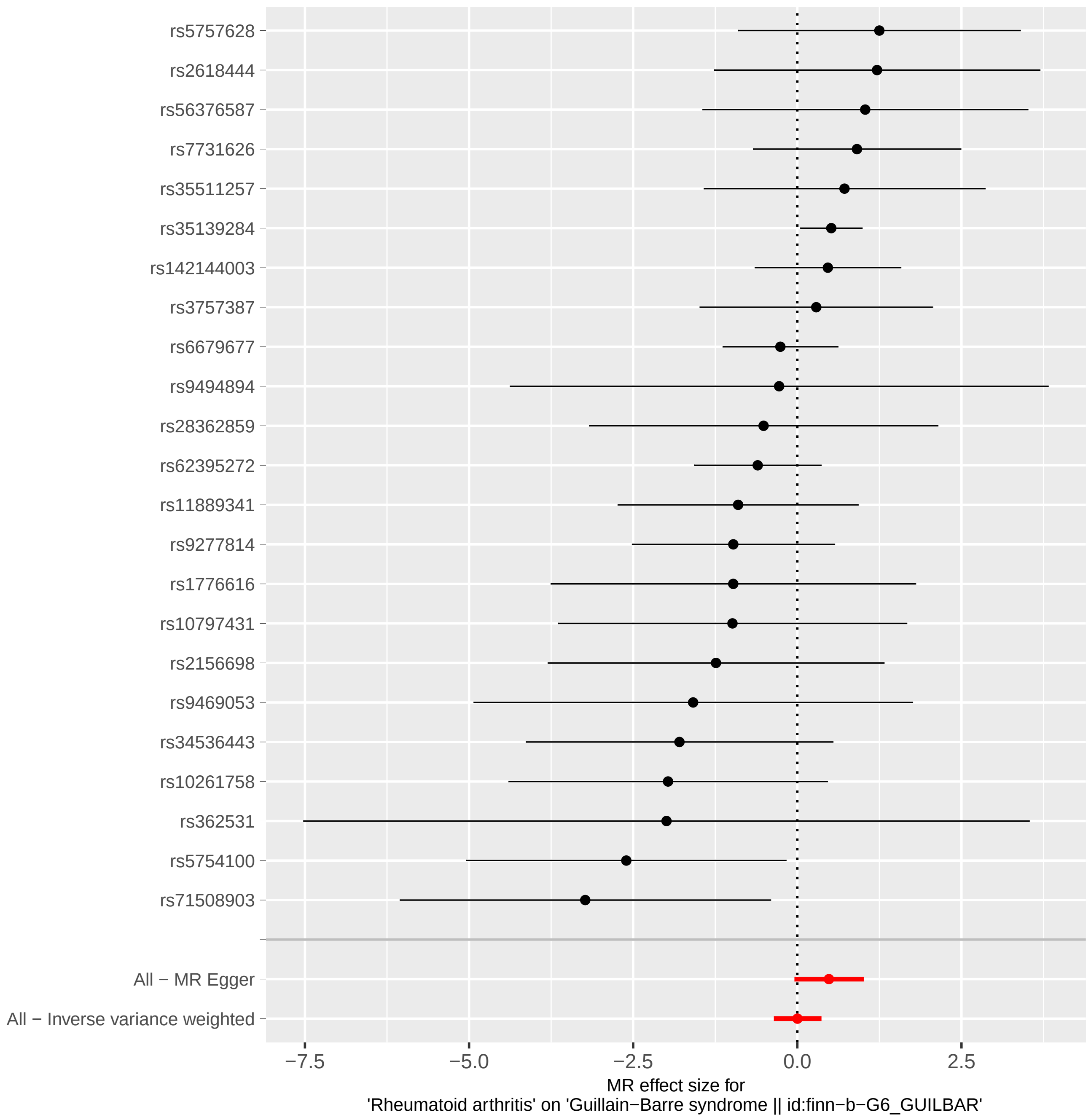
rs5757628
rs2618444
rs56376587
rs7731626
rs35511257
rs35139284
rs142144003
rs3757387
rs6679677
rs9494894
rs28362859
rs62395272
rs11889341
rs9277814
rs1776616
rs10797431
rs2156698
rs9469053
rs34536443
rs10261758
rs362531
rs5754100
rs71508903
All − MR Egger
All − Inverse variance weighted
−7.5
−5.0
−2.5
0.0
2.5
MR effect size for
'Rheumatoid arthritis' on 'Guillain−Barre syndrome || id:finn−b−G6_GUILBAR'

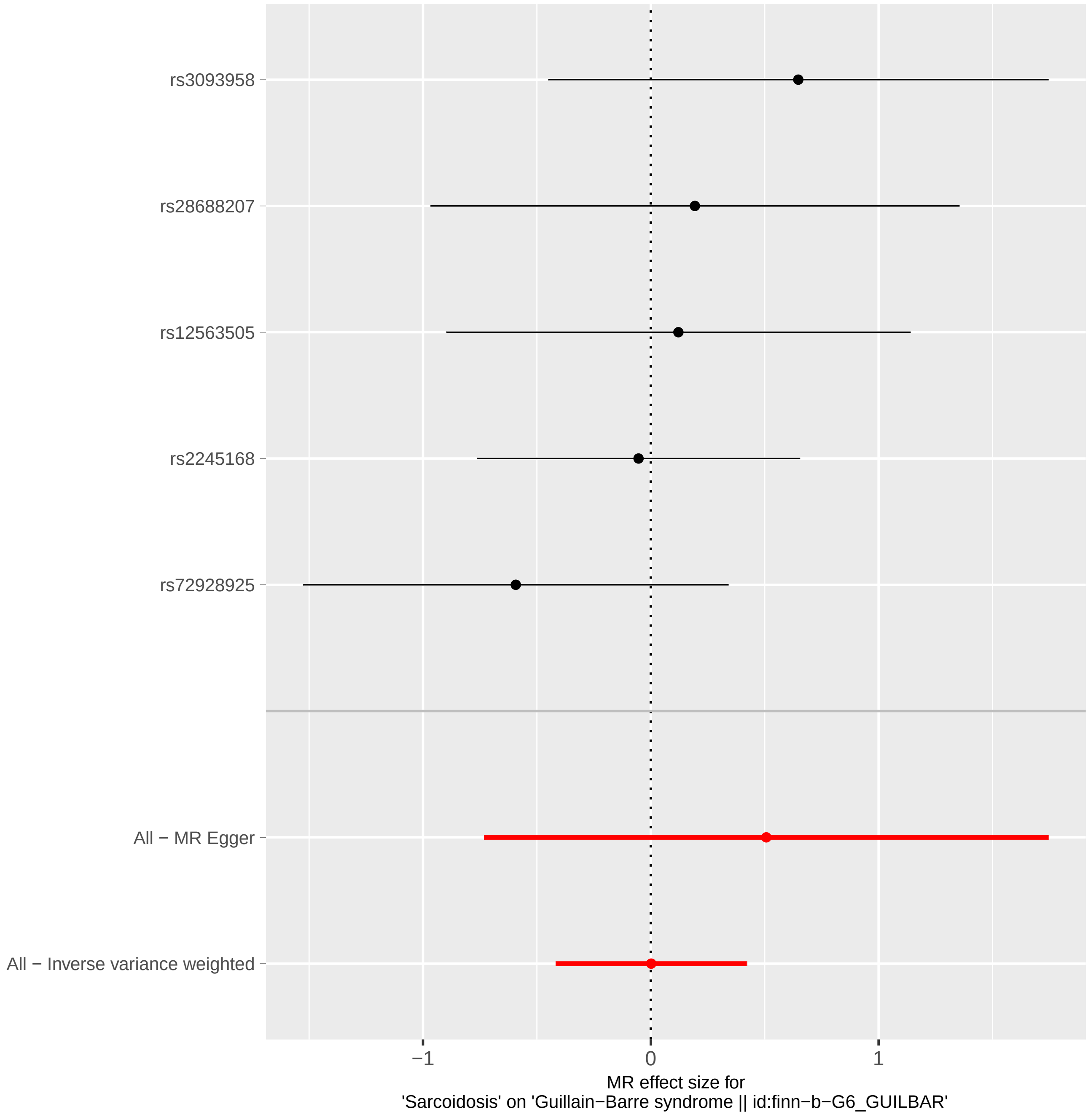
rs3093958
rs28688207
rs12563505
rs2245168
rs72928925
All − MR Egger
All − Inverse variance weighted
−1
0
1
MR effect size for
'Sarcoidosis' on 'Guillain−Barre syndrome || id:finn−b−G6_GUILBAR'

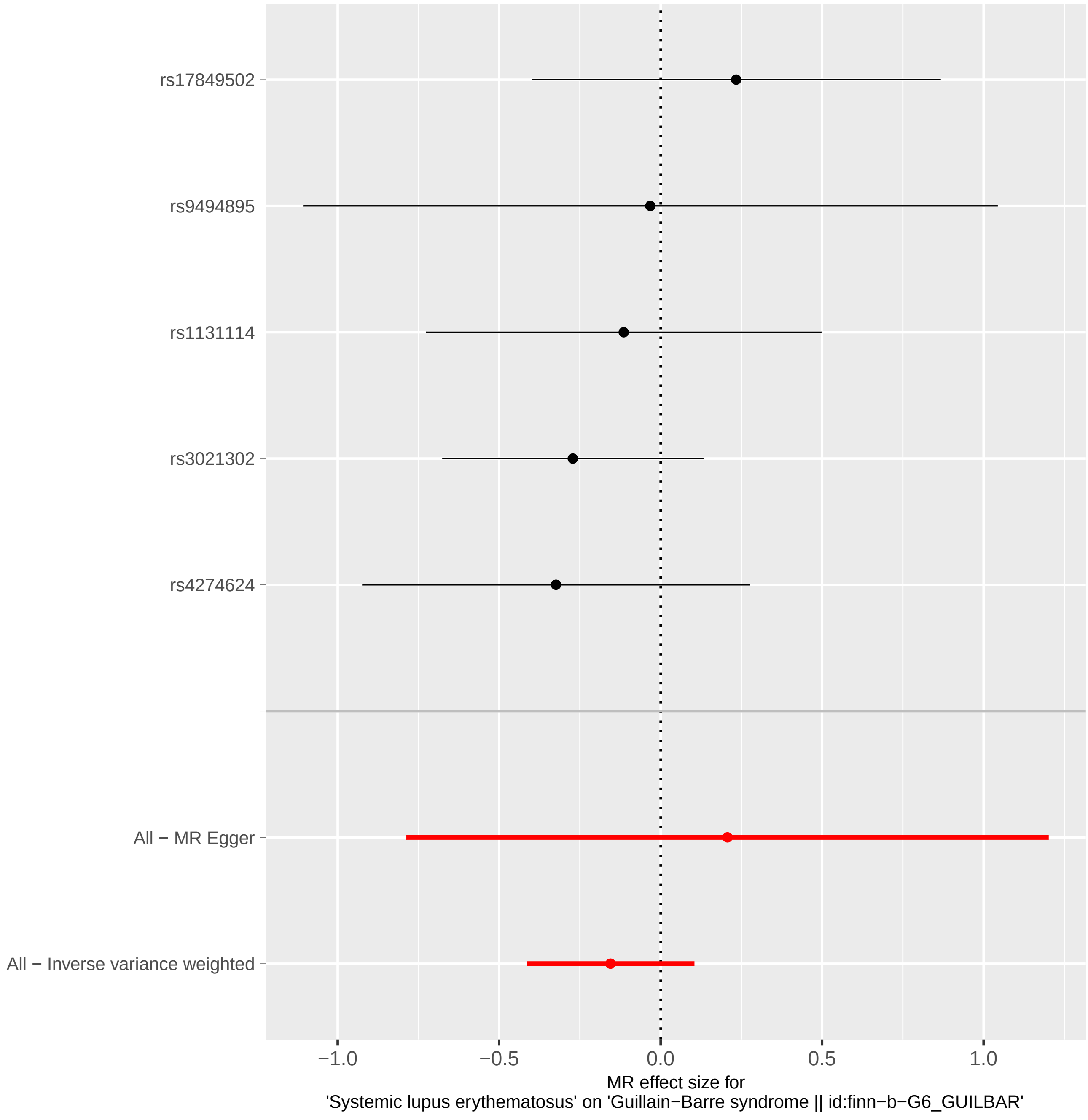

rs17849502
rs9494895
rs1131114
rs3021302
rs4274624
All − MR Egger
All − Inverse variance weighted
−1.0
−0.5
0.0
0.5
1.0
MR effect size for
'Systemic lupus erythematosus' on 'Guillain−Barre syndrome || id:finn−b−G6_GUILBAR'

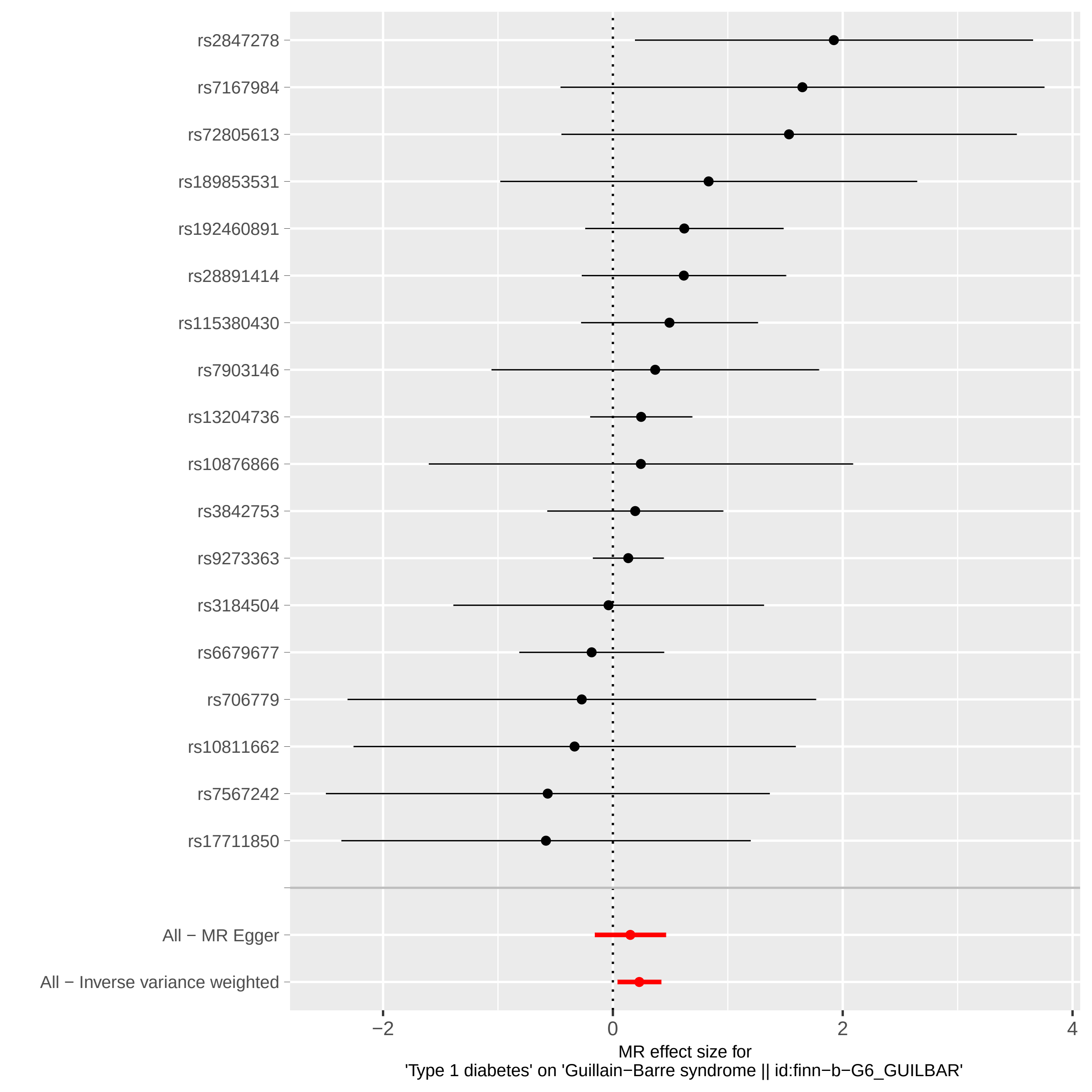

rs2847278
rs7167984
rs72805613
rs189853531
rs192460891
rs28891414
rs115380430
rs7903146
rs13204736
rs10876866
rs3842753
rs9273363
rs3184504
rs6679677
rs706779
rs10811662
rs7567242
rs17711850
All − MR Egger
All − Inverse variance weighted
−2
0
2
4
MR effect size for
'Type 1 diabetes' on 'Guillain−Barre syndrome || id:finn−b−G6_GUILBAR'

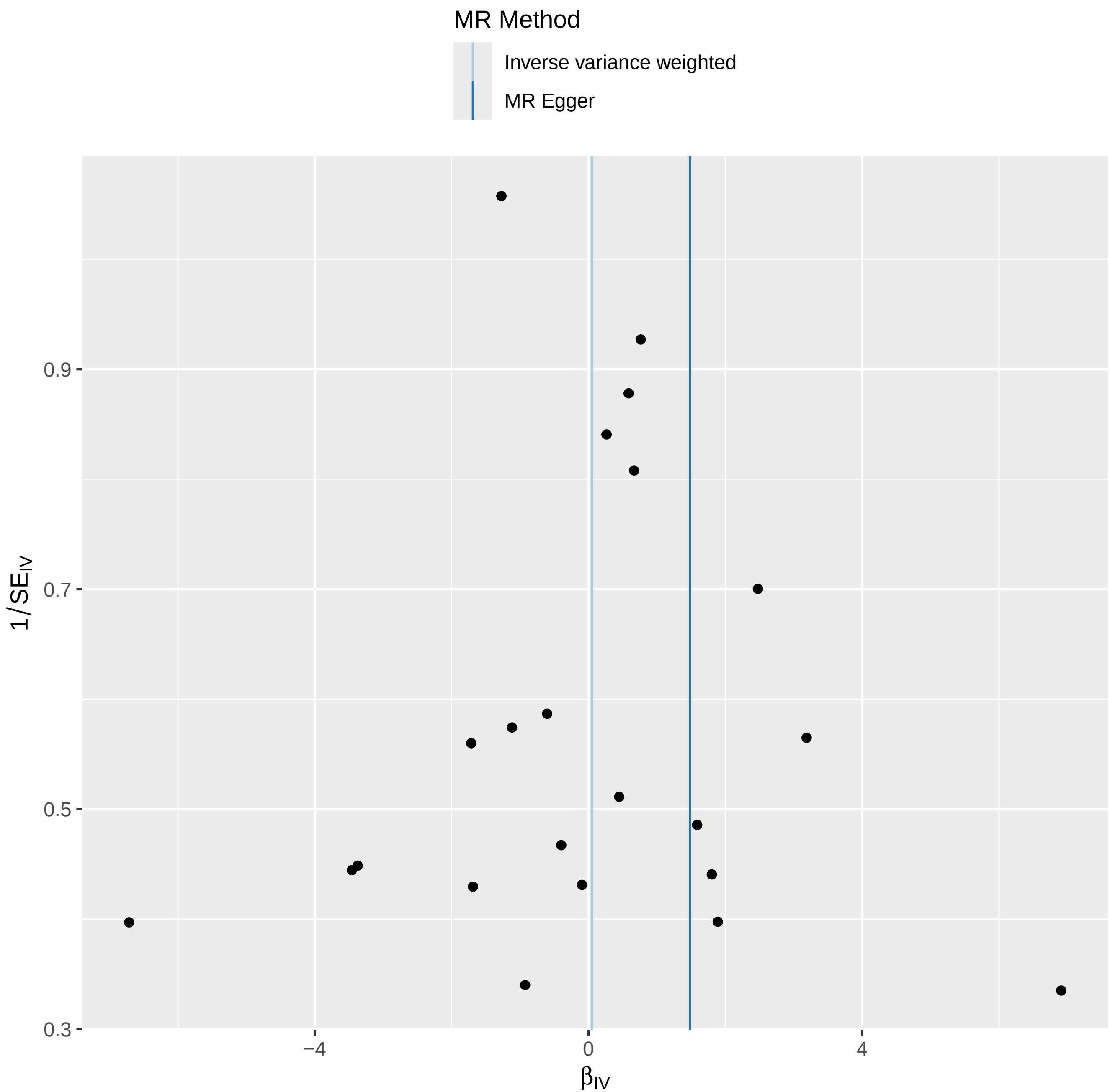
MR Method
Inverse variance weighted
MR Egger
0.9
0.7
0.5
0.3
−4
0
4
$1/SE_{IV}$
$\beta_{IV}$

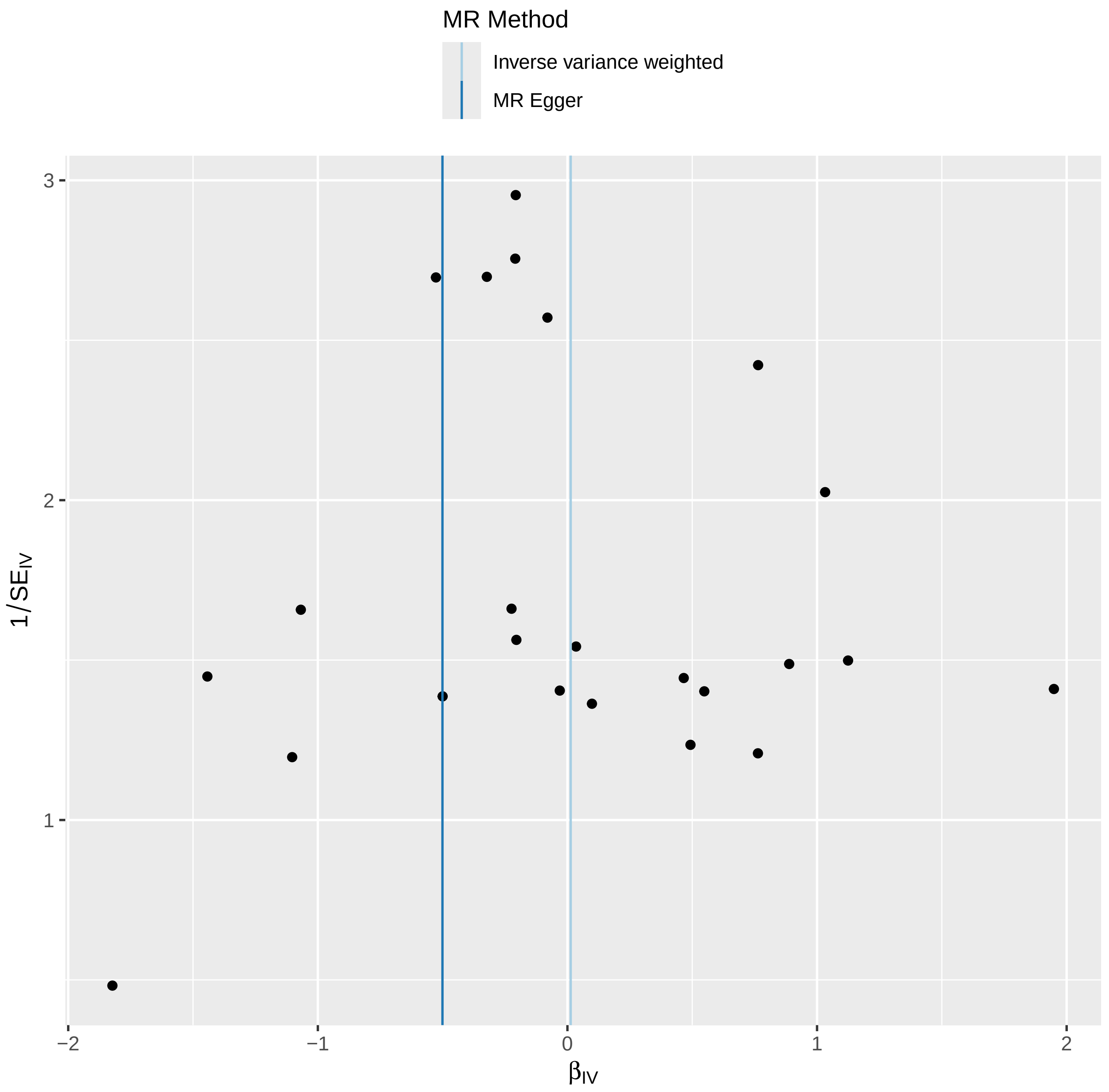

MR Method
Inverse variance weighted
MR Egger
3
2
1
$1/SE_{IV}$
−2
−1
0
1
2
$\beta_{IV}$

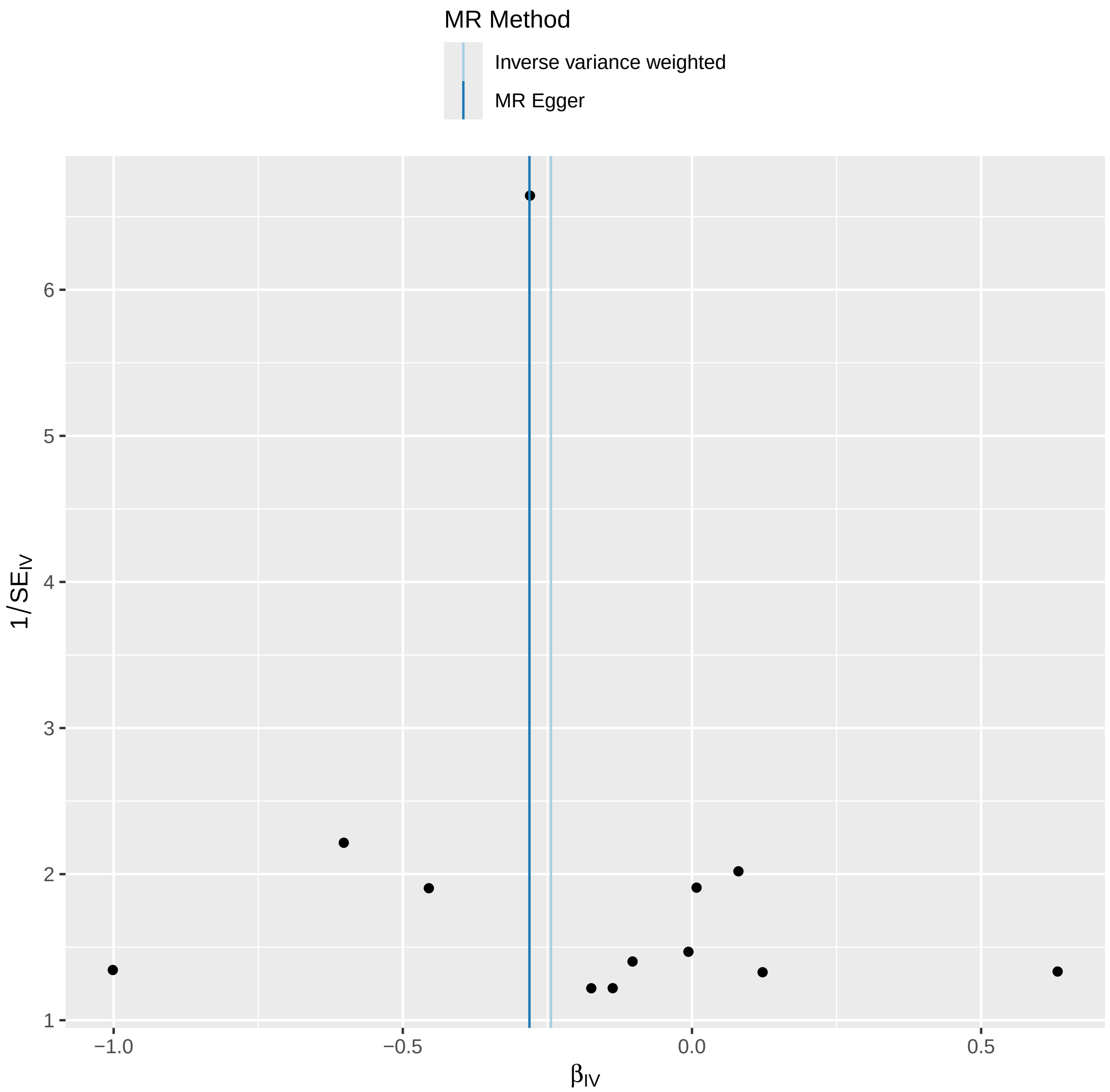
MR Method
Inverse variance weighted
MR Egger
1/SE_IV
1
2
3
4
5
6
−1.0
−0.5
0.0
0.5
β_IV

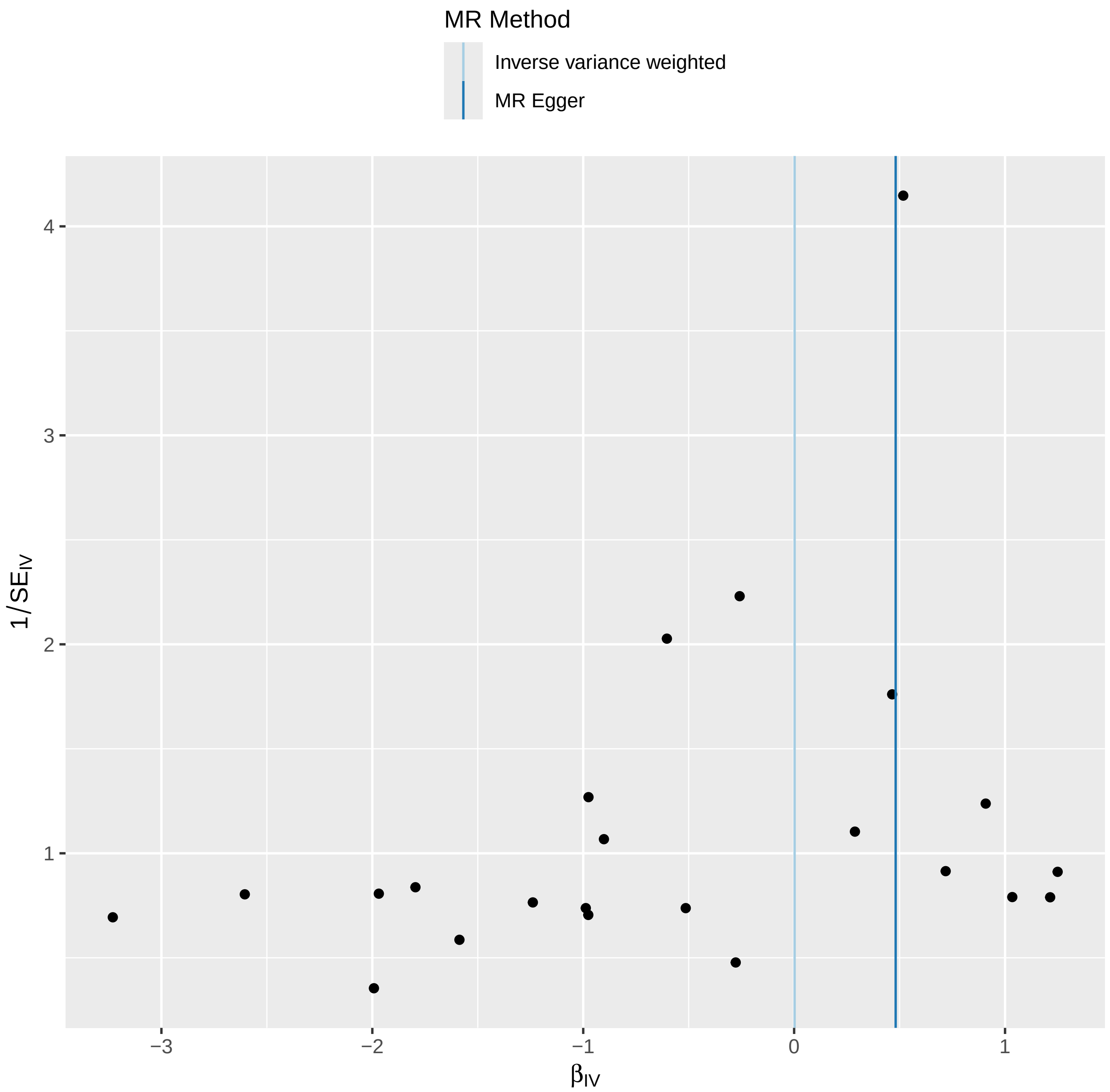
MR Method
Inverse variance weighted
MR Egger
1/SE_IV
4
3
2
1
−3
−2
−1
0
1
β_IV

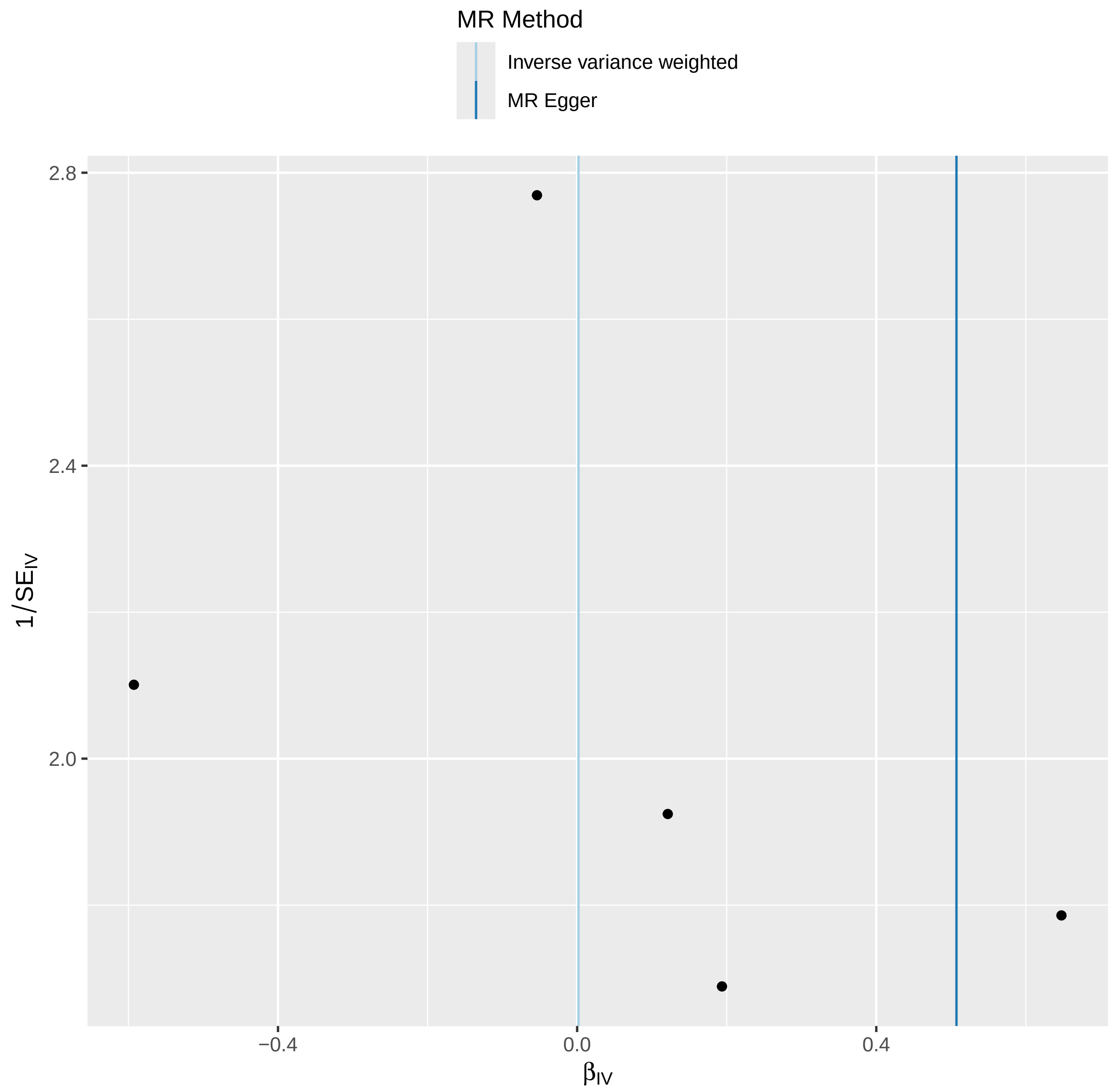
MR Method
Inverse variance weighted
MR Egger
2.8
2.4
2.0
1/SE_IV
−0.4
0.0
0.4
β_IV

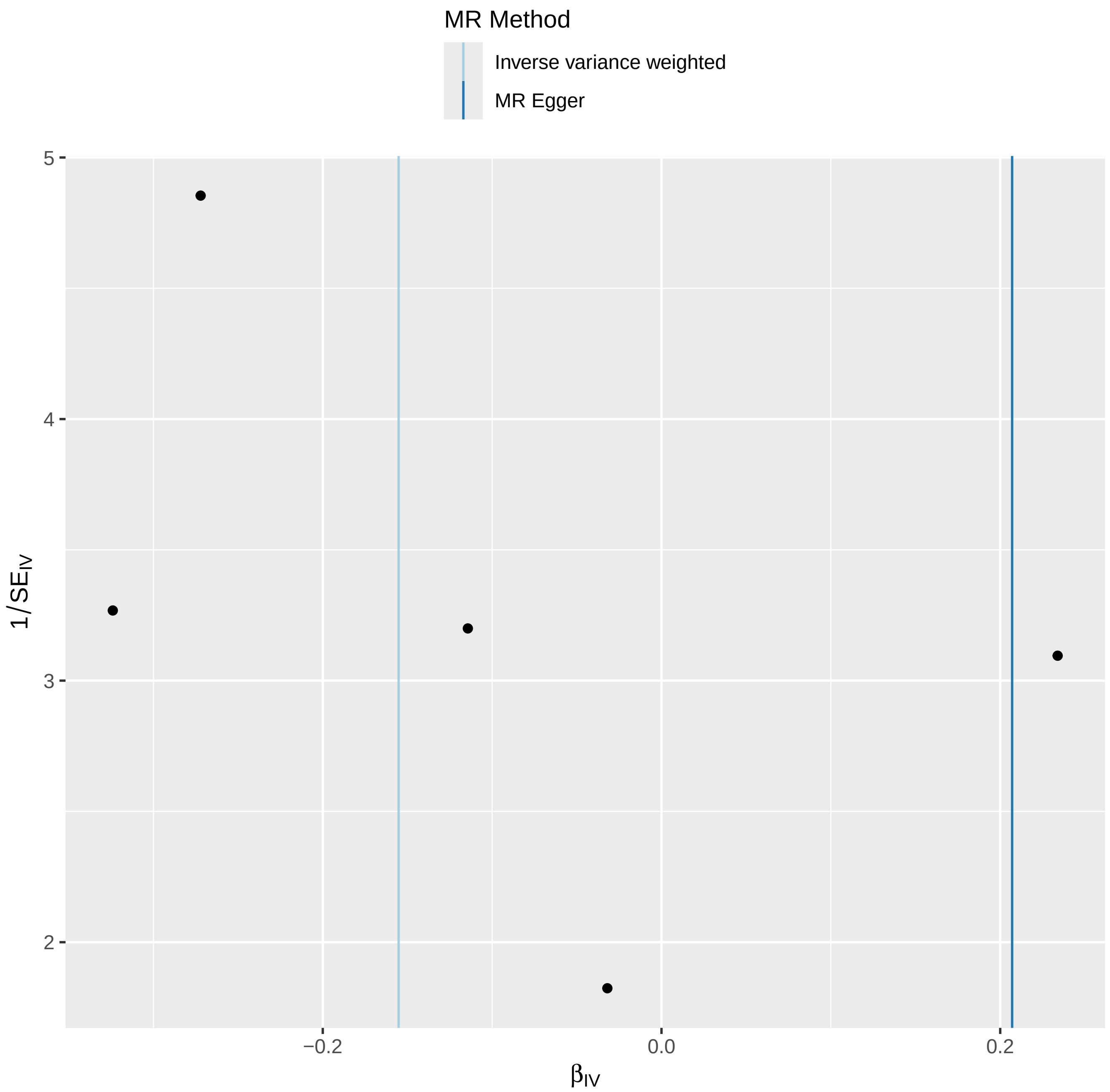

MR Method
Inverse variance weighted
MR Egger
5
4
3
2
−0.2
0.0
0.2
$1/SE_{IV}$
$\beta_{IV}$

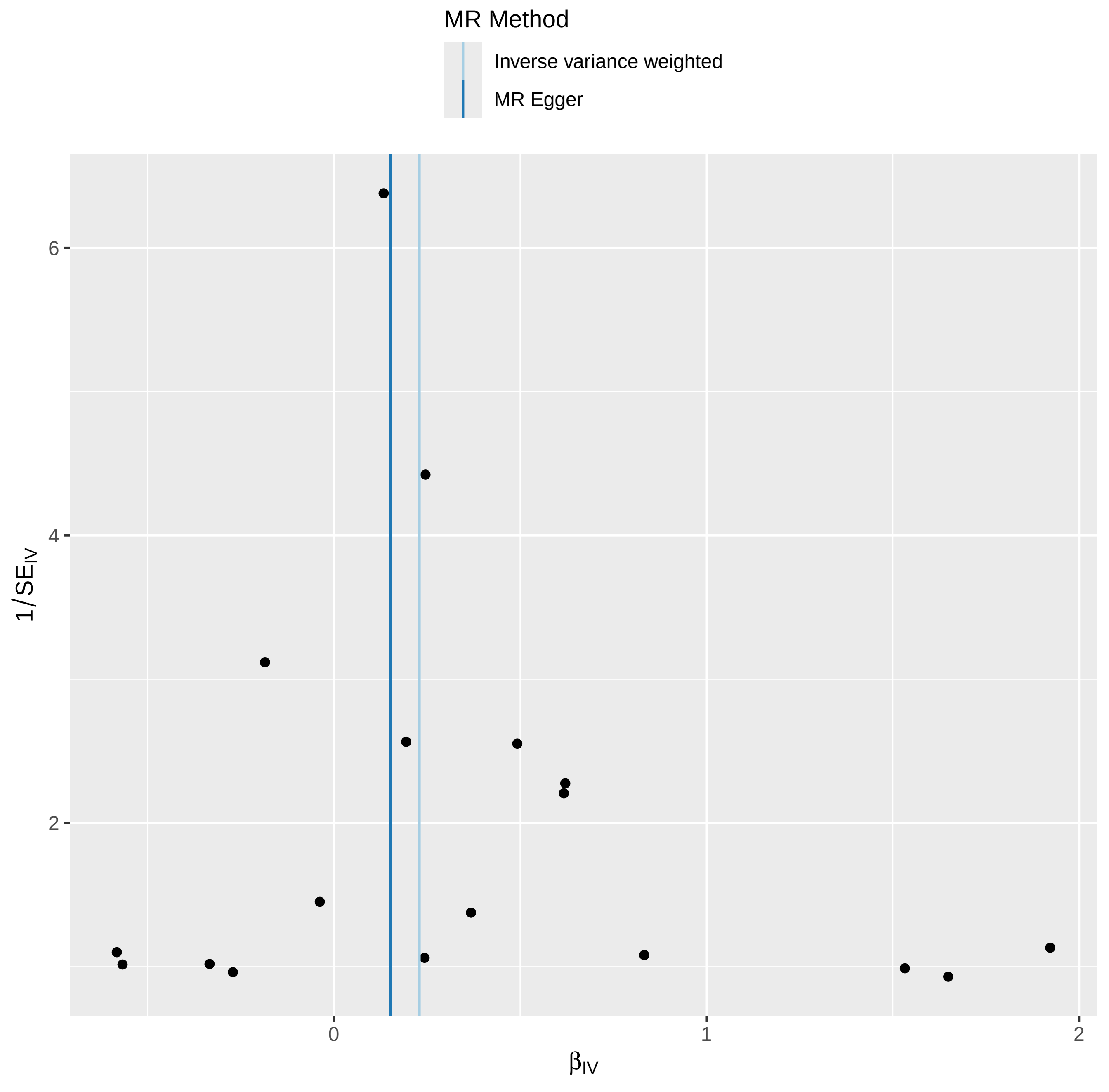

MR Method
Inverse variance weighted
MR Egger
6
4
2
1/SE_IV
0
1
2
β_IV

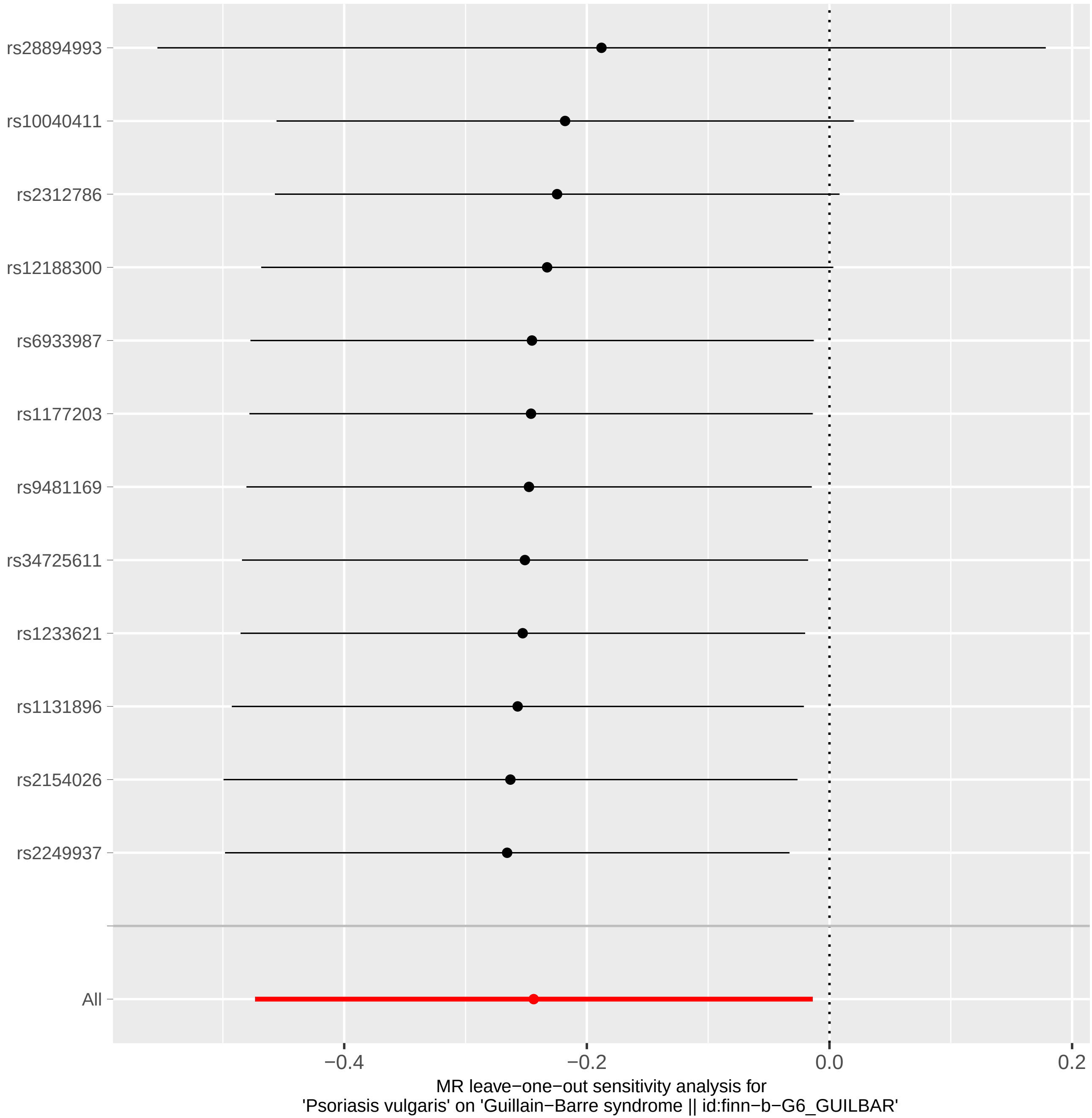
rs28894993
rs10040411
rs2312786
rs12188300
rs6933987
rs1177203
rs9481169
rs34725611
rs1233621
rs1131896
rs2154026
rs2249937
All
−0.4
−0.2
0.0
0.2
MR leave−one−out sensitivity analysis for
'Psoriasis vulgaris' on 'Guillain−Barre syndrome || id:finn−b−G6_GUILBAR'

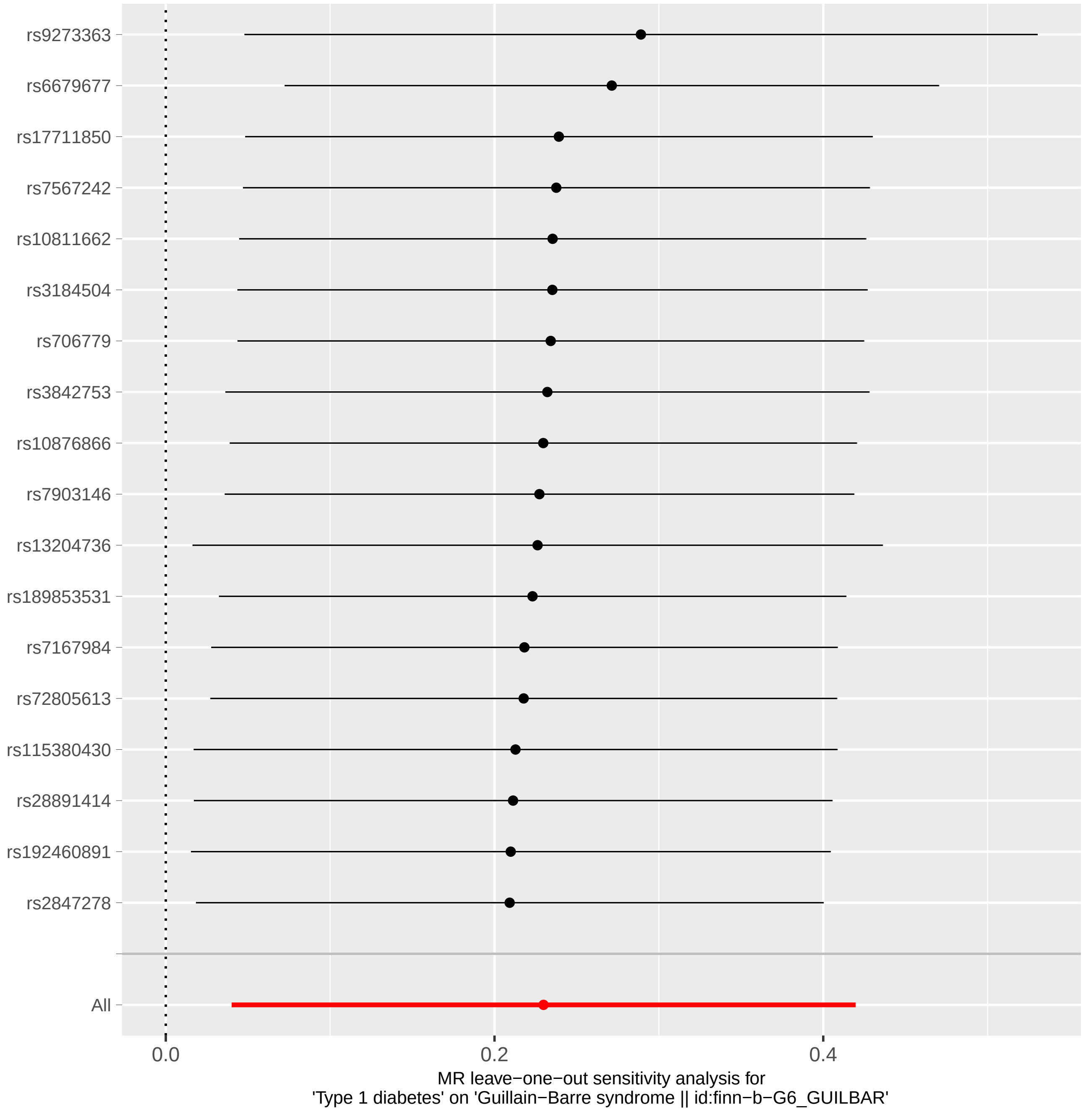
rs9273363
rs6679677
rs17711850
rs7567242
rs10811662
rs3184504
rs706779
rs3842753
rs10876866
rs7903146
rs13204736
rs189853531
rs7167984
rs72805613
rs115380430
rs28891414
rs192460891
rs2847278
All
0.0
0.2
0.4
MR leave−one−out sensitivity analysis for
'Type 1 diabetes' on 'Guillain−Barre syndrome || id:finn−b−G6_GUILBAR'

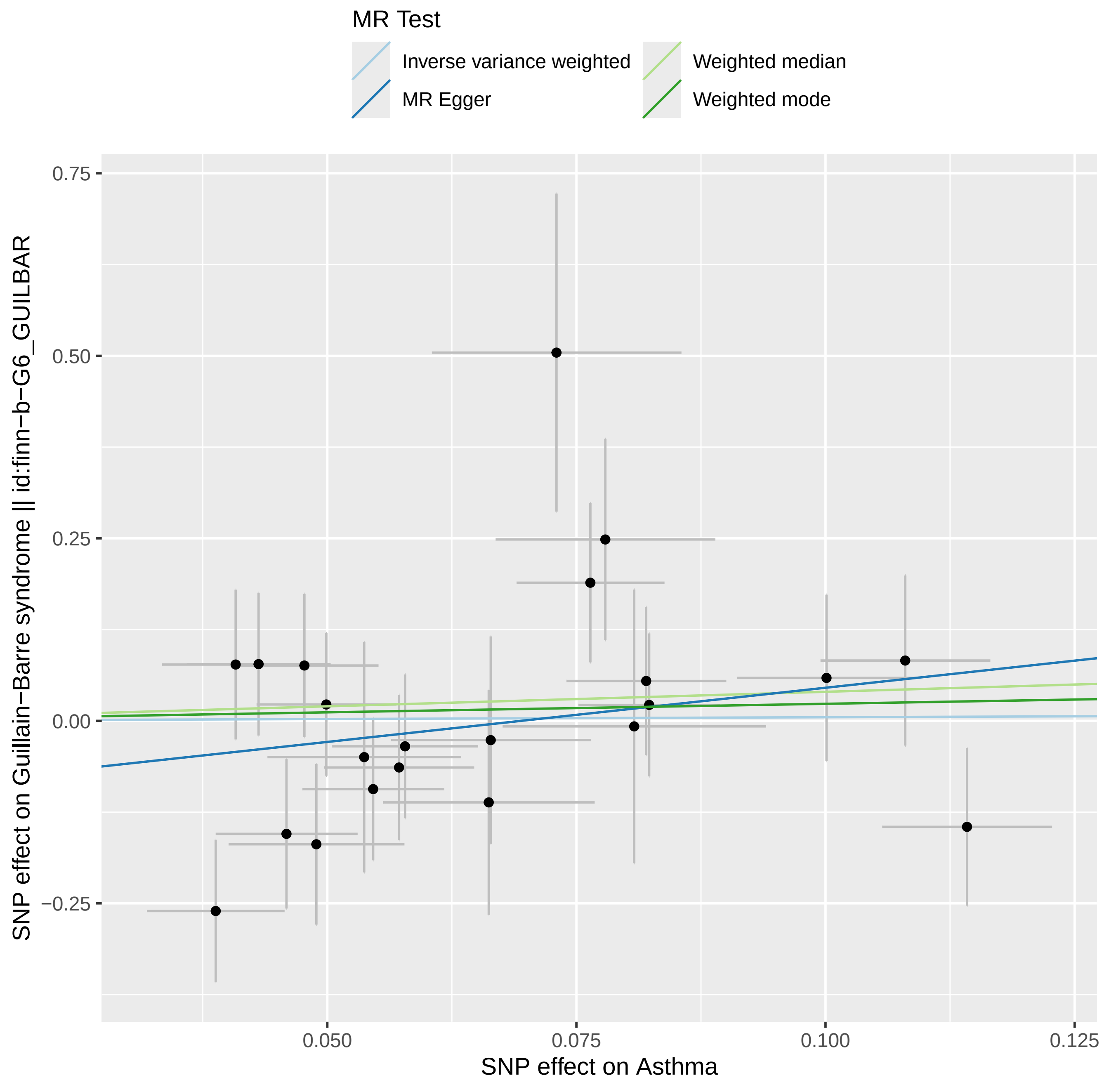
MR Test
Inverse variance weighted
MR Egger
Weighted median
Weighted mode
0.75
0.50
0.25
0.00
−0.25
0.050
0.075
0.100
0.125
SNP effect on Asthma
SNP effect on Guillain−Barre syndrome || id:finn−b−G6_GUILBAR

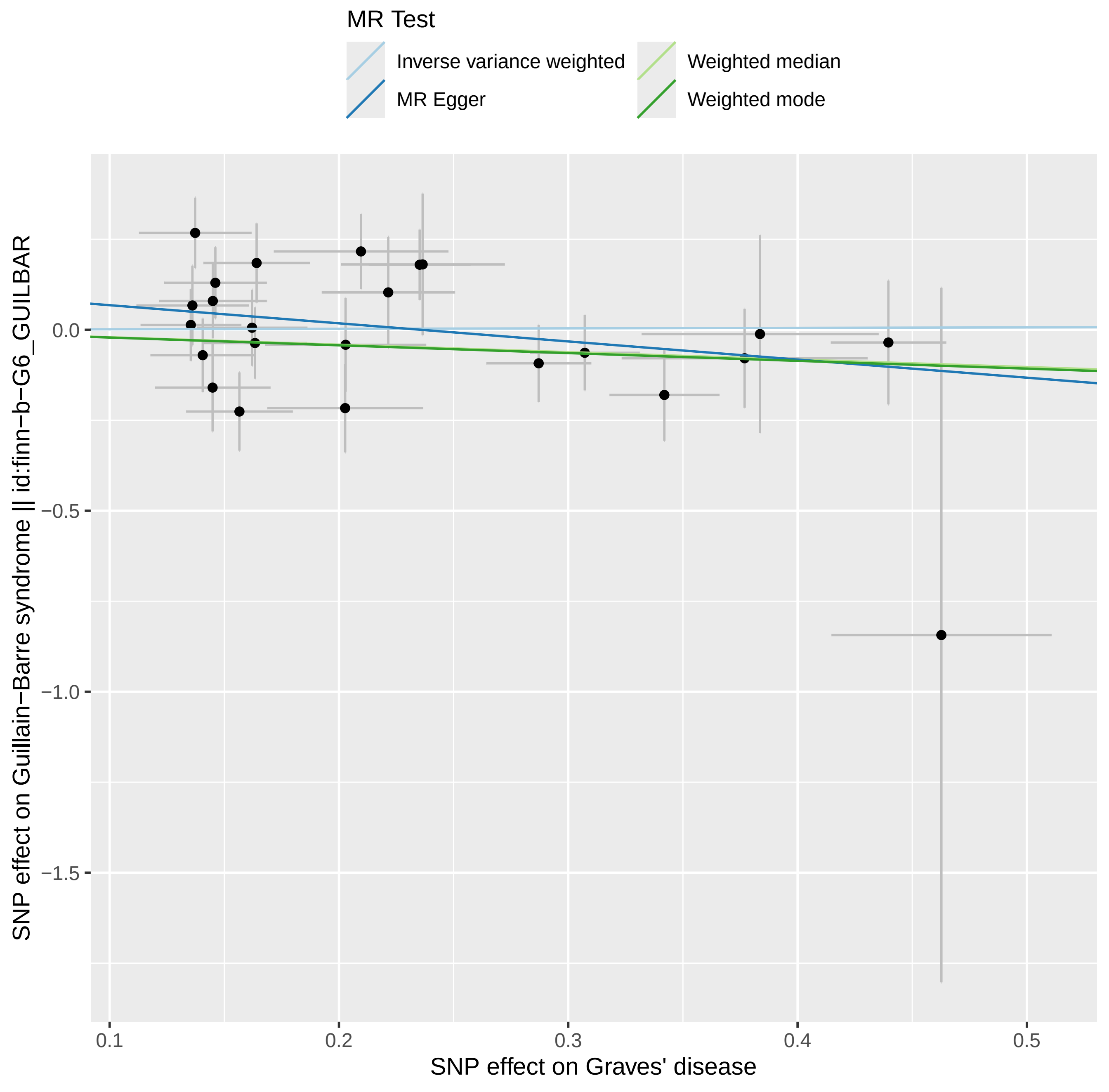
MR Test
Inverse variance weighted
MR Egger
Weighted median
Weighted mode
SNP effect on Guillain−Barre syndrome || id:finn-b-G6_GUILBAR
0.0
−0.5
−1.0
−1.5
0.1
0.2
0.3
0.4
0.5
SNP effect on Graves' disease

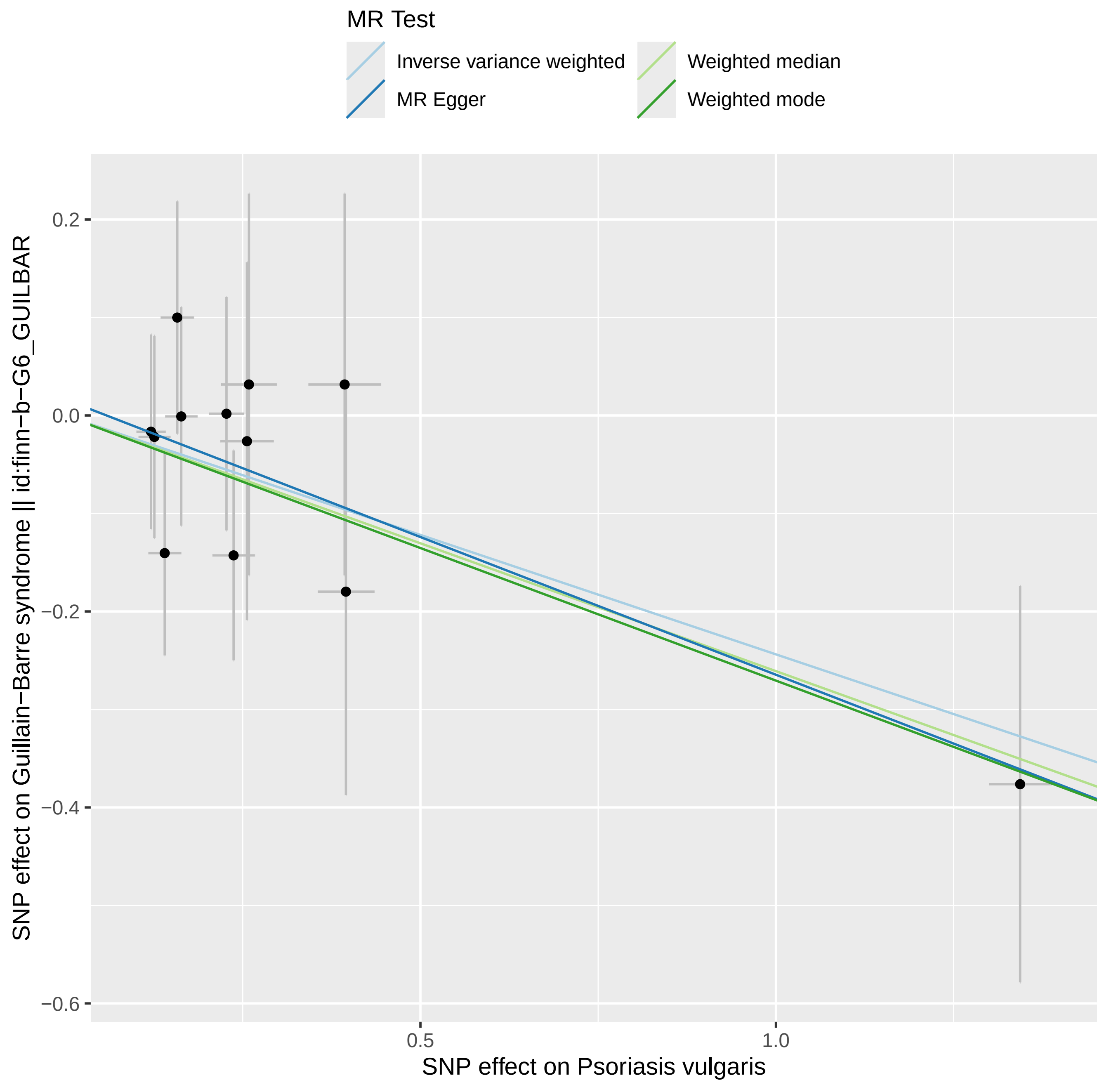
MR Test
Inverse variance weighted
MR Egger
Weighted median
Weighted mode
SNP effect on Guillain−Barre syndrome || id:finn−b−G6_GUILBAR
0.2
0.0
−0.2
−0.4
−0.6
0.5
1.0
SNP effect on Psoriasis vulgaris

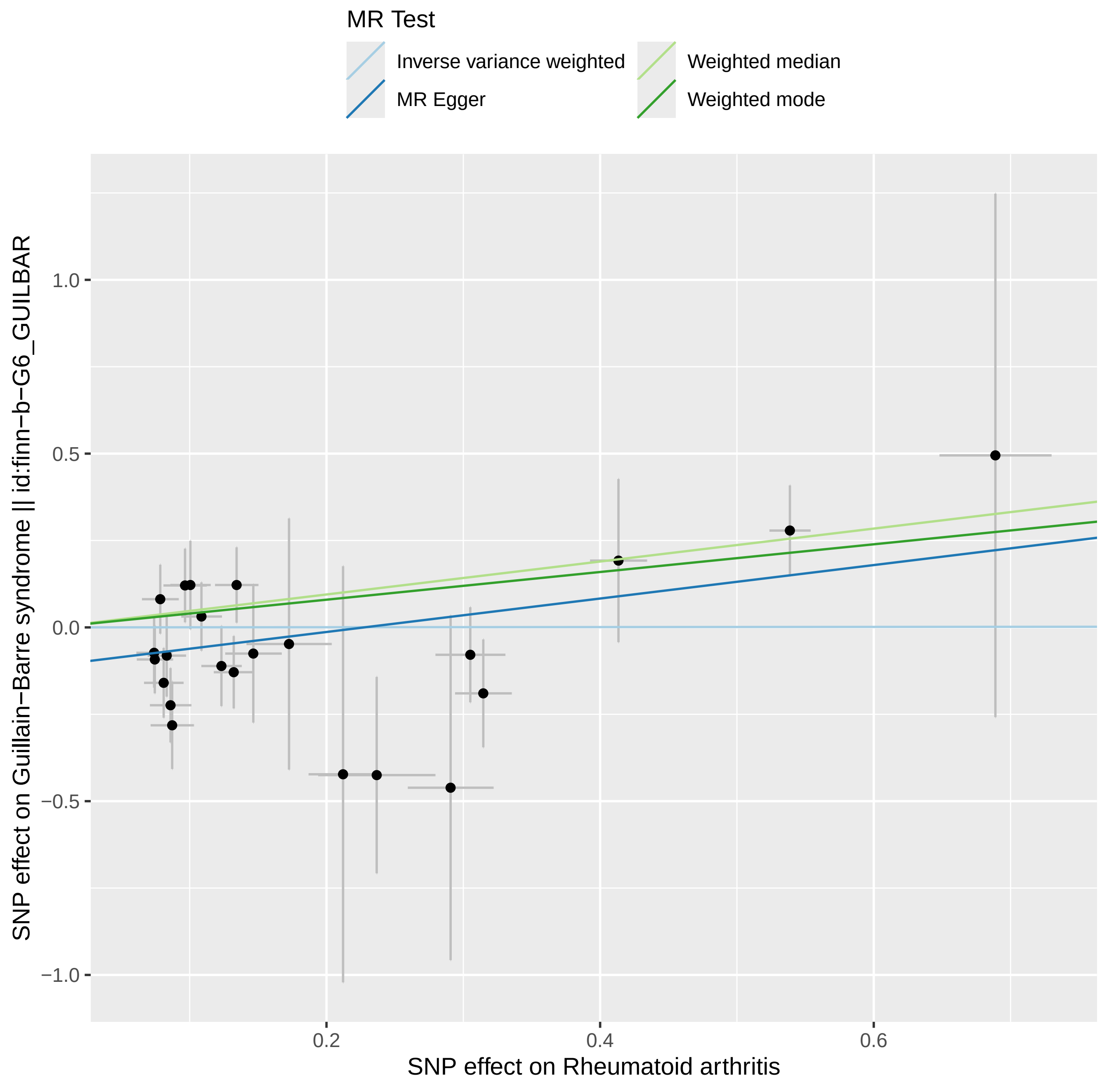
MR Test
Inverse variance weighted
MR Egger
Weighted median
Weighted mode
SNP effect on Guillain−Barre syndrome || id:finn−b−G6_GUILBAR
1.0
0.5
0.0
−0.5
−1.0
0.2
0.4
0.6
SNP effect on Rheumatoid arthritis

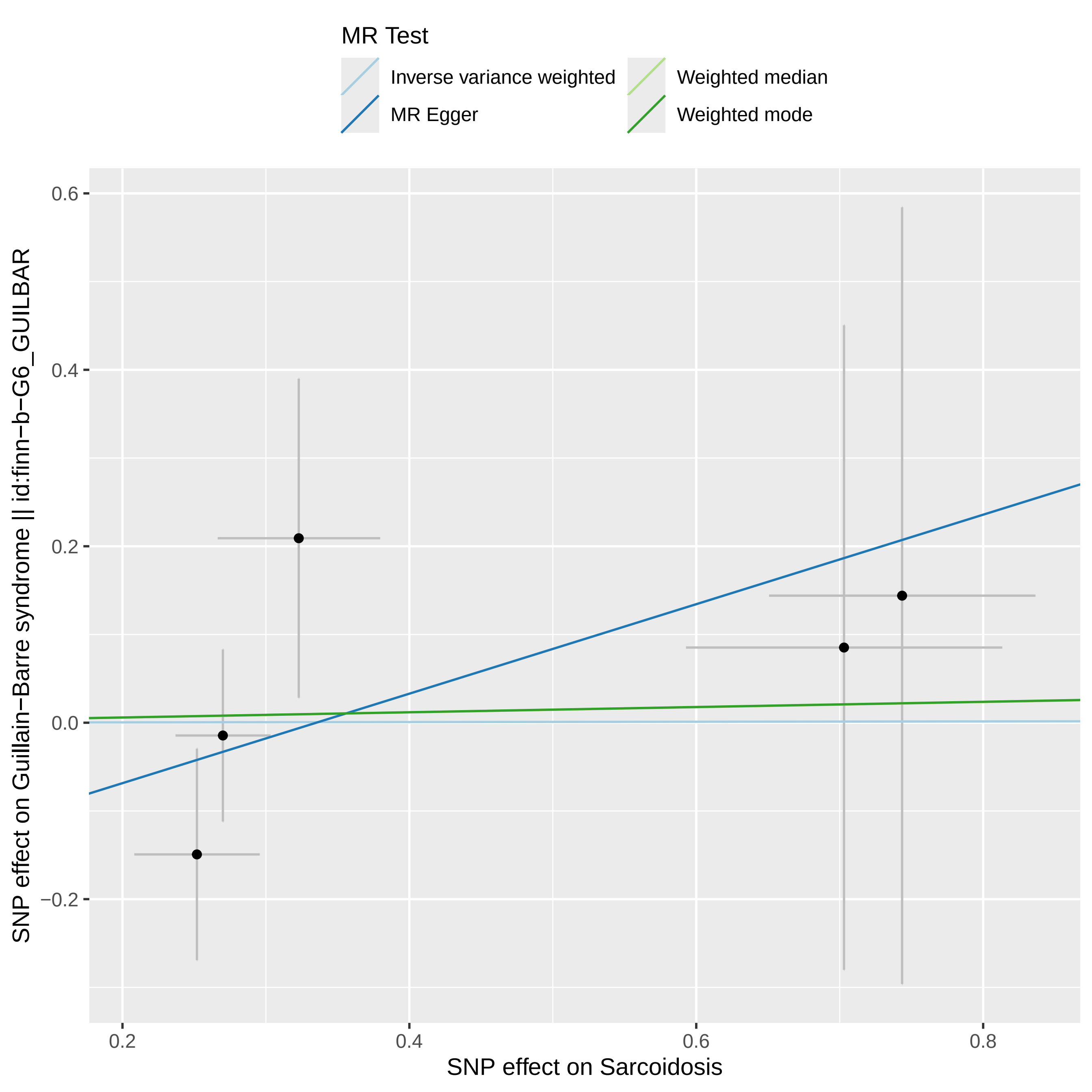
MR Test
Inverse variance weighted
MR Egger
Weighted median
Weighted mode
0.6
0.4
0.2
0.0
−0.2
0.2
0.4
0.6
0.8
SNP effect on Guillain−Barre syndrome || id:finn−b−G6_GUILBAR
SNP effect on Sarcoidosis

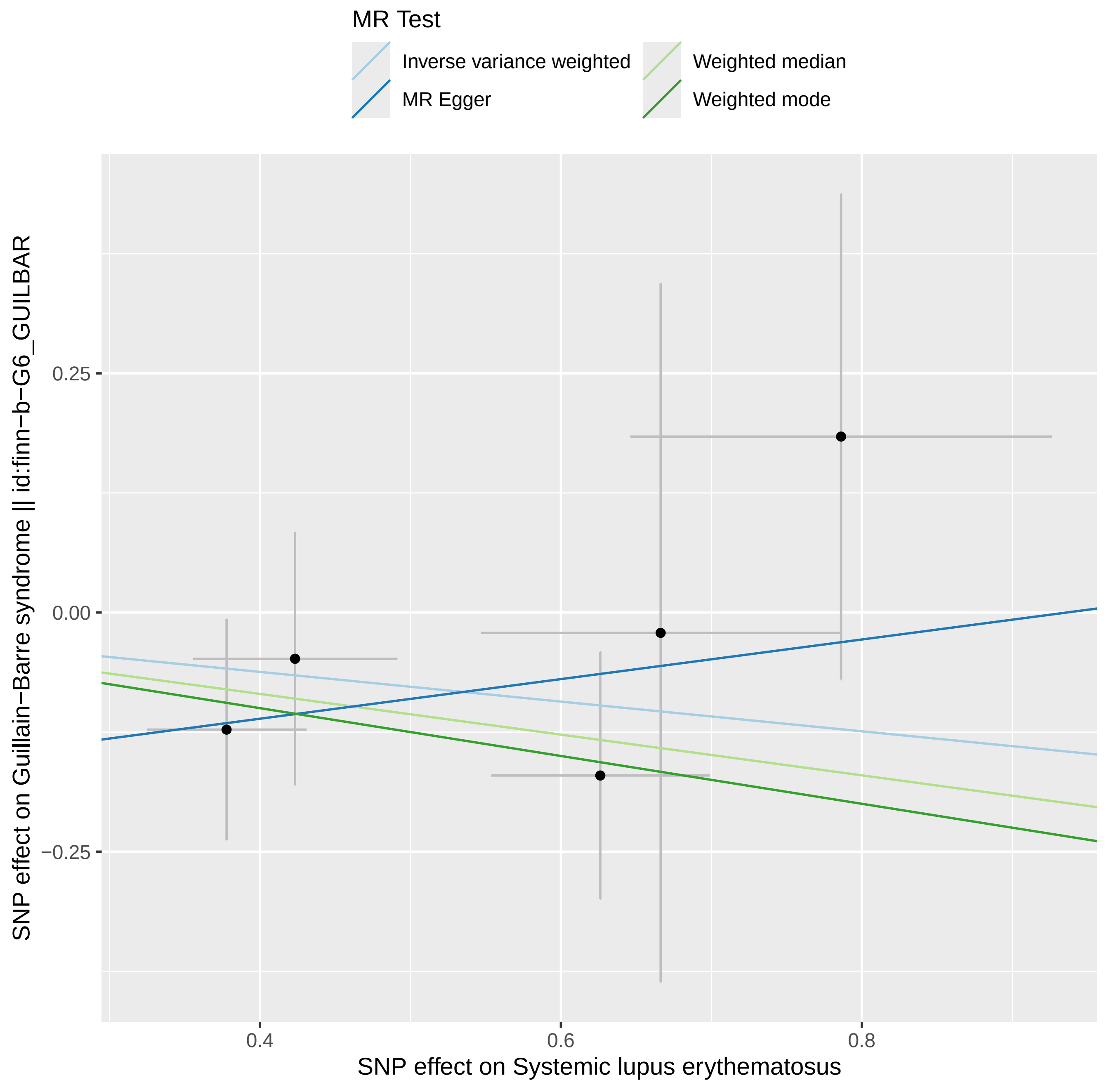
MR Test
Inverse variance weighted
MR Egger
Weighted median
Weighted mode
0.25
0.00
−0.25
0.4
0.6
0.8
SNP effect on Systemic lupus erythematosus
SNP effect on Guillain−Barre syndrome || id:finn−b−G6_GUILBAR

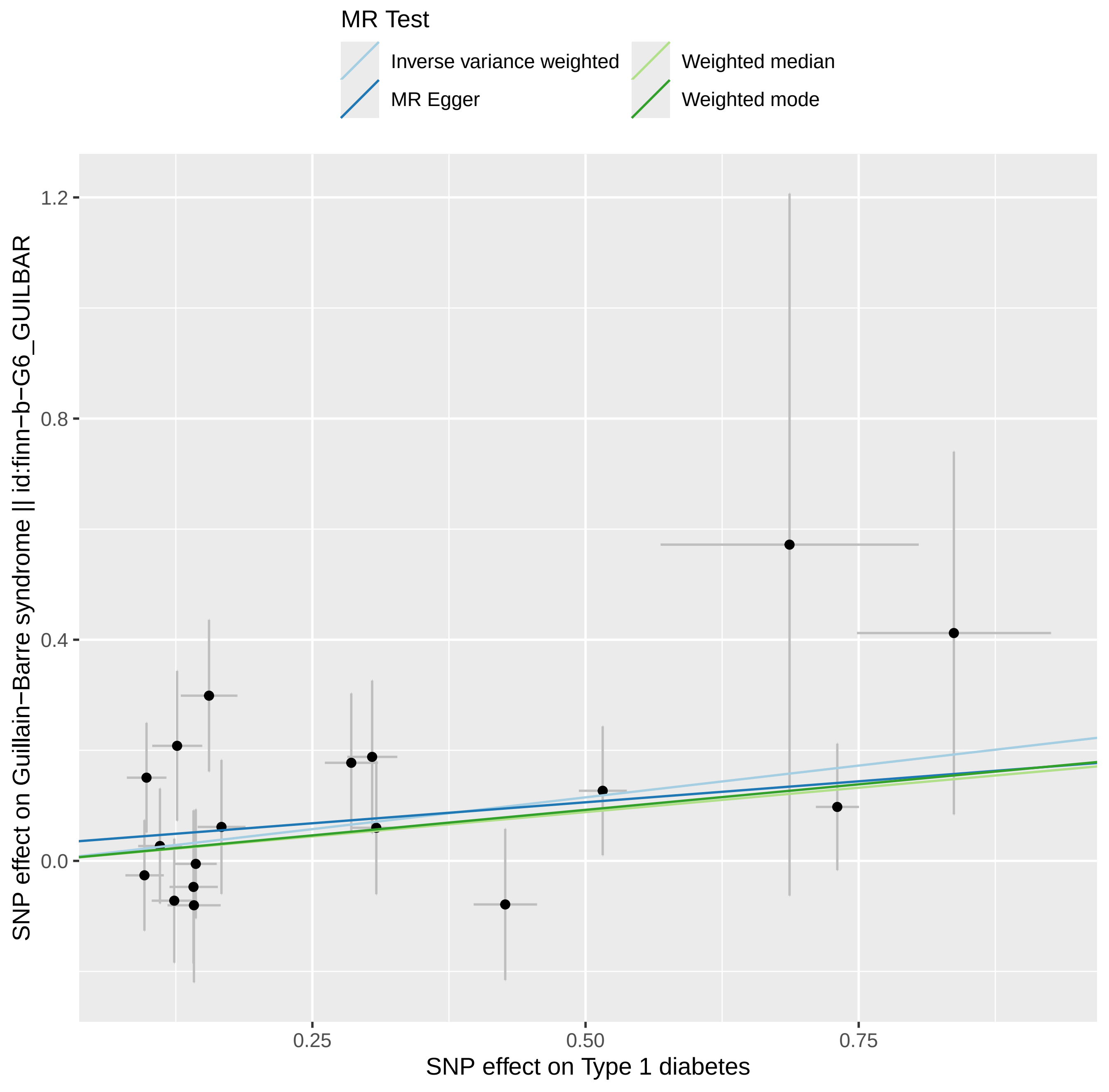

MR Test
Inverse variance weighted
MR Egger
Weighted median
Weighted mode
1.2
0.8
0.4
0.0
0.25
0.50
0.75
SNP effect on Guillain−Barre syndrome || id:finn−b−G6_GUILBAR
SNP effect on Type 1 diabetes
